# Human saliva modifies growth, biofilm architecture, and competitive behaviors of oral streptococci

**DOI:** 10.1128/msphere.00771-23

**Published:** 2024-02-06

**Authors:** Allen Choi, Kevin Dong, Emily Williams, Lindsey Pia, Jordan Batagower, Paige Bending, Iris Shin, Daniel I. Peters, Justin R. Kaspar

**Affiliations:** 1 Division of Biosciences, The Ohio State University College of Dentistry, Columbus, Ohio, USA; University of Wisconsin-Madison, USA

**Keywords:** intermicrobial interactions, biofilms, oral biology, microbial competition, human fluids, Streptococcus

## Abstract

The bacteria within supragingival biofilms participate in complex exchanges with other microbes inhabiting the same niche. One example is the mutans group streptococci (*Streptococcus mutan*s), implicated in the development of tooth decay, and other health-associated commensal streptococci species. Previously, our group transcriptomically characterized intermicrobial interactions between *S. mutans* and several species of oral bacteria. However, these experiments were carried out in a medium without human saliva. To better mimic their natural environment, we first evaluated how inclusion of saliva affected growth and biofilm formation of eight *Streptococcus* species individually and found saliva to positively benefit growth rates while negatively influencing biofilm biomass accumulation and altering spatial arrangement. These results carried over during evaluation of 29 saliva-derived isolates of various species. Surprisingly, we also found that addition of saliva increased the competitive behaviors of *S. mutans* in coculture competitions against commensal streptococci that led to increases in biofilm microcolony volumes. Through transcriptomically characterizing mono- and cocultures of *S. mutans* and *Streptococcus oralis* with and without saliva, we determined that each species developed a nutritional niche under mixed-species growth, with *S. mutans* upregulating carbohydrate uptake and utilization pathways while *S. oralis* upregulated genome features related to peptide uptake and glycan foraging. *S. mutans* also upregulated genes involved in oxidative stress tolerance, particularly manganese uptake, which we could artificially manipulate by supplementing in manganese leading to an advantage over its opponent. Our report highlights observable changes in microbial behaviors through leveraging environmental- and host-supplied resources over their competitors.

**IMPORTANCE:**

Dental caries (tooth decay) is the most prevalent disease for both children and adults nationwide. Caries are initiated from demineralization of the enamel due to organic acid production through the metabolic activity of oral bacteria growing in biofilm communities attached to the tooth’s surface. Mutans group streptococci are closely associated with caries development and initiation of the cariogenic cycle, which decreases the amount of acid-sensitive, health-associated commensal bacteria while selecting for aciduric and acidogenic species that then further drives the disease process. Defining the exchanges that occur between mutans group streptococci and oral commensals in a condition that closely mimics their natural environment is of critical need toward identifying factors that can influence odontopathogen establishment, persistence, and outgrowth. The goal of our research is to develop strategies, potentially through manipulation of microbial interactions characterized here, that prevent the emergence of mutans group streptococci while keeping the protective flora intact.

## INTRODUCTION

The bacteria that colonize the oral cavity live in complex communities within biofilms attached to the tooth’s surface. An array of complex intermicrobial interactions is present, with specific interactions developing to be synergistic, antagonistic, or neutral between species (1, 2). One well-studied antagonistic interaction occurs between mutans group streptococci and oral commensal streptococci (3, 4). Mutans group streptococci form microcolony structures that are encased and held structurally sound by glucan polysaccharides they abundantly produce (5–7). These species are also highly acidogenic, producing organic acids that are pooled within their microcolonies from fermentation of dietary carbohydrates, causing localized areas of acidic pH that demineralize the tooth’s enamel (8, 9). Oral commensal streptococci are commonly acid sensitive and encode various defense mechanisms, such as hydrogen peroxide production (10, 11) and arginine metabolism via the arginine deiminase (ADS) pathway (12, 13) to combat the outgrowth of the mutans group streptococci as well as to buffer against glycolytic acids. One favorable strategy toward tooth decay (dental caries) prevention is thwarting the emergence of mutans group streptococci within the oral microbiome while keeping the normal, protective microbiota intact. Development of new therapeutic approaches relies on fully characterizing these exchanges between competing microbes and unraveling the strategies employed by each species that leads to a gain in competitive advantage over the other.

Our group recently transcriptomically characterized coculture growth between *S. mutans* and several species of commensal streptococci (14). However, these experiments were carried out in our lab-based experimental medium, tryptone and yeast extract (TY-). *In vivo*, supragingival biofilms are constantly bathed in saliva. Resting human saliva contains organic and inorganic ions, peptides, and over 400 different host-derived proteins, many of which are glycosylated (15, 16). Individual free amino acids are present at concentrations less than 12 mg L^−1^ and the preferred carbohydrate glucose at a concentration less than 100 µM (17, 18). During periods of host fasting, oral microbes must obtain carbon, nitrogen, and energy from sources such as glycoproteins, requiring an assortment of enzymes that release oligosaccharides, peptides, and amino acids. These include several different classes of glycosidases and peptidases that are abundant throughout the genomes of oral bacteria but are often distributed between different species that leads to a consortium of different strains required to cooperatively degrade and liberate carbohydrate and peptide/amino acid moieties (19–21). For example, the glycan foraging activities of *Streptococcus oralis* are sufficient to digest the *N*-linked glycans of plasma human α_1_-acid glycoprotein (22) yet lack several glycoside hydrolases encoded by *Streptococcus mitis* and peptidases of *Streptococcus gordonii* to degrade proline-rich proteins (PRPs) (21, 23). Of note, the genome of the caries pathogen *Streptococcus mutans* does not contain many, if any glycoside hydrolases, and lacks PRP degradation activity (21, 23).

To understand whether competitive behaviors are altered by culturing these species within a medium that more closely mimics their natural environment, we evaluated cocultures of *S. mutans* and *S. oralis* in a TY-human saliva mix that was optimally chosen based on our initial characterization of individual oral streptococci growth in media mixes containing saliva. Our results show that inclusion of saliva enhances the competition of *S. mutans* against commensal streptococci, with each species segregating into their own nutritional niche.

## RESULTS

### Growth of oral streptococci are enhanced with human saliva

To examine how the growth of oral streptococci is altered in saliva, we first tested whether eight different strains could grow in only saliva and/or saliva supplemented with glucose (Fig. S1). None of the eight strains had measurable growth in saliva alone, while each strain, except for *Streptococcus* sp. A12, displayed low to moderate growth in the saliva containing glucose.

**Fig 1:**
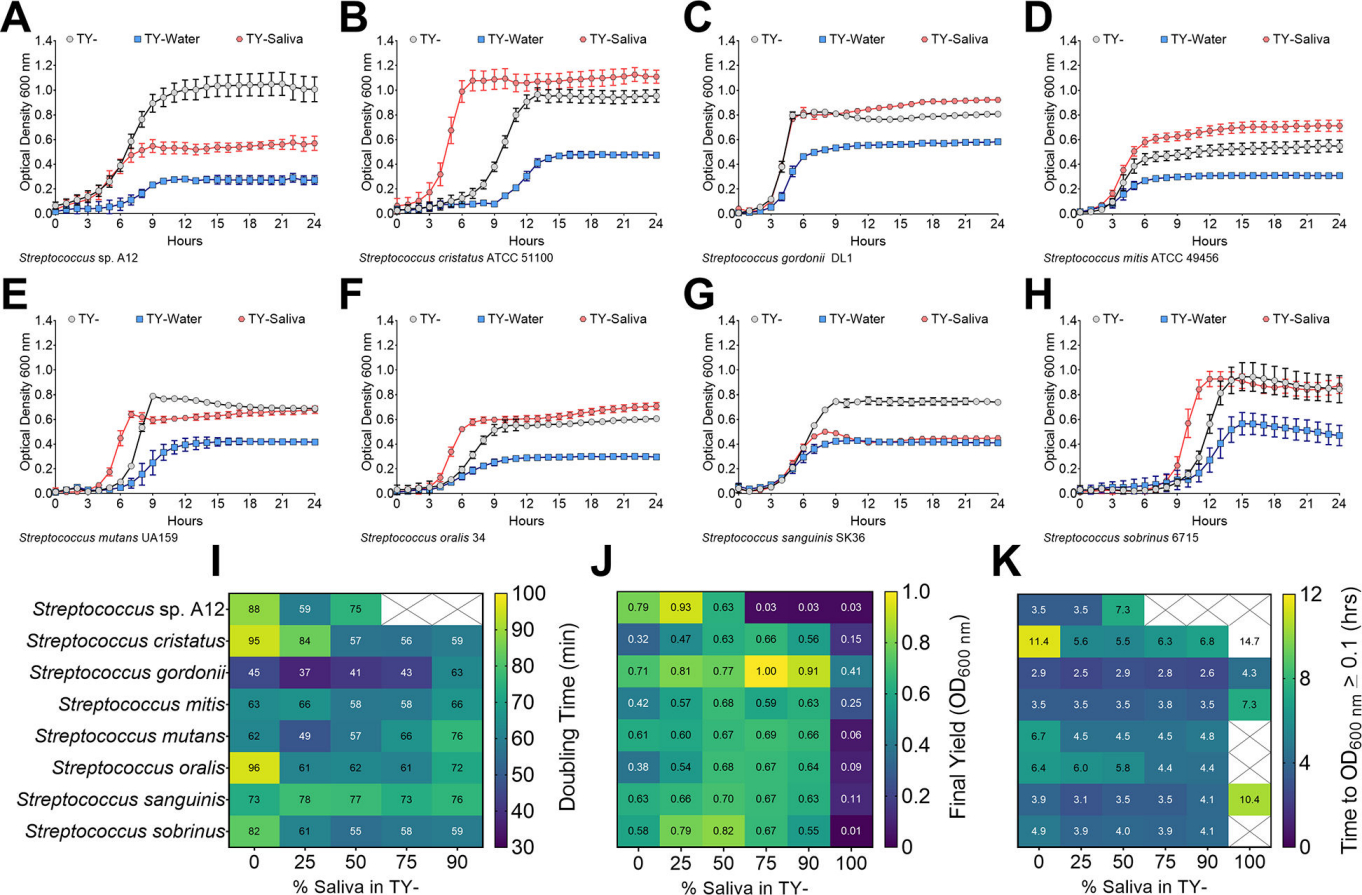
Growth of oral streptococci in human saliva. Growth curves of oral streptococci species (**A**) *S*. sp. A12, (**B**) *S. cristatus* ATCC 51100, (**C**) *S. gordonii* DL1, (**D**) *S. mitis* ATCC 49456, (**E**) *S. mutans* UA159, (**F**) *S. oralis* 34, (**G**) *S. sanguinis* SK36, and (**H**) *S. sobrinus* 6715 in either 100% TY (TY-; light grey circles), 50% TY / 50% H_2_O (TY-Water; blue squares), or 50% TY / 50% human saliva (TY-Saliva; red hexagons). Data points for optical density at 600 nm recorded every hour over a 24-h period are shown. Growth characteristic heat maps of the eight different oral streptococci with varying percentages (0%–100%) of human saliva mixed with TY. Characteristics shown include (**I**) doubling times (minutes), (**J**) final yield (OD_600 nm_), and (**K**) time to OD_600 nm_ ≥ 0.1 (hours). Dark-blue denotes lower values while green-yellow denotes the highest values within each heat map. White boxes crossed out without values indicate no growth in that condition.

An alternative strategy was chosen to mix our lab-based experimental medium, tryptone and yeast extract (TY-), half and half with either water (TY-Water, control) or saliva (TY-Saliva), and observe the growth of the same eight-species panel (Fig. 1A through H). The same amount of carbohydrate (20 mM glucose) was added into each medium. With this methodology, six of the eight strains had final yields at or even higher than TY-, and all eight had yields greater than optical density at 600 nm (OD_600 nm_) of 0.4 in medium containing saliva. Additionally, five of the eight strains showed improved growth in the TY-Saliva condition through either faster doubling times and/or shorter lag times.

We next wanted to determine the optimal amount of saliva to mix with TY- for the remainder of our experiments by recording doubling times, final yields, and lag times (Fig. 1I through K). Each strain saw improvements in at least one of these categories with 25%–75% saliva mixed in TY-. However, growth was impaired for some strains starting at 75% saliva and all strains with 90% saliva. Therefore, it was determined that a 50% TY-, 50% saliva mix would be used for all experiments going forward.

To ensure these measured growth enhancements with saliva were not TY- specific, we performed a similar set of growth assays in the chemically defined medium (CDM) (24–26) (Fig. S2). Here, all eight streptococci species displayed growth improvements over CDM alone with both *S. mitis* and *S. oralis* only growing in the CDM-Saliva condition. These data demonstrate that the growth of several oral streptococci species are improved in medias containing human saliva.

### Biofilm architecture is modified with human saliva

**Fig 2:**
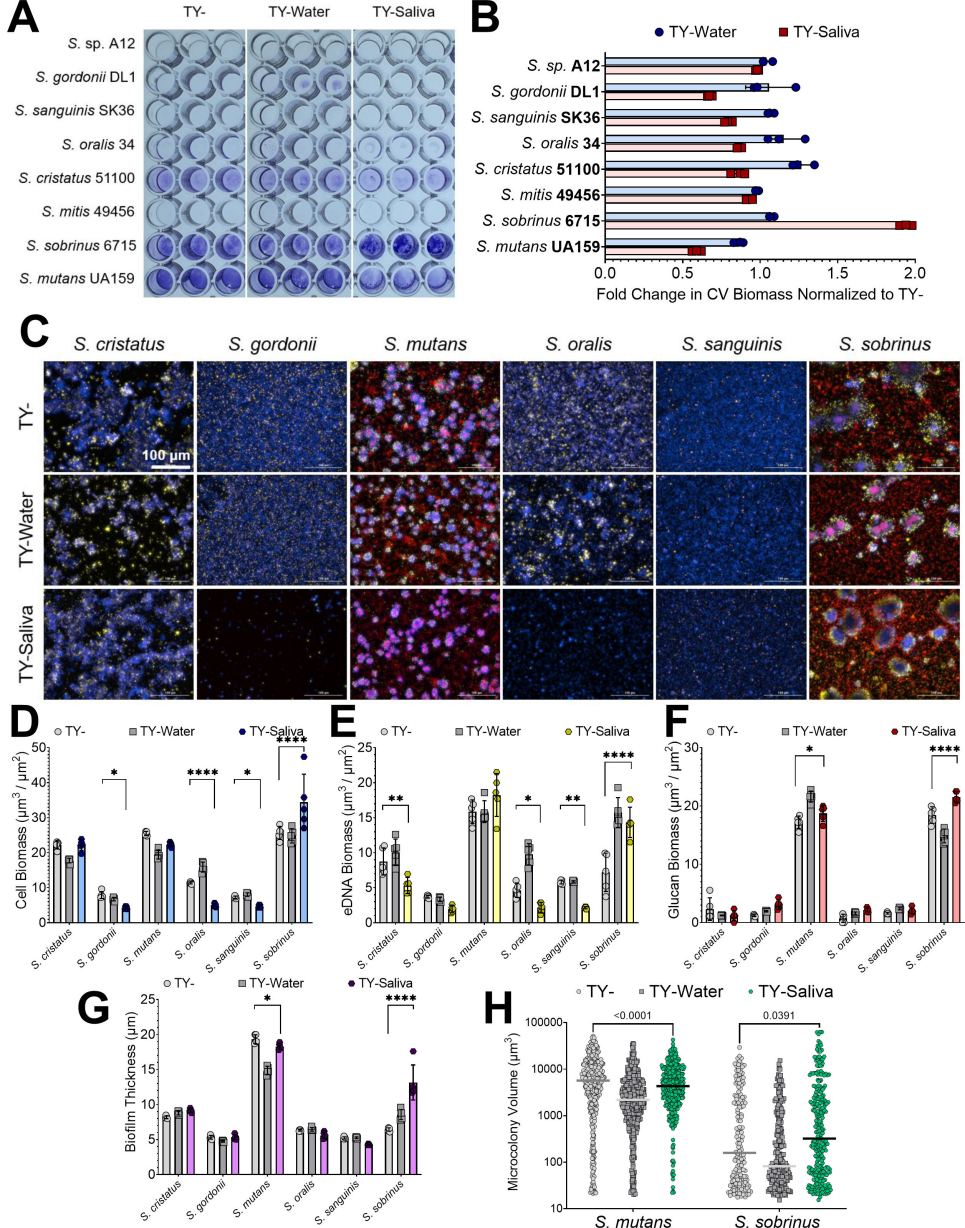
Biofilm formation of oral streptococci in human saliva. (**A**) Representative image of a crystal violet (CV) biofilm biomass assay for eight different oral streptococci species (listed on y-axis) in TY-, TY-Water, or TY-Saliva. (**B**) Fold change in CV biomass in TY-Water (blue circles) or TY-Saliva (red squares) normalized to TY- only. Data were quantified from the image shown on the left by extracting the crystal violet with 30% acetic acid and measuring the absorbance at 575 nm. (**C**) Merged representative maximum intensity 20× Z-projections of 24-h oral streptococci biofilms grown in either TY- (top), TY-Water (middle), or TY-Saliva (bottom). Bacterial cells were stained with Hoechst 33342 (blue), eDNA probed with labeled antibodies (yellow), and glucans visualized with labeled dextran (red). Scale bar (100 µm) is shown in top-left image. (**D**) Quantified cell, (**E**) eDNA, and (**F**) glucan biomass (µm^3^/µm^2^) along with (**G**) biofilm thickness (µm) and (**H**) individual microcolony volume from the image set shown in panel C between TY- (light-gray circles), TY-Water (dark-gray squares), or TY-Saliva (colored hexagons). Bars represent the mean of biomass or thickness from five independent images acquired (*n* = 5) with standard deviation. *S*. sp. A12 and *S. mitis* were not included within this panel, as they do not form adherent biofilms in monoculture. Quantification was completed using Gen5 Image+ software. Data graphing and two-way analysis of variance (ANOVA) with multiple comparisons were completed in GraphPad Prism software. * *P* < 0.05, **P < 0.01, ***P < 0.001, and ****P < 0.0001.

Previous studies have shown that salivary mucins can reduce oral streptococci biofilm formation (27, 28). To determine if similar reductions occur with saliva, we first compared the 24-h accumulated biomass using a crystal violet assay (Fig. 2A and B). All seven species, except for *Streptococcus sobrinus*, had similar or lower quantifiable biomass with the addition of saliva. Interestingly, *S. sobrinus* nearly doubled its accumulated biomass in TY- containing saliva.

To better understand compositional and structural changes that may be occurring, we imaged and quantified single-species biofilms in our three different growth media (Fig. 2C through H). We observed a significant reduction in *S. gordonii*, *S. oralis*, and *Streptococcus sanguinis* cell biomass along with a decline in *Streptococcus cristatus*, *S. oralis*, and *S. sanguinis* eDNA biomass in TY-Saliva compared with TY-. However, for *S. sobrinus*, a significant increase in cell, eDNA, and glucan biomass was recorded. Overall, human saliva alters biofilm accumulation and architecture of oral streptococci species while enhancing biofilm formation of an odontopathogen (*S. sobrinus*).

### Similar changes in growth and biofilm formation with saliva-derived bacterial isolates

**Fig 3:**
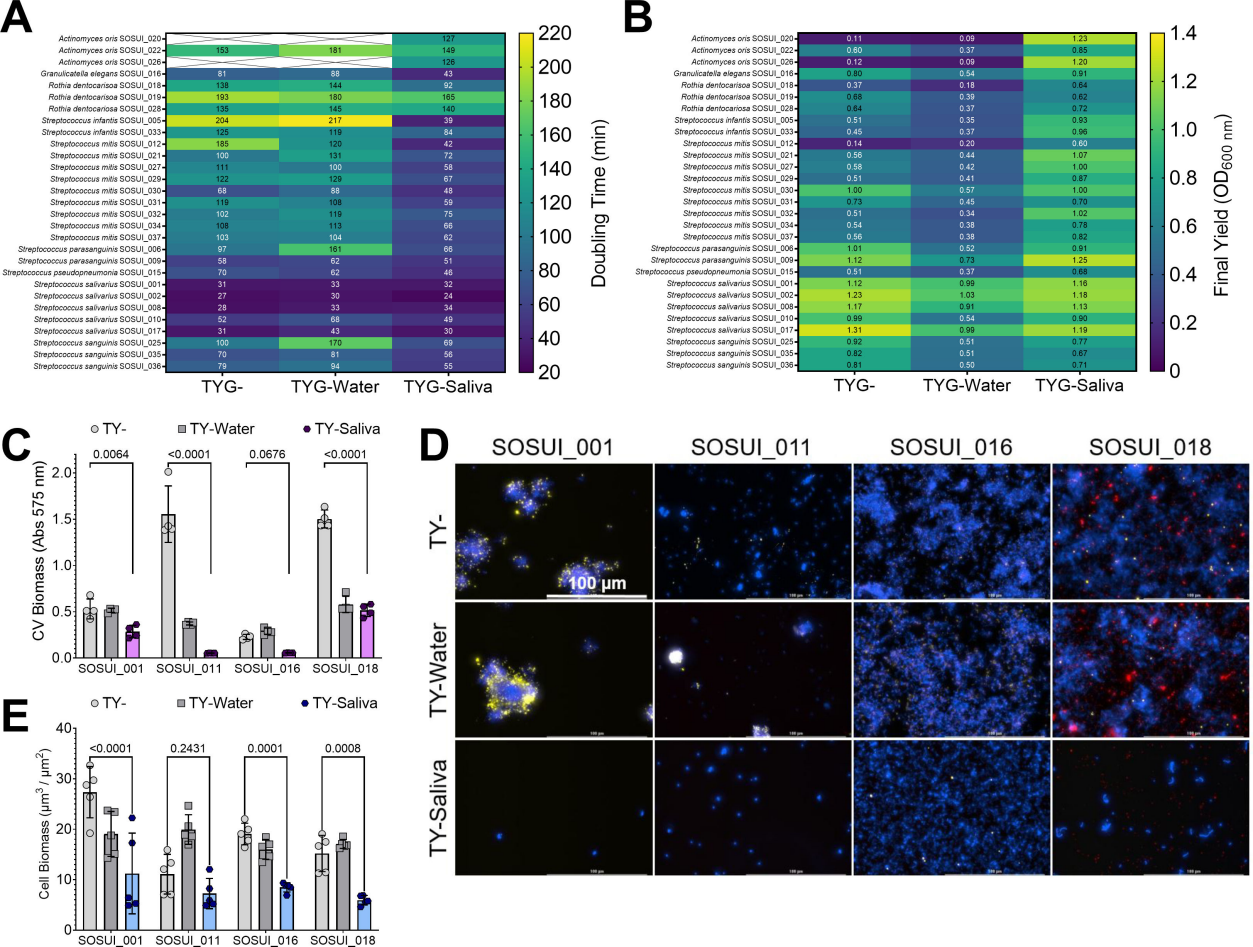
Growth and biofilm formation of human saliva-derived bacterial isolates. Growth characteristic heat maps of (**A**) doubling times (minutes) and (**B**) final yield (OD_600 nm_) shown for 29 different human saliva-derived bacterial isolates. Their SOSUI identifier #, along with species identity, is shown on the left y-axis. Dark-blue denotes lower values while green-yellow denotes the highest values within each heat map. White boxes crossed out without values indicate no growth in that condition. (**C**) CV biomass (absorbance at 575 nm) of SOSUI_001, _011, _016, and _018 in TY- (light-gray circles), TY-Water (dark-gray squares), or TY-Saliva (purple hexagons). *n* = 4. (**D**) Merged representative maximum intensity 40× Z-projections of 24-h saliva isolate biofilms grown in either TY- (top), TY-Water (middle), or TY-Saliva (bottom). Bacterial cells were stained with Hoechst 33342 (blue), eDNA probed with labeled antibodies (yellow), and glucans visualized with labeled dextran (red). Scale bar (100 µm) is shown in top-left image. (**E**) Quantified cell biomass (µm^3^/µm^2^) from the image set shown in panel D between TY- (light-gray circles), TY-Water (dark-gray squares), or TY-Saliva (colored hexagons). *n* = 5. Quantification was completed using Gen5 Image+ software. Data graphing and two-way ANOVA with multiple comparisons were completed in GraphPad Prism software with resulting *P* values displayed.

To verify that documented changes were not limited to lab-adapted strains, we first isolated 29 different bacterial colonies that grew on Brain Heart Infusion (BHI) agar plates from our commercially sourced saliva. Viable colonies were streaked for isolation and then species identified by 16S sequencing. Identities returned included a range of different *Streptococcus*, *Actinomyces*, *Rothia*, and *Granulicatella* species. We then monitored the growth of each isolate. Doubling times for 26 out of 29 isolates were quickest in the TY-Saliva condition, and 18 out of 29 recorded their highest final yields as well (Fig. 3A and B). Of note, two *Actinomyces oris* isolates (SOSUI_020 and SOSUI_026) and a *S. mitis* isolate (SOUSI_012) struggled to grow in either of the TY- and TY-Water conditions but grew well in medium that contained saliva. To measure potential changes in biofilm formation, we first screened 17 of the isolates (SOSUI_001 – SOSUI_018, and SOSUI_003 failed to grow after isolation and were excluded from all experiments) for measurable biomass accumulation (Fig. S3). Four isolates, SOSUI_001, SOSUI_011, SOSUI_016, and SOSUI_018, were selected to carry forward for further analysis (each isolate also represented a different genus). Three of the four isolates saw significant reductions in biomass accumulation in TY-Saliva compared with TY- while the fourth, SOSUI_16, saw a non-significant 75% ± 1% reduction (Fig. 3C). Similar patterns of reduction in both cell biomass and biofilm matrix components could be visualized by microscopy (Fig. 3D and E). These data suggest that observations made with lab-adapted oral streptococci species are similar to phenotypes displayed by low-passage saliva-derived isolates including non-*Streptococcus* species.

### Saliva enhances the competitive behaviors of *S. mutans*

**Fig 4:**
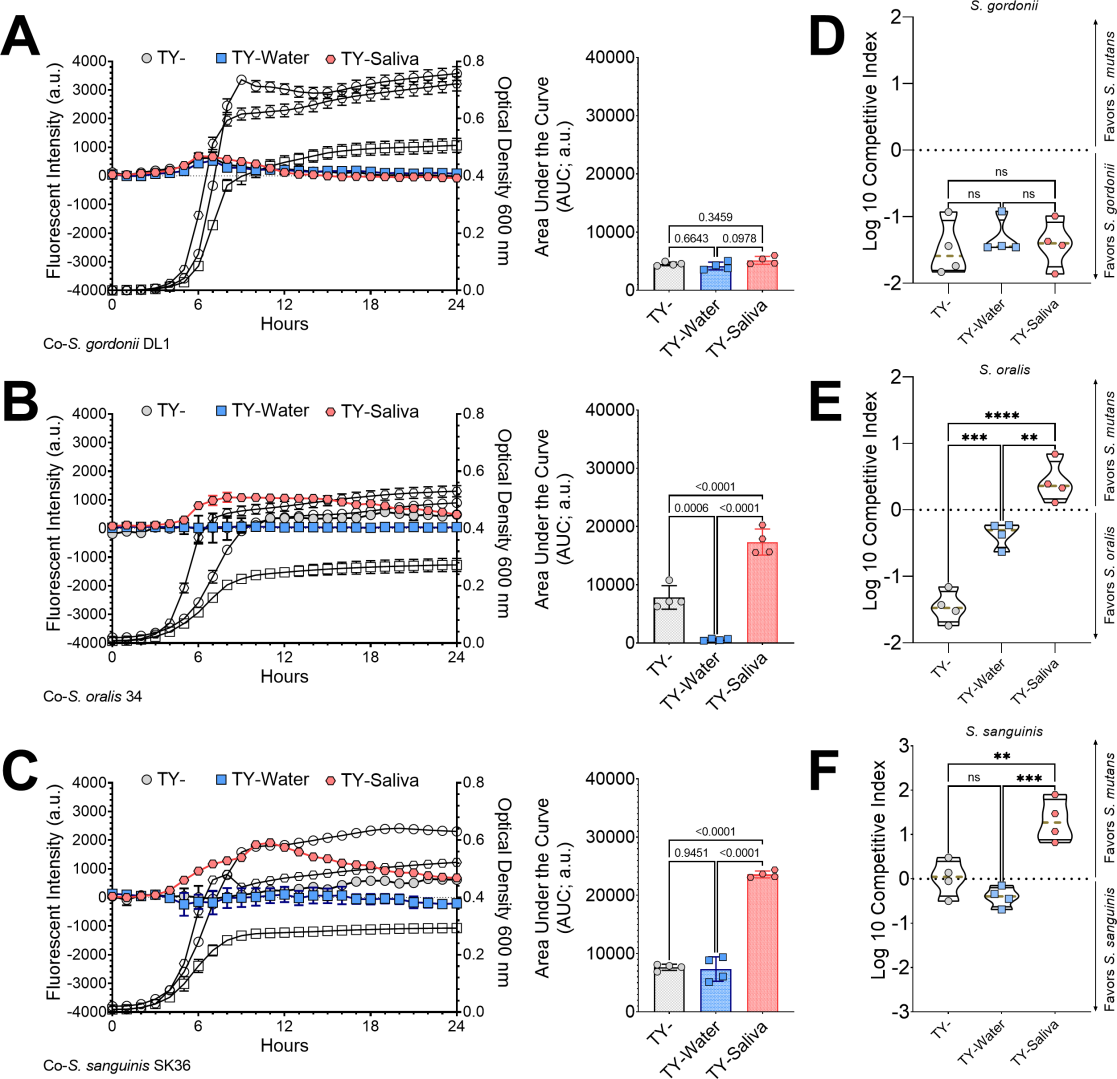
Competition assays between *S. mutans* and commensal streptococci competitors. Fluorescence-based [intensity, arbitrary units (a.u.), left y-axis] growth profile of *S. mutans* in coculture competition against (**A**) *S. gordonii* DL1, (**B**) *S. oralis* 34, and (**C**) *S. sanguinis* SK36 in either TY- (light-gray circles), TY-Water (blue squares), or TY-Saliva (red hexagons). Growth of the entire coculture, measured by optical density at 600 nm, is shown in black outline using the same symbol (right y-axis). The AUC of the fluorescent intensity of each condition was quantified and is shown in the graph on the right (*n* = 4). Violin plot of Log10 competitive index of *S. mutans* in coculture competition against (**D**) *S. gordonii* DL1, (**E**) *S. oralis* 34, and (**F**) *S. sanguinis* SK36 in either TY- (light-gray circles), TY-Water (blue squares), or TY-Saliva (red hexagons). Positive values represent a competitive advantage for *S. mutans* while negative values represent a competitive advantage for the commensal strain. Black solid lines represent the quartiles while a gold dashed line represents the median. *n* = 4. Data graphing and one-way or two-way ANOVA with multiple comparisons were completed in GraphPad Prism software. * *P* < 0.05, **P < 0.01, ***P < 0.001, and ****P < 0.0001.

Previous studies reported that salivary mucins can alter the competitive behaviors between *Streptococcus mutans* and commensal streptococci (29). We performed a fluorescent intensity-based coculture competition assay between a chromosomally integrated, constitutively expressing *S. mutans gfp* strain (30) and unmarked competitor species (Fig. 4A through C). Specific growth of *S. mutans* could be tracked via fluorescent intensity measurements of green fluorescent protein (GFP) every half hour over a 24-h period, while the optical density of the entire coculture was also recorded. An area under the curve (AUC) was calculated using the intensity measurements. A significant increase in *S. mutans* intensity was recorded in cocultures with *S. oralis* and *S. sanguinis* but not with *S. gordonii* within the TY-Saliva condition. To verify that the fluorescent intensity-based competition led to quantifiable changes in recoverable cell number, we set up a competitive index assay between *S. mutans* and the three commensal strains that enumerated colony-forming units (CFUs) during inoculation (*t*_i_ = 0 h) and at the time of cell harvest (*t*_f_ = 24 h) (Fig. 4D through F). The competitive index assay showed similar results to the fluorescent intensity-based assay, with *S. mutans* displaying a higher competitive index in TY-Saliva compared with TY- with *S. oralis* and *S. sanguinis*, but no change with *S. gordonii*. We also used the fluorescent intensity-based competition with other competitors and found higher *S. mutans* intensities in TY-Saliva with *S*. sp. A12, *S. cristatus*, and *S. sobrinus*, but not with *S. mitis* (Fig. S4). *S. mutans* additionally displayed increased intensity within cocultures using CDM-, ensuring this change was not specific for TY- (Fig. S5).

**Fig 5:**
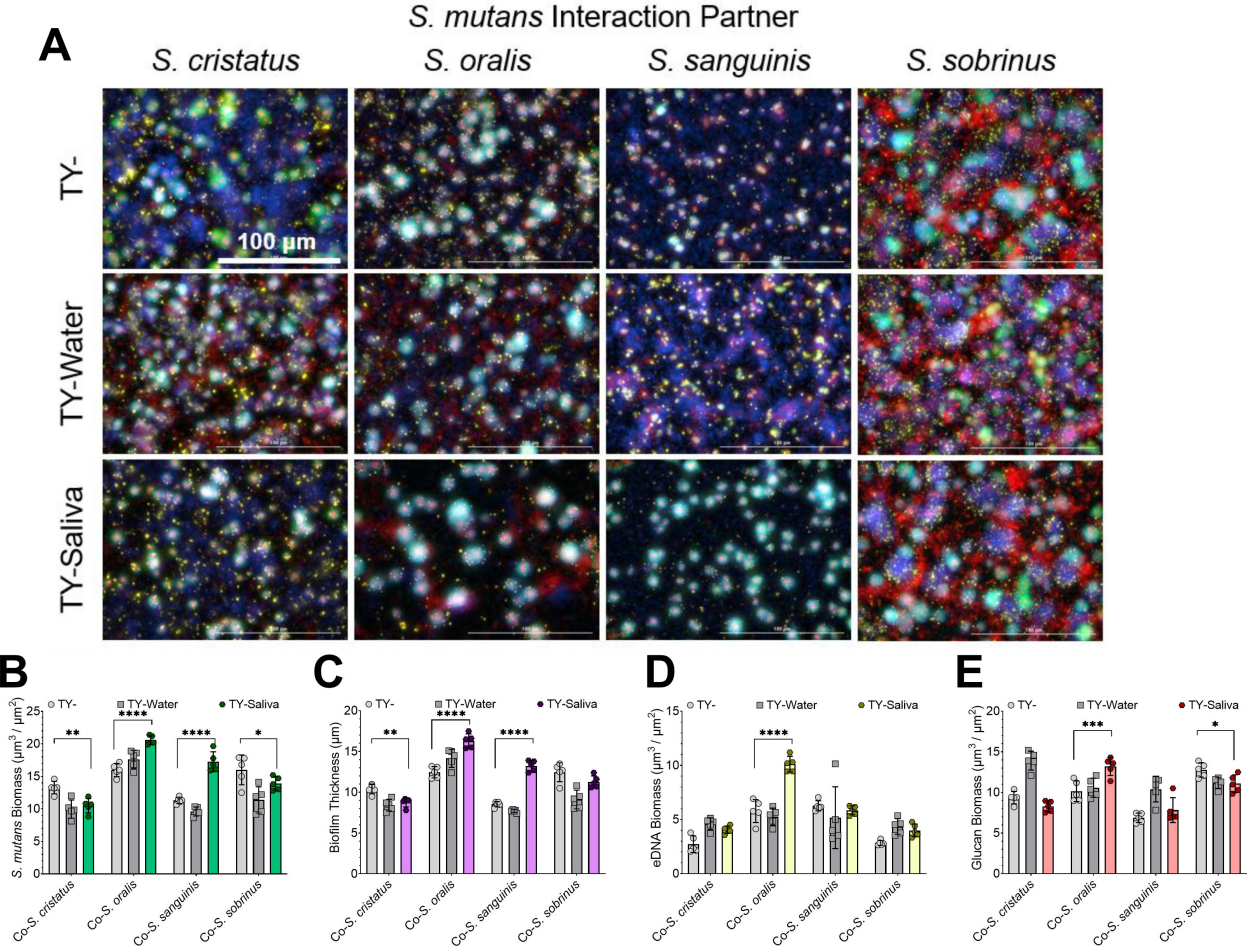
Increased biomass of *S. mutans* within human saliva. (**A**) Merged representative maximum intensity 40× Z-projections of 24-h cocultured biofilms between a *S. mutans* GFP+ strain (green) and oral commensal competitors (stained with Hoechst 33342, blue). Biofilm matrix component eDNA was probed with labeled antibodies (yellow) and glucans visualized with labeled dextran (red). (**B**) Quantified *S. mutans* biomass (µm^3^/µm^2^), (**C**) biofilm thickness (µm), (**D**) eDNA, and (**E**) glucan biomass from the image set shown in panel B between TY- (light-gray circles), TY-Water (dark-gray squares), and TY-Saliva (colored hexagons). Bars represent the mean of biomass or thickness from five independent images acquired (*n* = 5) with standard deviation. Quantification was completed using Gen5 Image+ software. Data graphing and one-way or two-way ANOVA with multiple comparisons were completed in GraphPad Prism software. * *P* < 0.05, **P < 0.01, ***P < 0.001, and ****P < 0.0001.

For additional validation, we also examined 24 coculture biofilms and found significantly higher quantified *S. mutans* biomass with *S. oralis* and *S. sanguinis* that increased overall biofilm thickness (Fig. 5). Together, these data indicate that human saliva can enhance the competitive behaviors of *S. mutans* during growth with some commensal species.

### Human saliva reprograms the transcriptome of oral streptococci

**Fig 6:**
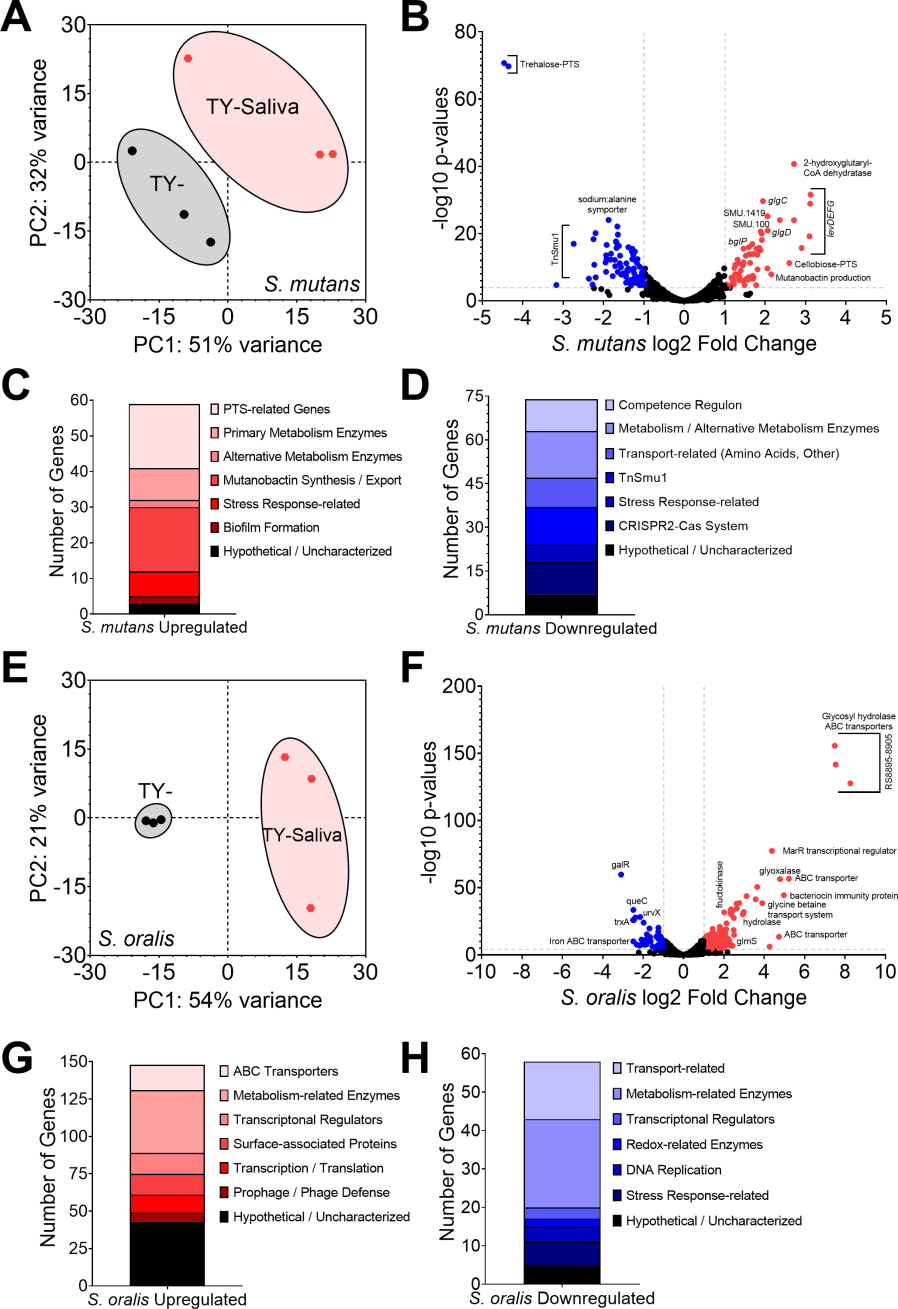
Transcriptome profiling of *S. mutans* and *S. oralis* monocultures in human saliva. (**A**) Principal component analysis (PCA) from RNA-Seq expression data (*n* = 3) of *S. mutans* monocultures grown in TY- (black circles) or TY-Saliva (red hexagons). The proportion of variance for either PC1 (x-axis) or PC2 (y-axis) is listed. (**B**) Volcano plot of changes within individual *S. mutans* genes (circles) between TY- and TY-Saliva. Differentially expressed genes (genes with >4 Log10 *P* value and Log2 fold change > (−)1) are shown in either red (upregulated, right) or blue (downregulated, left). Individual gene identifier, name, and/or characterized function are displayed, if able. (**C**) Stacked bar chart of upregulated *S. mutans* DEGs grouped by pathway/operon/function. (**D**) Stacked bar chart of downregulated *S. mutans* DEGs. (**E**) PCA from RNA-Seq expression data of *S. oralis* monocultures (*n* = 3). (**F**) Volcano plot of changes within individual *S. oralis* genes. (**G**) Stacked bar chart of upregulated *S. oralis* DEGs. (**H**) Stacked bar chart of downregulated *S. oralis* DEGs. DEGs were determined from Degust using edgeR analysis. Data graphing and PCA calculations were completed in GraphPad Prism software.

To understand how saliva affects changes in growth, biofilm formation, and competition between the oral streptococci, we performed RNA-Seq on monocultures of *S. mutans* and *S. oralis* in TY-Saliva and compared with TY- (Fig. 6). We found 133 differentially expressed genes (DEGs) in *S. mutans* grown in saliva (Table S1), with the most upregulated genes (59 total DEGs) belonging to phosphotransferase systems (PTS) specific for fructose, mannose and/or N-acetyl glucosamine (GlcNAc) (*levDEFGX*, SMU.1956c – SMU.1961c) (31–33), cellobiose (SMU.1596 – SMU.1600) (34), and α−1,3-linked carbohydrates (SMU.100 – SMU.105) (35). Other upregulated genes of note included the glucosyltransferases *gtfBC* (SMU.1004, SMU.1005), the bacitracin resistance ABC transporters *mbrAB* (SMU.1006-SMU.1007) (36), and the TnSmu2 gene cluster which contains genes for mutanobactin synthesis (37). In contrast, many of the downregulated genes (74 DEGs) belonged to the integrative and conjugative element TnSmu1 (38, 39), the genetic competence regulon (40), a CRISPR2-Cas system (41, 42), and predicted amino acid ABC transporters (SMU.932–SMU.936). Interestingly, the most downregulated genes within the data set belonged to the trehalose-specific PTS (43, 44). The commensal *S. oralis* had a higher number of DEGs overall (206 total, 11% of coding genome features) (Table S2), consisting of upregulation (148 DEGs) in 18 different ABC transporters, 13 transcriptional regulators, 7 glycosyl hydrolases, and genes involved in *de novo* purine and tryptophan biosynthesis. Downregulated genes (58 DEGs) included queuosine biosynthesis (45), transporters for iron (46), vitamin B12 (47), and glutamine (48) and transcriptional regulators *ciaR* (49) and *mntR* (50). These described DEGs highlight the alternations in metabolic networks for oral streptococci during growth in saliva.

**Fig 7:**
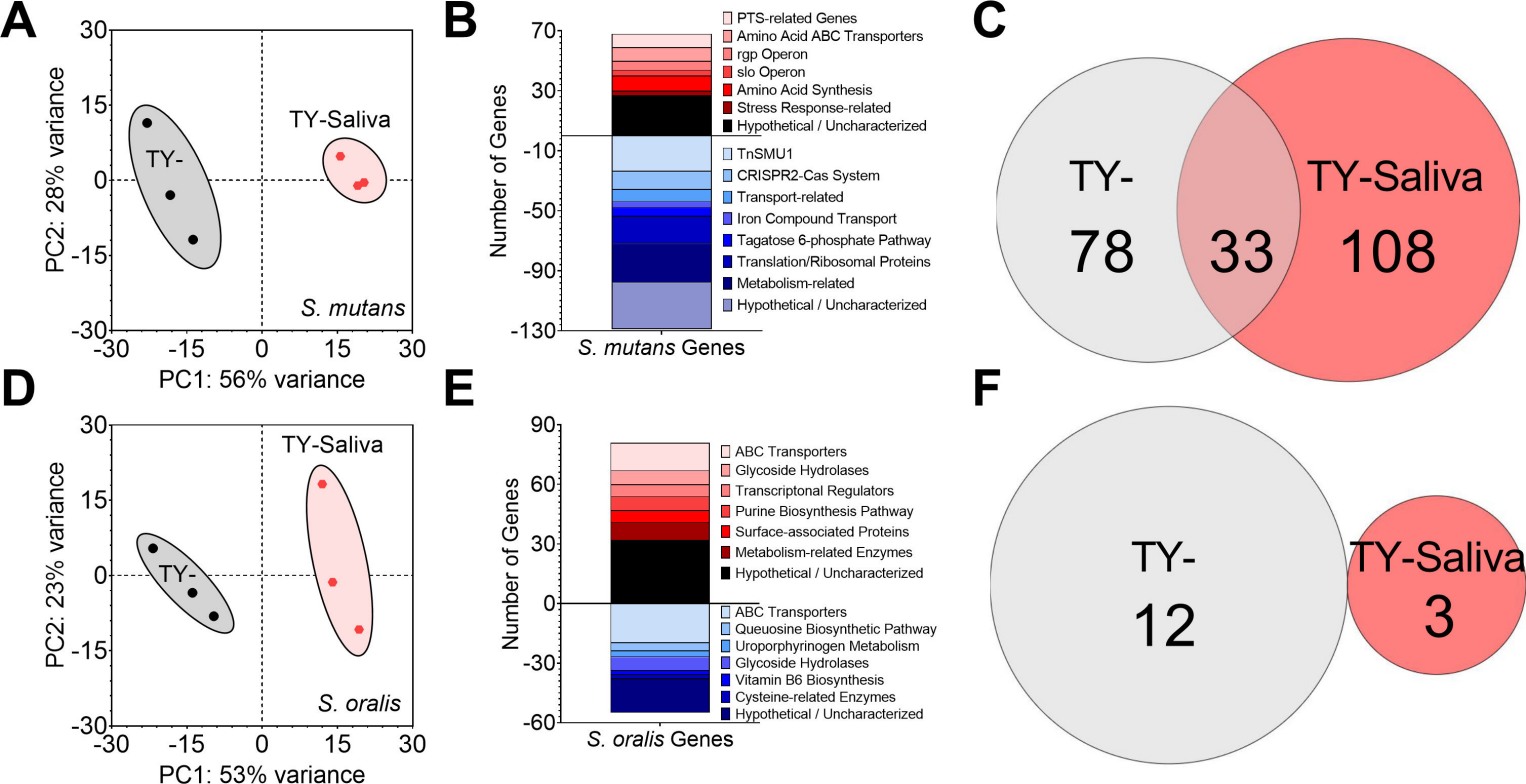
Transcriptome profiling of *S. mutans* and *S. oralis* cocultures in human saliva. (**A**) PCA from RNA-Seq expression data (*n* = 3) of *S. mutans* cocultures with *S. oralis* grown in TY- (black circles) or TY-Saliva (red hexagons). The proportion of variance for either PC1 (x-axis) or PC2 (y-axis) is listed. (**B**) Stacked bar chart of *S. mutans* upregulated (red, positive) and downregulated (blue, negative) DEGs grouped by pathway/operon/function. (**C**) Venn diagram of the number of *S. mutans* DEGs in coculture with *S. oralis* specific to TY- (light-gray), TY-Saliva (red), or common to both conditions (overlap). (**D**) PCA from RNA-Seq expression data of *S. oralis* cocultures with *S. mutans* (*n* = 3). (**E**) Stacked bar chart of *S. oralis* upregulated and downregulated DEGs. (**F**) Venn diagram of the number of *S. oralis* DEGs in coculture with *S. mutans* specific to each condition.

We also compared DEGs of both *S. mutans* and *S. oralis* grown in coculture TY-Saliva to coculture TY- (Fig. 7). By already characterizing monocultures in saliva, we were able to categorize DEGs that overlapped with monoculture growth as well as isolate new features that were specific only to the coculture interaction. For *S. mutans*, this included upregulation of transporters related to manganese (*slo* operon and *mntH*) (51), maltose (*malXFGK*) (52), glutamine and glutathione uptake (SMU.1939c-SMU.1942c) (53), and the rhamnose-glucose cell wall polysaccharide (*rgp*) operon (54, 55) (Table S3). Additionally, the trehalose-specific PTS, the most downregulated genes in monoculture, were now upregulated twofold in coculture.

Recently, our group highlighted a repetitive DEG pattern for *S. mutans* during coculture growth compared with monoculture, including with *S. oralis* (14). To determine how saliva modified this pattern, we compared *S. mutans* DEGs during transition from monoculture to coculture growth with *S. oralis* in either TY- or TY-Saliva (i.e., DEGs from coculture in TY- vs DEGs from coculture in TY-Saliva). Of the 219 total *S. mutans* DEGs captured during coculture growth with *S. oralis* in both media conditions, 78 were specific to TY-, 108 were specific to TY-Saliva, and 33 were present under both conditions (Table S4). These 33 genome features consisted of the CRISPR2-Cas system, *levDEFGX*, the transglycosylase SMU.2146c, the LexA-like transcriptional regulator *hdiR* (SMU.2027), and amino acid ABC transporters SMU.932-SMU.936. For *S. oralis*, only 24 DEGs were specific to the coculture interaction (Table S5). Eleven were upregulated and included genes within the tagatose 6-phosphate pathway, an endo-α-*N*-acetylgalactosaminidase (56), an *N*-acetylmannosamine kinase (57) and α-mannosidase, and a thiamin transporter. Downregulated genes included *oppA*, other amino acid transporters, and the uroporphyrinogen metabolism pathway. For comparison of DEGs during transition from monoculture to coculture, we found only 15 total DEGs with no overlap between conditions (Table S6).

### *S. mutans* growth enhanced with alternative carbohydrates and manganese

**Fig 8:**
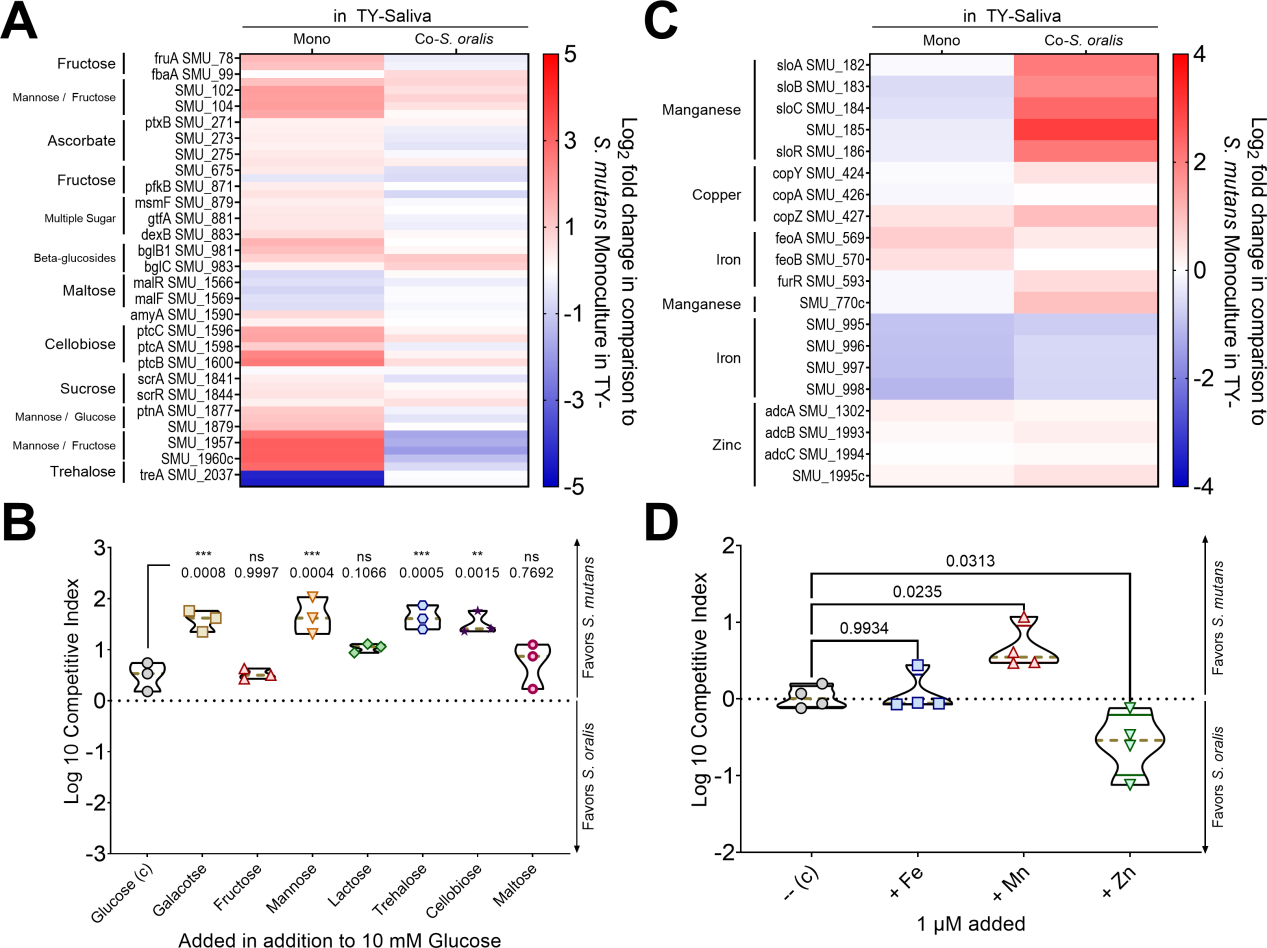
Altering the competitive behaviors of *S. mutans* with alternative carbohydrate sources or metals. (**A**) Log2 fold change heat map of *S. mutans* carbohydrate uptake gene expression changes in TY-Saliva monoculture or coculture with *S. oralis* 34 in comparison to TY- monoculture growth. Deeper red colors denote upregulation while deeper blue colors denote downregulation. (**B**) Violin plot of Log10 competitive index of *S. mutans* in coculture competition against *S. oralis* 34 in TY-Saliva with 10 mM glucose and 10 (monosaccharides) or 5 (disaccharides) mM of the indicated carbohydrate. Positive values represent a competitive advantage for *S. mutans* while negative values represent a competitive advantage for *S. oralis*. Black solid lines represent the quartiles while a gold dashed line represents the median. *n* = 4. (**C**) = control. (**C**) Log2 fold change heat map of *S. mutans* metal uptake gene expression changes. (**D**) Violin plot of Log10 competitive index of *S. mutans* in coculture competition against *S. oralis* 34 in TY-Saliva with 1 µM addition of the indicated metal (FeSO_4_, MnSO_4_, or ZnSO_4_). Data graphing and one-way or two-way ANOVA with multiple comparisons were completed in GraphPad Prism software. * *P* < 0.05, **P < 0.01, ***P < 0.001, and ****P < 0.0001.

A striking feature from our transcriptomic data sets was the set of DEGs in *S. mutans* carbohydrate uptake and utilization pathways in TY-Saliva that is altered if *S. mutans* is in monoculture or coculture with *S. oralis* (Fig. 8A). One hypothesis is that *S. mutans* and *S. oralis* could be segregating into different preferred nutritional niches that is enhanced with the inclusion of saliva into the growth medium. To begin to test this hypothesis, we performed a competitive index assay between *S. mutans* and *S. oralis* in TY-Saliva that included 10 mM glucose as well as 10 mM of an additional monosaccharide (galactose, fructose, or mannose) or 5 mM of an additional disaccharide (lactose, trehalose, cellobiose, or maltose). Each strain grew similarly in monoculture between the different medias (Fig. S6). However, in coculture, the presence of galactose, mannose, trehalose, and cellobiose increased the competitiveness of *S. mutans* while inclusion of fructose, lactose, and maltose saw no significant change from growth in glucose alone (Fig. 8B). Similarly to carbohydrates, metal uptake pathways were listed as DEGs in coculture with *S. oralis* (Fig. 8). Supplementing the media with 1 µM addition of iron (Fe) saw no change in competitive index, but addition of manganese (Mn) benefited *S. mutans* while zinc (Zn) shifted the balance in favor of *S. oralis* (Fig. 8D). Together, these data indicate that studying observable changes in DEGs could offer opportunities to further define the interactions that occur between species, while also opening interventional strategies to artificially manipulate interaction outcomes.

## DISCUSSION

Our foray into examining the growth and competition of oral streptococci in saliva began with the goal of documenting how the interaction(s) between *S. mutans* and commensal streptococci are modified in the presence of saliva. We were able to verify prior reports that most streptococcal strains are not able to grow in 100% saliva unless glucose is added as a carbon source (58). Even then, *S. gordonii* was the only species that achieved growth levels that would have been suitable for our assays, which also follows previous observations (59). Therefore, we used the strategy of mixing our lab-based experimental medium, TY-, with different proportions of saliva. Conveniently, mixing an equal volume of TY- with saliva led to growth benefits for almost all eight species of streptococci tested. Including even just small amounts of saliva led to growth benefits, with *S. oralis* recording faster doubling times in TY- with as little as 1% saliva added (Fig. S7). One surprise was finding several species that clearly grew better with the addition of saliva such as *S. cristatus* and *S. oralis*, with saliva even allowing for growth of *S. oralis* and *S. mitis* in CDM. While this experimental protocol does not fully replicate conditions *in vivo*, it does allow for inclusion of important host-derived factors that can influence the growth of oral bacteria. Other models that take into consideration more factors of the oral environment than our protocol tested here may provide further insights into oral bacteria growth. While we have not yet determined the factor(s) present in saliva that are promoting growth, the displayed phenotypes should provide a clear test to determine their identity. These discoveries could also be translatable to other genera, as similar growth-promoting phenotypes were recorded with saliva-derived isolates of *Actinomyces*, *Rothia*, and *Granulicatella* species. It is important to note that while growth in planktonic phase improved, several species saw reductions in biofilm biomass, potentially from loss of adhesion due to the presence of salivary mucins (27). The role saliva plays during biofilm growth will need to be further explored to fully realize its potential benefits via more advanced methodologies.

**Fig 9:**
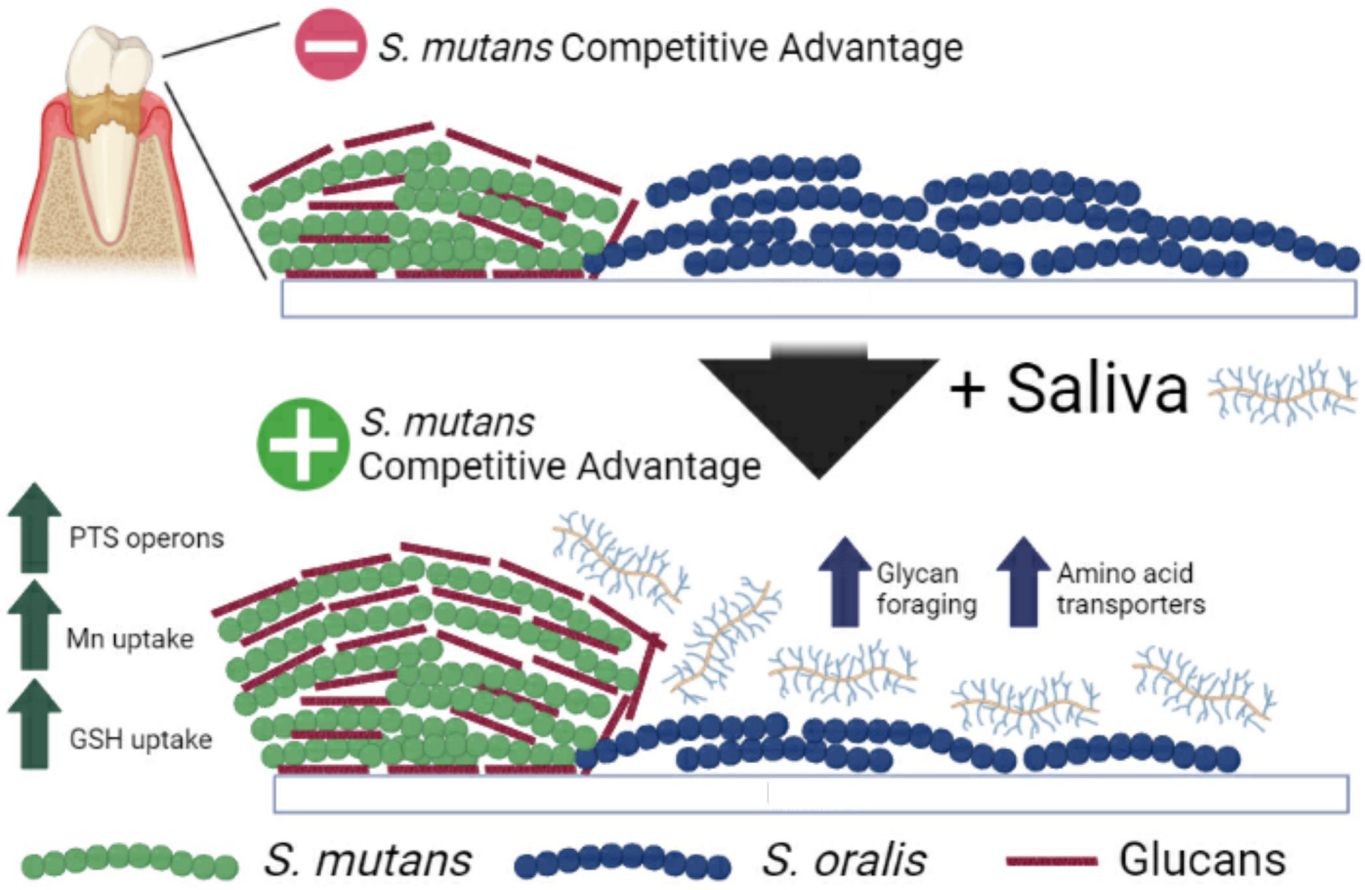
*Saliva modifies the competitive behaviors of oral streptococci*. Two oral streptococci, *S. mutans* (green) and *S. oralis* (blue), colonize supragingival biofilms attached to the tooth’s surface. In cocultures competed in lab-based medium, *S. oralis* maintains a competitive advantage over *S. mutans*. However, when saliva is added to the medium, the competitive behaviors of *S. mutans* are enhanced through upregulation of operons related to carbohydrate uptake and utilization as well as pathways for oxidative stress tolerance. In *S. oralis*, the presence of saliva leads to enhanced expression of genes related to glycan foraging and amino acid transporters. The figure artwork was created using BioRender.

Another surprise occurred during coculture competitions between *S. mutans* and several different commensal species. We initially assumed that since several commensals saw drastic growth improvement in saliva, such as *S. oralis*, commensals would gain a higher competitive edge. However, *S. mutans* was more competitive against four out of the six different commensal species we tested, as well as with *S. sobrinus* that is commonly found together with *S. mutans* in carious lesions (60–62). A limitation of this study was not testing *S. mutans* expansion in mixed-species communities that better represents the composition of supragingival biofilms. Due to significant changes in both doubling times and reductions in lag times, our current working hypothesis is that saliva “jump starts” metabolic pathways in several species, including carbohydrate uptake and glycolytic pathways in *S. mutans* characterized by our transcriptomic data sets (Fig. 9). We were able to capture upregulation of at least three *S*. *mutans* PTS operons in saliva, as well as the trehalose PTS and maltose ABC transporters during coculture with *S. oralis*. We also saw upregulation of *fruAB*, pyruvate formate-lyase (*pfl*), and the glycogen synthesis operon (*glg*), suggesting heterofermentative growth on non-glucose sugars with potential concurrent intracellular polysaccharide (IPS) accumulation. A variety of carbohydrates likely present in saliva is plausibly responsible for these transcriptional changes, even if present in low micromolar amounts. While we did not specifically test for other carbohydrates, we did quantify less than 125 µM glucose present in our saliva preparations, similar to other previously reported values (15, 63). Additionally, we found upregulation of genes responsible for mutanobactin synthesis, a compound described to be important for commensal-produced hydrogen peroxide tolerance (37). Enhanced *S. mutans* competitiveness may also be due, in part, to increased oxidative stress tolerance supplied by this compound. Another intriguing possibility are quorum-sensing molecules such as AI-2 that could be carried in our donor-provided saliva that upregulates metabolism-related genes when present (64, 65). Of note, genes related to genetic competence and bacteriocin production were downregulated, a similar transcriptional profile for *S. mutans* cultured with mucin *O*-linked glycans (66).

Our transcriptional data of *S. mutans* and *S. oralis* cocultures also hints of each species developing nutritional niches under mixed-species growth. The best example of this involves the *lac* operon and tagatose 6-phosphate pathway for the metabolism of galactose. During coculture, *S. mutans* downregulates the genes within this pathway while they are concurrently upregulated in *S. oralis*. In general, there was no significant overlap in the regulation of orthologous genes between strains during growth in saliva suggesting species-specific responses, with the tagatose pathway being one of the few gene sets common between species. *S. mutans* also upregulates pathways for trehalose and maltose uptake, two carbohydrates that do not abundantly support the growth of *S. oralis* (Fig. S8). In turn, we see upregulation of genes in *S. oralis* related to glycan foraging. Rather than competing for the same non-glucose carbohydrates, it is tempting to speculate that these organisms may be utilizing carbon sources the other species do not. To begin to test this hypothesis, we completed a competitive index assay where providing galactose, mannose, trehalose, and cellobiose in addition to glucose favored *S. mutans* over *S. oralis*. Other experiments will be required to further test this hypothesis and potential variability between species. Targeting of transport systems that do not overlap with those found in commensals could provide an attractive strategy toward limiting *S. mutans* emergence. Likewise, the taxonomic diversity and resistance to dysbiosis of saliva-derived oral communities have recently been shown to be increased with mucin glycans (28), highlighting that these nutritional niche partitionings may be key in maintaining microbiome homeostasis by suppressing outgrowth of pathogens.

A final note was the observation of multiple oxidative stress tolerance pathways upregulated in *S. mutans* with saliva, especially under the coculture condition. This included upregulation of manganese transporters *sloABC* and *mntH*, recently characterized to show cooperative activity in manganese-restricted conditions (51, 67), as well as concurrent downregulation of iron transporters. Manganese can assist in oxidative stress tolerance through acting as a cofactor of superoxide dismutase to neutralize hydroxyl radicals (68), while free iron can produce hydroxyl radicals via Fenton chemistry (69). Additionally, manganese may also stimulate carbohydrate metabolism (70, 71). We noted increased *S. mutans* growth in coculture with addition of 1 µM manganese, a physiologically relevant concentration that is present in resting saliva (72). This result not only validates our approach of characterizing intermicrobial interactions leading to strategies of manipulation that can shift the balance between organisms but also highlights the critical role of trace metals in *S. mutans* physiology.

Our study into how saliva has impact on the growth, biofilm formation, and competitive behaviors between commensal and mutans group streptococci has solidified the importance of studying mixed-species interactions in conditions that more closely resemble their *in vivo* condition. While many lingering questions remain, our results provide new insights and findings that offer promising directions for the further evaluation of supporting the persistence of health-associated microbes while developing interventions to combat oral pathogens.

## MATERIALS AND METHODS

### Human saliva and growth media

Commercially available pooled human saliva was purchased from Innovative Research. Upon receipt, the saliva was thawed, centrifuged at 4,500 RPM for 10 minutes, and then passed through a filter unit prior to local frozen storage as 10-mL aliquots. For experimental use, the saliva was thawed and used the same day. Bacterial strains used in this study (Table S7) were cultured in BHI, Tryptone Yeast extract with Glucose (TY-, 10 g tryptone, 5 g yeast extract, 3 g K_2_HPO_4_, and 3.6 g glucose L^−1^) or the chemically defined medium CDM (24–26). For all biofilm-related experiments, 1.7 g L^−1^ sucrose was added. To prepare media using –Water or –Saliva, a percentage (%) of the final volume of either liquid was first added to TY- or CDM- that had been prepared without the addition of carbohydrate. After mixing, carbohydrate(s) were then added to all media for a consistent concentration (20 mM) within all groups. Catalog numbers for all materials used in this publication are listed within Table S8.

### Overnight cultures, strain inoculation, and growth measurements

Overnight cultures were harvested by centrifugation, washed, and normalized to OD_600 nm_ = 0.1 with 1× phosphate-buffered saline (PBS) prior to back dilution (1:100) into the experimental medium of choice. For coculture competitions, strains were inoculated according to Table S9. Growth measurements were completed using a Bioscreen C MBR automated turbidometric analyzer (Growth Curves Ab Ltd., Helsinki, Finland) with the OD_600 nm_ recorded every 0.5 h for 24 h. Wells were overlaid with 0.05 mL sterile mineral oil. Resulting data were used to calculate doubling time [D = (time in minutes; 120)/(*n*, number of generations)] , final yield (average of OD_600 nm_ for last two time points recorded), and time to OD_600 nm_ = 0.1 (half-hour interval when OD_600 nm_ was first >0.1). All experiments were completed with three biological replicates measured in technical triplicates.

### Biofilm microscopy

Bacterial strains were inoculated into medium that contained 1 µM Alexa Fluor 647-labeled dextran, added to glass bottom, black plates, and incubated at 37°C and 5% CO_2_ for 24 h. Resulting biofilms were first washed with 1× PBS and incubated with BSA blocking buffer at room temperature for 0.5 h. Biofilms were then probed with two murine monoclonal antibodies against B-DNA and Z-DNA (2 µg mL^−1^) for 1 h at room temperature. The biofilms were washed and incubated for 1 h at room temperature with an Alexa Fluor 594-labeled goat anti-mouse IgG secondary antibody (2 µg mL^−1^). Finally, the biofilms were washed and stained with Hoechst 33342 solution (5 µM final concentration) for 15 minutes. All biofilms were imaged using a 20× or 40× phase objective on a Agilent Biotek Lionheart FX automated microscope (Agilent Biotek, Winooski, Vermont, United States). Images were captured using Gen5 Image+ software, and quantification of biomass and biofilm thickness were completed either with the Gen5 Image+ software or by importing .TIF files into BiofilmQ (73). Five images of each sample, taken at 2,500-micron increments to avoid observer bias, were acquired.

### Isolation of bacterial strains from human saliva

During preparation of saliva, 0.05 mL of non- or diluted saliva was spread plated on BHI agar. Plates from the thawed-only, non-processed sample contained several different bacterial colony morphologies that were picked randomly (*n* = 50) and grown overnight in BHI medium (37°C and 5% CO_2_). Cultures that grew were then streaked for single-colony isolation again. Plates that were confirmed to contain a single-colony morphology were then selected, stored long term, given a SOSUI (saliva Ohio State University isolate) identifier number, and species identified by Sanger sequencing from Eurofins Genomics using 16S rRNA primers (F - AGA GTT TGA TCC TGG CTC AG; R - TAC GGG TAC CTT GTT ACG ACT) purchased from Integrated DNA Technologies. SOSUI isolate and species identity are listed in Table S10.

### Crystal Violet biofilm biomass quantification

Strains were inoculated into 96-well plates and incubated for 24 h at 37°C and 5% CO_2_. Following, medium from the biofilms was aspirated and plates were dunked into a bucket of water. At room temperature for 15 minutes, 0.05 mL of 0.1% crystal violet was added and incubated. The solution was removed, and plates were dunked again to remove excess crystal violet. Next, 0.2 mL of 30% acetic acid solution was added to extract the bound crystal violet and diluted 1:4 with water before the absorbance at 575 nm was recorded. All biofilm experiments were completed with three biological replicates measured in technical quadruplicates.

### Fluorescent Intensity-based competitive growth assays

*S. mutans* UA159 GFP− (pMZ) or GFP+ strains (pMZ-P*veg::gfp*) were inoculated into their respective growth media along with competitor species. Cultures were plated along with a 0.05-mL sterile mineral oil overlay and incubated for 24 h at 37°C in a Agilent Biotek Synergy H1 multimode plate reader with the OD_600 nm_ and the fluorescent intensity of GFP (excitation 485 nm, emission 528 nm, optics bottom, gain 100) recorded every 0.5 h. For data analysis, the OD_600 nm_ of a medium-only blank was subtracted from respective optical density readings and fluorescent intensity (a.u.) of cultures containing GFP− were subtracted from cultures containing GFP+ (medium/cell background fluorescence). After plotting the resulting data points in GraphPad Prism, an AUC of the fluorescent intensity was calculated using built-in analysis tools. All experiments were completed with three biological replicates measured in technical quadruplicates.

### Colony-forming unit competitive index assays

1 x 10^5^ cells mL^-1^ each of *S. mutans* (kanamycin resistant) and commensal (spectinomycin resistant) were inoculated. Part of the inoculum was serially diluted and plated onto both BHI kanamycin (selection of *S. mutans*) and BHI spectinomycin (selection of commensal) agar plates. Colony forming units (CFUs) were later enumerated from these agar plates after incubation to determine the initial cell count (*t*_i_ = 0 h). The remaining inoculum was incubated at 37°C and 5% CO_2_ for 24 h. Resulting cultures were harvested, washed and resuspended with 1x PBS while transferring to a 5 mL polystyrene round-bottom tube. To isolate single cells, tubes were sonicated within a water bath sonicator (four intervals of 30 s, resting 2 min on ice). Cultures were serially diluted and plated on both BHI kanamycin and spectinomycin agar plates and incubated for 48 h at 37°C and 5% CO2. Following CFU counting of the final cell count (t_f_ = 24 h), a competitive index was calculated using the following formula:


$$CI=Log10\;\;[(t_{f}\;\;S.mutans\;\;CFU/\;t_{f}\;\;commensal\;\;CFU)\;\;/\;(t_{i}\;\;S.mutans\;\;CFU\;/\;t_{i}\;\;commensal\;\;CFU)]$$

### Harvesting cultures and RNA isolation for RNA-Seq

Selected bacterial strains, in either monoculture or cocultures, were grown in the respective media until a measured optical density (OD_600 nm_) of 0.4 was reached before harvesting by centrifugation. RNA isolation was carried out as described elsewhere (14). RNA was sequenced through SeqCenter with their 8 M Single Reads package applied to RNA from monocultures and their 16 M Single Reads package applied to cocultures. Delivered .FASTQ files were uploaded and analyzed through Galaxy (74) with a custom pipeline (14, 75, 76) that included FASTQ Groomer (v 1.1.5), FASTQ Quality Trimmer (v 1.1.5), mapping of reads with Bowtie2 (v 2.3.4.3), and htseq-count (v 0.9.1) on genome features from species-specific .GFF3 files that resulted in a .CSV file containing non-normalized reads counts. The number and percentage of reads mapped to each species are listed within Table S11. All read counts were combined into a single .CSV file and uploaded to Degust (77) and edgeR analysis performed to determine log2 fold change and false discovery rates (FDR) for all genome features. The *P* value was obtained by taking the –Log10 of the FDR. All genome files for this analysis were accessed through NCBI (Table S12).

### Graphing and statistics

Graphing of data were completed with GraphPad Prism (version 9.0). All statistical analysis was completed within GraphPad Prism using built-in analysis tools, including PCA of RNA-Seq data, one-way or two-way ANOVA with post hoc tests (Dunnett’s or Tukey’s test) for a multiple comparison, and AUC calculations.

## Data Availability

The resulting RNA-Seq raw sequencing and data files from this study are available from NCBI-GEO (Gene Expression Omnibus) under accession number GSE239483.
