# Erratum for Choi et al., “Human saliva modifies growth, biofilm architecture, and competitive behaviors of oral streptococci”

**DOI:** 10.1128/msphere.00868-24

**Published:** 2024-11-06

**Authors:** Allen Choi, Kevin Dong, Emily Williams, Lindsey Pia, Jordan Batagower, Paige Bending, Iris Shin, Daniel I. Peters, Justin R. Kaspar

## ERRATUM

Volume 9, no. 2, e00771-23, 2024, https://doi.org/10.1128/msphere.00771-23. Due to a processing error, the supplemental material files are missing. The supplemental material is given in this erratum.

## SUPPLEMENTAL MATERIAL

Supplemental figures and tables msphere.00868-24-s0001.pdf Figures S1 to S8 and Tables S1 to S12.

RNA-Seq data set msphere.00868-24-s0002.xlsx Complete RNA-Seq data output for each gene within each species (listing only significant genes).

ASM does not own the copyrights to Supplemental Material that may be linked to, or accessed through, an article. The authors have granted ASM a non-exclusive, world-wide license to publish the Supplemental Material files. Please contact the corresponding author directly for reuse.

