# Supplementary material for: Erratum for Choi et al., “Human saliva modifies growth, biofilm architecture, and competitive behaviors of oral streptococci”
Source: mSphere. 2024 Nov 6;9(12):e00868-24. doi: 10.1128/msphere.00868-24 (PMC11656814; doi:10.1128/msphere.00868-24)
Supplement: Supplemental figures and tables — Figures S1 to S8 and Tables S1 to S12. [file msphere.00868-24-s0001.pdf]

Supplemental Material

Human Saliva Modifies Growth, Biofilm Architecture and Competitive Behaviors of Oral Streptococci

Allen Choi*, Kevin Dong*, Emily Williams*, Lindsey Pia, Jordan Batagower, Paige Bending, Iris Shin, Daniel I. Peters, and Justin R. Kaspar #

Division of Biosciences, The Ohio State University College of Dentistry, Columbus, Ohio

Table of Contents:

Supplemental Figures and Figure Legends (1-8): Pages 2 - 9

Supplemental Tables (1-12): Pages 10 – 37

Supplemental Excel File: Contains analyzed RNA-Seq data (log2 fold change and –log10 P values) for each gene within either indicated species, used to construct Supplemental Tables 1, 2, 3 and 5.

* Co-first authors
# Corresponding author

Mailing address:
Division of Biosciences, The Ohio State University, College of Dentistry,
305 W. 12th Avenue, Postle Hall Rm 4185, Columbus, OH 43210.
Phone: (614) 292-3373
E-mail: kaspar.17@osu.edu

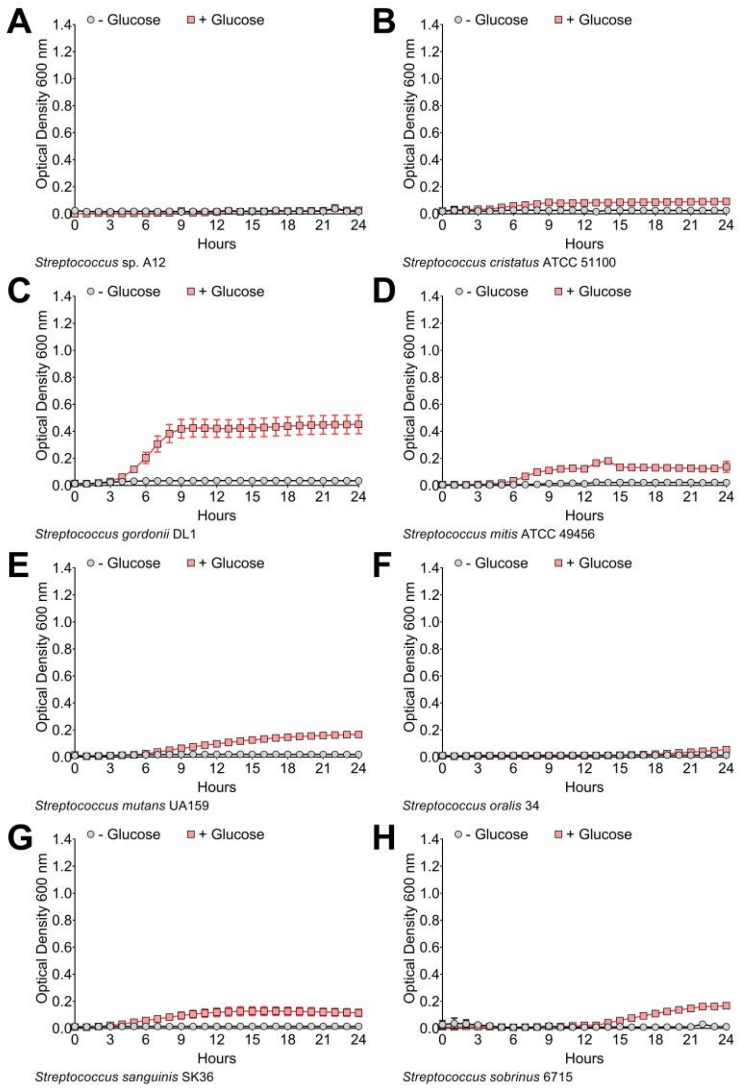

Supplemental Figure 1. Growth of oral streptococci in pure human saliva. Growth curves of oral streptococci species **(A)** *S*. sp. A12, **(B)** *S. cristatus* ATCC 51100, **(C)** *S. gordonii* DL1, **(D)** *S. mitis* ATCC 49456, **(E)** *S. mutans* UA159, **(F)** *S. oralis* 34, **(G)** *S. sanguinis* SK36 and **(H)** *S. sobrinus* 6715 in either 100% human saliva with no addition of carbohydrate (- Glucose; grey circles) or supplemented with 20 mM glucose (+ Glucose; red squares). Data points for optical density at 600 nm recorded every hour over a 24 h period are shown. All growth curves were completed with three biological replicates measured in technical triplicates.

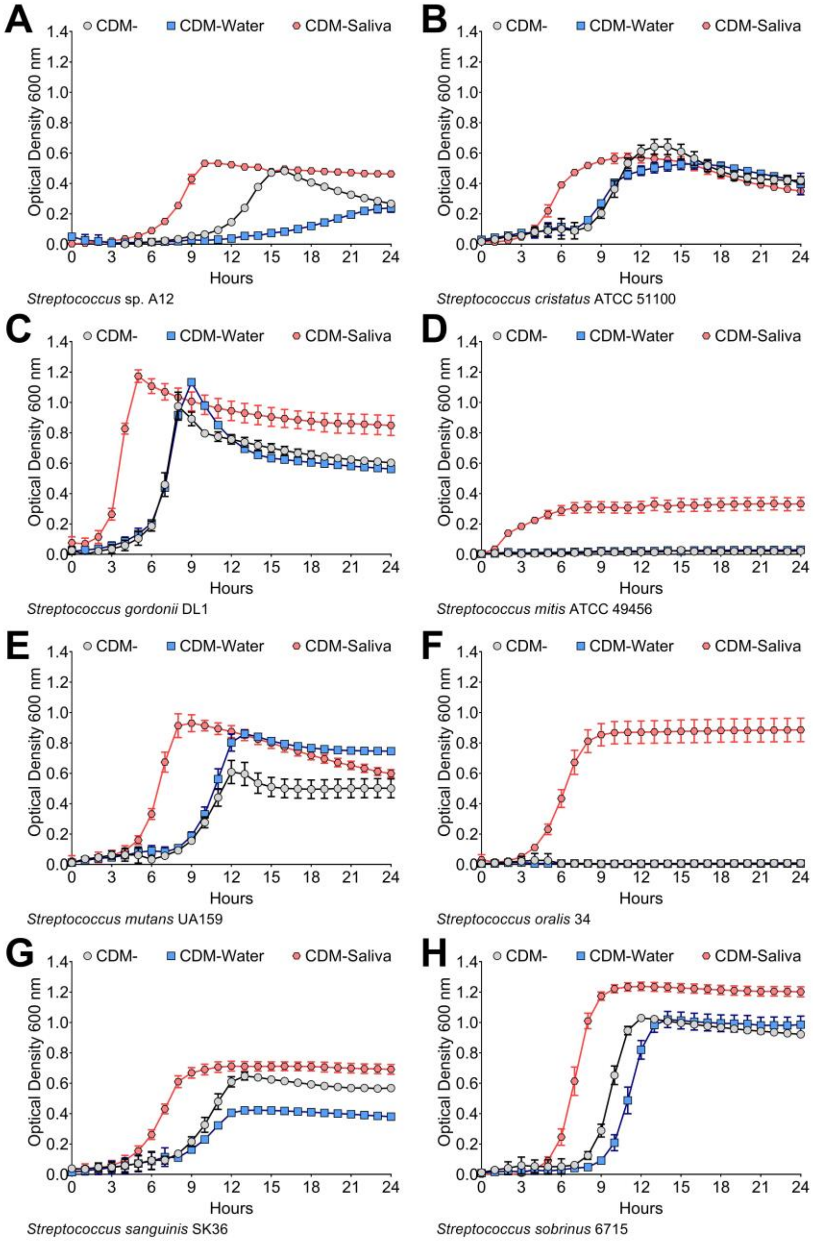

Supplemental Figure 2. Growth of oral streptococci in human saliva with CDM medium. Growth curves of oral streptococci species **(A)** *S*. sp. A12, **(B)** *S. cristatus* ATCC 51100, **(C)** *S. gordonii* DL1, **(D)** *S. mitis* ATCC 49456, **(E)** *S. mutans* UA159, **(F)** *S. oralis* 34, **(G)** *S. sanguinis* SK36 and **(H)** *S. sobrinus* 6715 in either 100% CDM (CDM-; grey circles), 50% CDM / 50% H_2O (TY-Water; blue squares), or 50% CDM / 50% human saliva (CDM-Saliva; red hexagons). Data points for optical density at 600 nm recorded every hour over a 24 h period are shown. All growth curves were completed with three biological replicates measured in technical triplicates.

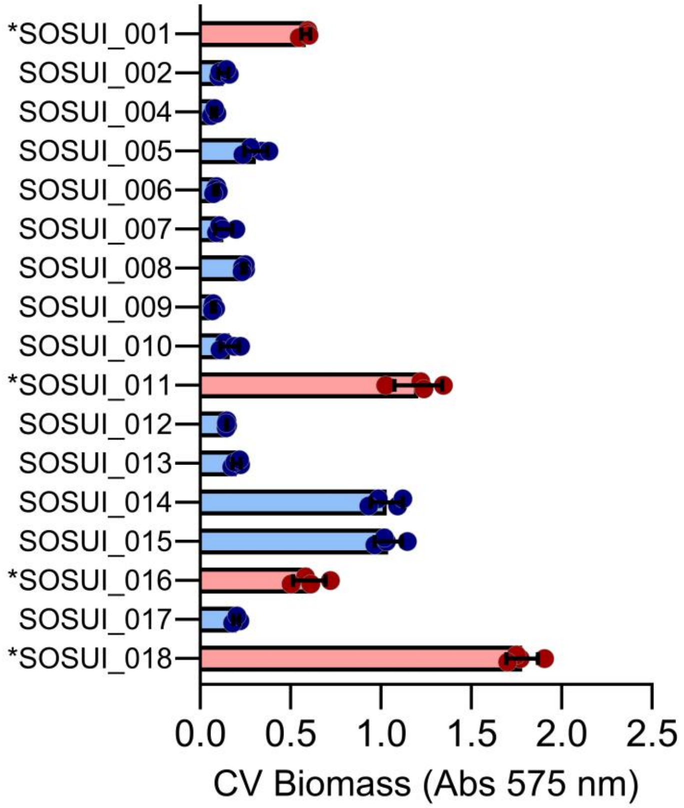

Supplemental Figure 3. Accumulated biomass of saliva-derived bacterial isolates. Crystal violet biomass of 17 different saliva-derived bacterial isolates, grown in TY- medium for 24 h. Data was quantified by extracting crystal violet with 30% acetic acid and measuring the absorbance at 575 nm within a Agilent Biotek® Synergy™ H1 multimode plate reader. Red bars and data points, along with asterisk next to SOSUI identifier number, denote isolates that were carried forward for further characterization. n = 4.

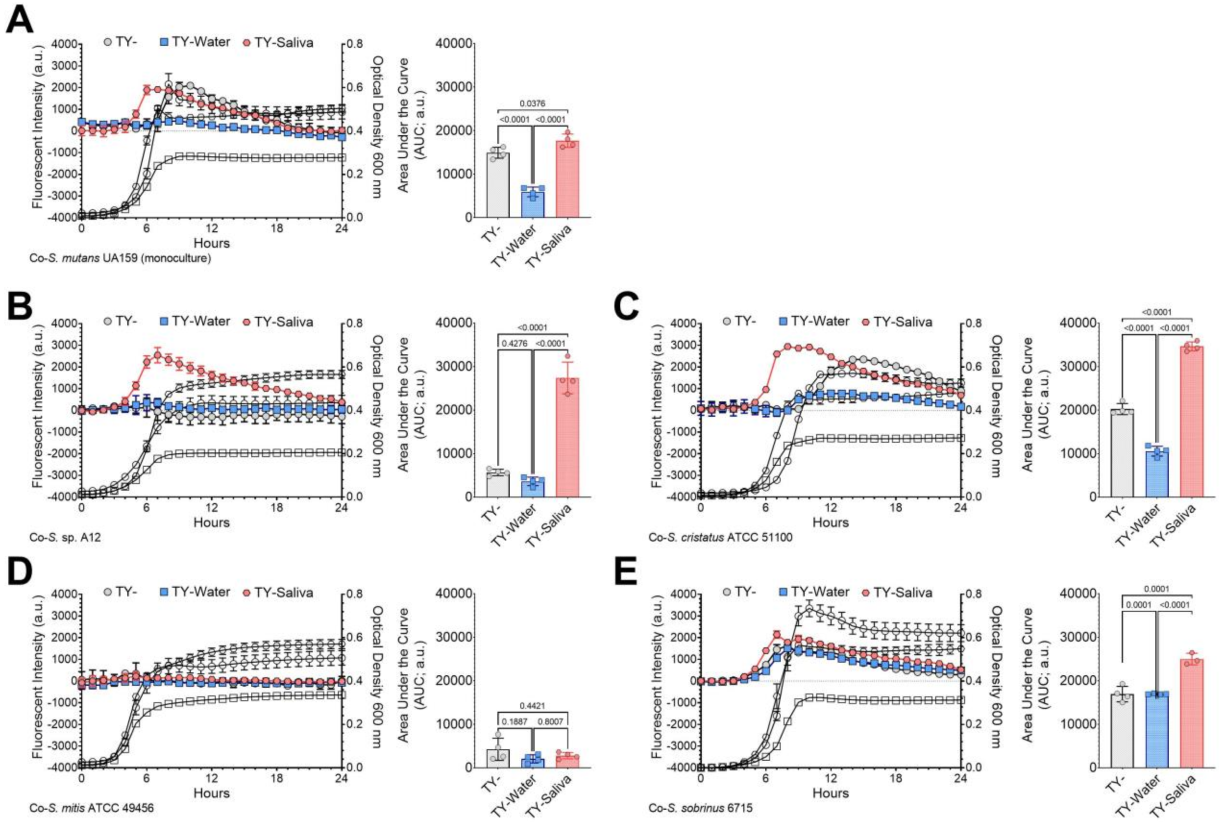

Supplemental Figure 4. Coculture competition between *S. mutans* and oral streptococci competitors in human saliva with TY medium. Fluorescence-based (intensity, arbitrary units, a.u., left y-axis) growth profile of *S. mutans* in coculture competition against **(A)** *S. mutans* UA159 (monoculture growth control), **(B)** *S.* sp. A12, **(C)** *S. cristatus* ATCC 51100, **(D)** *S. mitis* ATCC 49456 and **(E)** *S. sobrinus* 6715 in either 100% TY (TY-; grey circles), 50% TY / 50% H_2O (TY-Water; blue squares), or 50% TY / 50% human saliva (TY-Saliva; red hexagons). Growth of the entire coculture, measured by optical density at 600 nm, is shown in black outline using the same symbol (right y-axis). The area under the curve (AUC) of the fluorescent intensity of each condition (sum of peaks $\geq$ 0) was quantified and is shown in the graft on the right (n = 4). Data graphing and one-way analysis of variance with multiple comparisons was completed in GraphPad Prism software. All coculture competitions were completed with four biological replicates measured in technical triplicates.

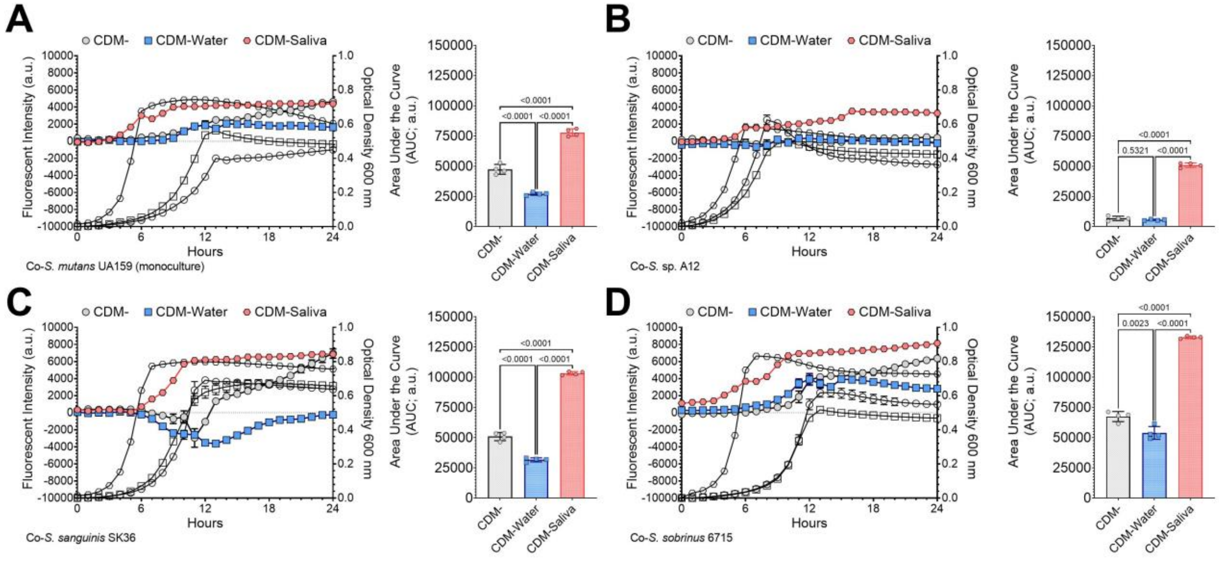

Supplemental Figure 5. Coculture competition between *S. mutans* and oral streptococci competitors in human saliva with CDM medium. Fluorescence-based (intensity, arbitrary units, a.u., left y-axis) growth profile of *S. mutans* in coculture competition against **(A)** *S. mutans* UA159 (monoculture growth control), **(B)** *S.* sp. A12, **(C)** *S. sanguinis* SK36 and **(D)** *S. sobrinus* 6715 in either 100% CDM (CDM-; grey circles), 50% CDM / 50% H_2O (CDM-Water; blue squares), or 50% CDM / 50% human saliva (CDM-Saliva; red hexagons). Growth of the entire coculture, measured by optical density at 600 nm, is shown in black outline using the same symbol (right y-axis). The area under the curve (AUC) of the fluorescent intensity of each condition (sum of peaks $\geq$ 0) was quantified and is shown in the graft on the right (n = 4). Data graphing and one-way analysis of variance with multiple comparisons was completed in GraphPad Prism software. All coculture competitions were completed with four biological replicates measured in technical triplicates.

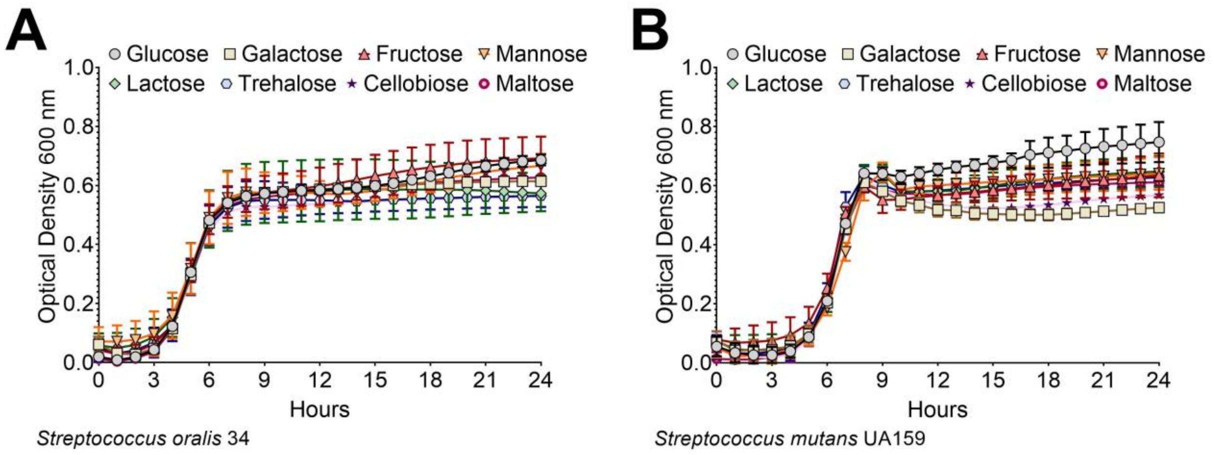

Supplemental Figure 6. Minimum saliva concentration influencing oral streptococci growth. Growth curves of oral bacterial species **(A)** *Streptococcus oralis* 34 and **(B)** *Streptococcus mutans* UA159 in TY-Saliva with 10 mM glucose and 10 (monosaccharides) or 5 (disaccharides) additional carbohydrate as indicated: glucose (light grey circles), galactose (yellow squares), fructose (red triangles), mannose (orange upside-down triangles), lactose (green diamond), trehalose (blue hexagon), cellobiose (purple stars) or maltose (pink cirlces). Data points for optical density at 600 nm recorded every hour over a 24 h period are shown. All growth curves were completed with three biological replicates measured in technical triplicates.

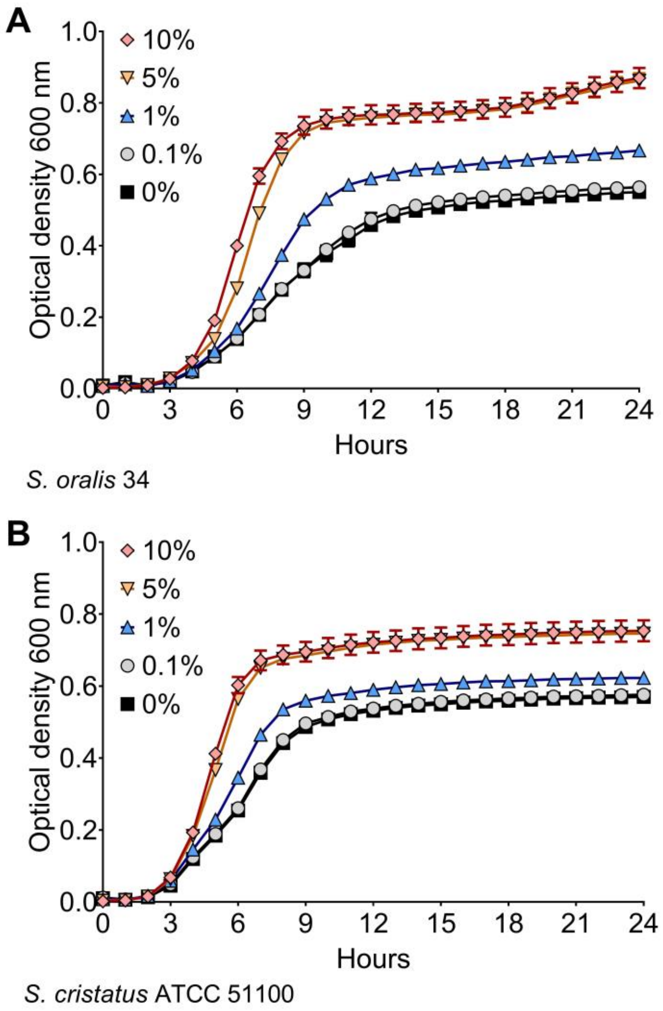

Supplemental Figure 7. Minimum saliva concentration influencing oral streptococci growth. Growth curves of oral bacterial species **(A)** *Streptococcus oralis* 34 and **(B)** *Streptococcus cristatus* ATCC 51100 in TY with either 0% (black squares), 0.1% (light grey circles), 1% (blue upright triangles), 5% (orange downward triangles) or 10% saliva (red diamonds). Data points for optical density at 600 nm recorded every hour over a 24 h period are shown. All growth curves were completed with three biological replicates measured in technical triplicates.

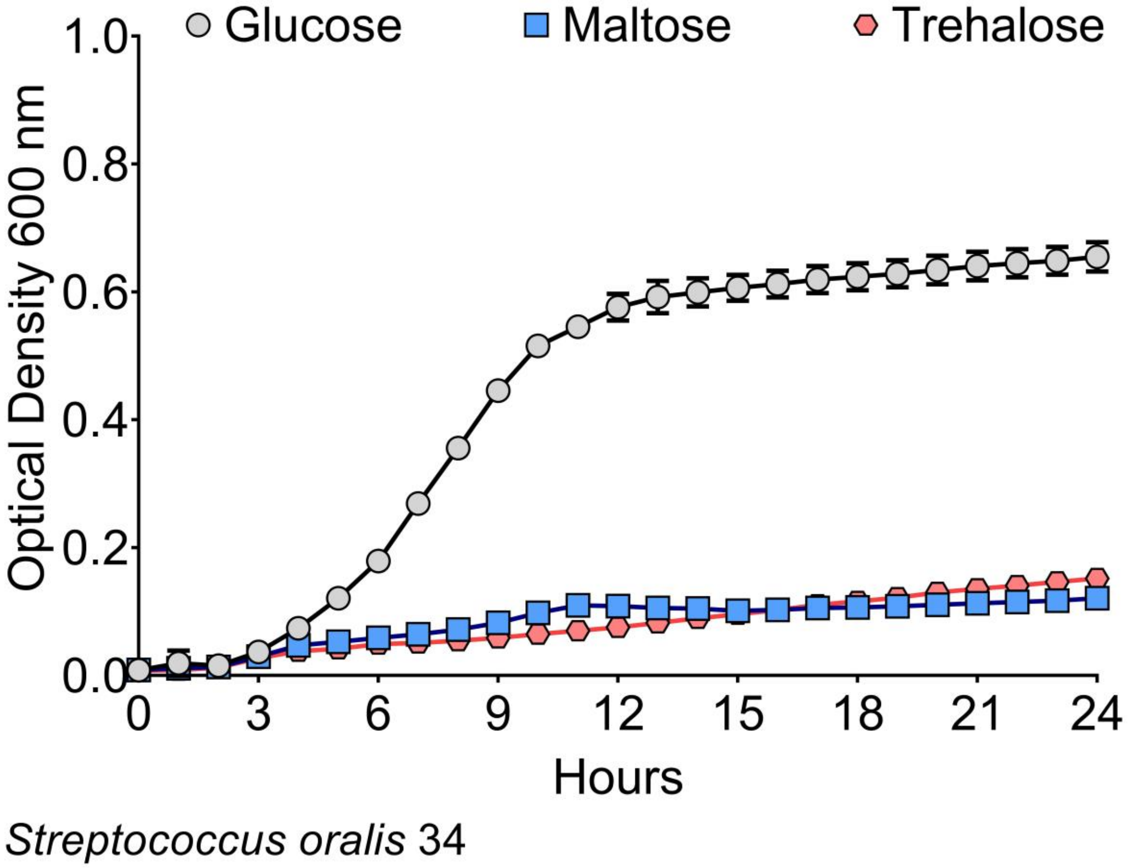

Supplemental Figure 8. *S. oralis* growth on maltose and trehalose. Growth curve of *Streptococcus oralis* 34 in TY- with 20 mM glucose (grey circles), 10 mM maltose (blue squares), or 10 mM trehalose (red hexagons). 10 mM was used for maltose and trehalose as both are disaccharides compared to the monosaccharide glucose. Data points for optical density at 600 nm recorded every hour over a 24 h period are shown. All growth curves were completed with three biological replicates measured in technical triplicates.

SUPPLEMENTAL TABLES

Supplemental Table 1. Differentially expressed genes* (DEGs) between *Streptococcus mutans* UA159 grown in TY- vs TY-Saliva**.** Red, upregulated genes. Blue, downregulated genes. *Differentially expressed genes = genes with ≥4 Log10 *P* value and Log2 fold change (FC) ≥ (-)1.

Gene ID	Gene	Product	Log2 Fold Change	Fold Change	P value	Log10 P value
**SMU_1960c**		fructose-specific Enzyme IIB component	3.125208401	8.725322163	2.46E-32	31.61
**SMU_1957**		fructose-specific Enzyme IID component	3.114004866	8.657826357	1.24E-29	28.91
**SMU_1958c**		fructose-specific Enzyme IIC component	3.097439116	8.55898142	6.33E-20	19.20
**SMU_1961c**		fructose-specific Enzyme IIA component	2.904885035	7.489581127	1.92E-16	15.72
**SMU_1956c**		conserved hypothetical protein	2.716391007	6.572266617	1.04E-24	23.98
**SMU_438c**		(R)-2-hydroxyglutaryl-CoA dehydratase activator-related protein	2.716257527	6.571658571	1.98E-41	40.70
**SMU_1600**	*ptcB*	PTS system IIB component, required for cellobiose uptake and metabolism	2.598851835	6.058043069	5.72E-12	11.24
**SMU_1599**	*celR*	transcriptional regulator, required for cellobiose uptake and metabolism	2.364885953	5.151119299	9.36E-25	24.03
**SMU_1346**	*bacT*	thioesterase II-like protein	2.152938261	4.447326305	1.31E-08	7.88
**SMU_1538**	*glgC*	glucose-1-phosphate adenylyltransferase	2.064996569	4.184329809	1.16E-21	20.93
**SMU_1419**		transcriptional regulator	2.056051256	4.15846548	6.61E-26	25.18
**SMU_1345c**		peptide synthetase similar to mycA	2.05489944	4.155146778	2.29E-10	9.64
**SMU_1537**	*glgD*	glycogen biosynthesis protein	1.94734188	3.856633042	2.16E-30	29.67
**SMU_1420**		NADPH-quinone reductase	1.918076089	3.779187477	7.65E-19	18.12
**SMU_1539**	*glgB*	1,4-alpha-glucan branching enzyme	1.914043226	3.768638009	9.91E-21	20.00
**SMU_104**		glycosyl hydrolase, alpha-glucosidase	1.890923313	3.708725043	2.46E-21	20.61
**SMU_102**		PTS system, IID component	1.88495483	3.693413601	8.00E-16	15.10
**SMU_103**		PTS system, IIA component	1.855105274	3.617781491	1.75E-16	15.76
**SMU_1343c**		polyketide synthase	1.811891503	3.511023137	4.55E-10	9.34
**SMU_1536**	*glgA*	glycogen synthase	1.804805862	3.493821401	1.68E-14	13.77
**SMU_2127**		succinic semialdehyde dehydrogenase (NAD-dependent aldehyde dehydrogenase)	1.784204324	3.444284521	3.30E-16	15.48
**SMU_1597c**		conserved hypothetical protein	1.778894889	3.431632092	2.21984E-05	4.65
**SMU_1341c**		gramicidin S synthase/mycosubtilin synthetase chain mycB	1.71400026	3.280692236	7.24E-08	7.14
**SMU_1344c**		malonyl CoA-acyl carrier protein transacylase	1.699962219	3.248924503	3.03E-07	6.52
**SMU_1596**	*ptcC*	PTS system, cellobiose-specific IIC component	1.698527876	3.245695991	1.11E-14	13.96
**SMU_1340**	*bacA2*	bacitracin synthetase 1/ tyrocidin synthetase III	1.690822557	3.228407199	3.45E-08	7.46
**SMU_105**		SCR operon transcriptional repressor	1.683203679	3.211402907	1.30E-17	16.88
**SMU_941c**		conserved hypothetical protein	1.627849022	3.09051876	5.05E-12	11.30
**SMU_1347c**			1.621577935	3.077114089	9.95E-08	7.00
**SMU_1006**		ABC transporter, ATP-binding protein	1.614283559	3.06159523	1.24E-14	13.91
**SMU_940c**		hemolysin III-related protein	1.594939157	3.020817772	1.20E-16	15.92
**SMU_1047c**		hypothetical protein	1.593608046	3.018031882	9.44E-08	7.02
**SMU_1342**	*bacA1*	bacitracin synthetase	1.575621156	2.980637971	5.25E-07	6.28
**SMU_1339**	*bacD*	bacitracin synthetase; surfactin synthetase	1.559725943	2.947978377	2.00E-07	6.70
**SMU_1365c**			1.5352446	2.89837567	2.96E-07	6.53
**SMU_1535**	*phsG*	glycogen phosphorylase	1.522063198	2.872014828	3.73E-14	13.43
**SMU_1336**	*pksD*	conserved hypothetical protein	1.491326654	2.8114739	2.04946E-05	4.69
**SMU_180**		oxidoreductase, possible fumarate reductase	1.465122047	2.760868258	3.27E-12	11.49
**SMU_980**	*bglP*	beta-glucoside-specific EII permease	1.462866312	2.756554857	3.12E-16	15.51
**SMU_1895c**		hypothetical protein	1.455773058	2.743035053	1.08185E-06	5.97
**SMU_1004**	*gtfB*	glucosyltransferase-I	1.450685607	2.733379179	2.85E-11	10.54
**SMU_1337c**		alpha/beta superfamily hydrolases	1.435906505	2.705521123	9.15E-07	6.04

**SMU_402**	*pfl*	pyruvate formate-lyase	1.396097646	2.631887179	1.18E-09	8.93
**SMU_78**	*fruA*	fructan hydrolase; exo-beta-D-fructosidase	1.359901719	2.566676939	7.08E-07	6.15
**SMU_1348c**			1.359462823	2.565896225	5.00E-08	7.30
**SMU_1366c**			1.349450294	2.548150155	7.15E-08	7.15
**SMU_1007**		ABC transporter permease	1.328916533	2.512139419	6.88E-12	11.16
**SMU_1005**	*gtfC*	glucosyltransferase-SI	1.315997534	2.489744215	5.98E-13	12.22
**SMU_1335c**		enoyl-acyl carrier protein(ACP) reductase	1.296040625	2.455540512	1.36E-08	7.87
**SMU_1077**	*pgm*	phosphoglucomutase	1.263563113	2.400879694	1.97E-11	10.71
**SMU_1425**	*clpB*	ATP-dependent Clp protease, ATP-binding subunit ClpB	1.257133111	2.390202933	1.23E-08	7.91
**SMU_1879**		mannose PTS system component IID	1.239548802	2.361246735	1.85E-10	9.73
**SMU_981**	*bglB1*	beta-glucosidase, BglB protein	1.230064405	2.345774616	9.14644E-06	5.04
**SMU_1421**	*pdhC*	dihydrolipoamide acetyltransferase (acetoin dehydrogenase E2 component)	1.213704933	2.3193249	5.69E-07	6.24
**SMU_101**		sorbose PTS system, IIC component	1.198424043	2.294888473	3.66277E-06	5.44
**SMU_79**	*fruB*	fructan hydrolase; exo-beta-D-fructosidase	1.184050164	2.272137533	1.22367E-05	4.91
**SMU_1338c**		ABC transport macrolide permease	1.165052848	2.242414297	1.23E-07	6.91
**SMU_1862**		hypothetical protein	1.091136405	2.130417825	0.000100124	4.00
**SMU_1601**	*bgl*	6-phospho-beta-glucosidase, required for cellobiose uptake and metabolism	1.038815524	2.05454015	1.19734E-05	4.92
**SMU_197c**		hypothetical protein	-1.000744983	-2.001033033	2.62472E-05	4.58
**SMU_1750c**		hypothetical protein	-1.001381568	-2.001916177	5.34218E-06	5.27
**SMU_673**		conserved hypothetical protein	-1.009451817	-2.013146017	1.39E-09	8.86
**SMU_837**		oxidoreductase, aldo/keto reductase family	-1.012302782	-2.017128205	1.96E-07	6.71
**SMU_80**	*hrcA*	heat-inducible transcription repressor	-1.031585549	-2.044269708	3.23735E-06	5.49
**SMU_1752c**		hypothetical protein	-1.035292613	-2.049529297	4.34E-07	6.36
**SMU_860**	*pyrAB*	carbamoyl-phosphate synthase, large subunit	-1.045932413	-2.064700342	1.99877E-06	5.70
**SMU_997**		inorganic ion ABC transporter,ATP-binding protein; possible ferrichrome transport system	-1.048352657	-2.06816696	3.53E-09	8.45
**SMU_198c**		conjugative transposon protein	-1.066080334	-2.093737146	2.00375E-05	4.70
**SMU_856**	*pyrR*	bifunctional protein: pyrimidine operon regulatory protein and uracil phosphoribosyltransferase	-1.091559327	-2.131042443	7.05E-07	6.15
**SMU_625**	*comEA*	competence protein	-1.09449196	-2.135378718	6.61E-08	7.18
**SMU_764**	*ahpC*	alkyl hydroperoxide reductase, subunit C	-1.103296527	-2.148450483	7.43E-11	10.13
**SMU_1987**	*comYA*	late competence protein; type II secretion system protein E	-1.110860857	-2.15974481	1.98617E-05	4.70
**SMU_644**		competence protein CoiA	-1.114398035	-2.165046536	1.33378E-06	5.87
**SMU_82**	*dnaK*	chaperone protein, DnaK	-1.146340938	-2.213517743	3.89139E-06	5.41
**SMU_765**		alkyl hydroperoxide reductase, subunit F	-1.159612614	-2.233974339	3.89E-13	12.41
**SMU_859**	*pyrA*	carbamoyl-phosphate synthase,small subunit	-1.164560829	-2.241649671	9.44E-08	7.02
**SMU_1980c**		conserved hypothetical protein	-1.172035087	-2.253293257	4.57148E-06	5.34
**SMU_998**		ABC transporter, ferrichrome-binding protein	-1.175694746	-2.259016402	5.64E-12	11.25
**SMU_671**	*citZ*	citrate synthase	-1.225160935	-2.337815269	2.35E-15	14.63
**SMU_81**	*grpE*	co-chaperone protein GrpE	-1.234718699	-2.353354566	3.70462E-06	5.43
**SMU_626**		competence protein; possible integral membrane protein	-1.239662154	-2.361432265	6.50E-09	8.19
**SMU_836**		hypothetical protein	-1.242616169	-2.36627241	5.42E-13	12.27
**SMU_858**	*pyrB*	aspartate transcarbamoylase	-1.249030145	-2.376815873	2.32E-10	9.63
**SMU_609**		cell wall protein precursor	-1.264772117	-2.402892516	2.11E-14	13.68
**SMU_1984**	*comYC*	competence protein ComYC	-1.265428808	-2.403986523	3.16E-07	6.50
**SMU_838**	*gshR*	glutathione reductase	-1.266562711	-2.405876707	6.43E-10	9.19
**SMU_2042**	*dexA*	dextranase (1,6-alpha-glucanhydrolase)	-1.286160206	-2.438780991	3.42E-16	15.47
**SMU_1967**	*ssb2*	single-stranded DNA-binding protein	-1.288702902	-2.44308304	4.18E-13	12.38
**SMU_672**	*idh*	isocitrate dehydrogenase	-1.298853181	-2.460332296	2.21E-15	14.66
**SMU_1983**	*comYD*	competence protein ComYD	-1.318385177	-2.493868124	3.032E-05	4.52
**SMU_499**		late competence protein required for DNA uptake	-1.338875903	-2.52954149	6.60E-07	6.18
**SMU_670**	*citB*	aconitate hydratase; aconitase A	-1.3838615	-2.609659353	1.10E-16	15.96
**SMU_1757c**		conserved hypothetical protein	-1.384352162	-2.610547052	9.65E-10	9.02

**SMU_1224**	*pyrK*	dihydroorotate dehydrogenase electron transfer subunit	-1.386292679	-2.614060768	2.62E-10	9.58
**SMU_1657c**		nitrogen regulatory protein PII	-1.391746681	-2.623961734	1.84628E-06	5.73
**SMU_857**		xanthine/uracil permease	-1.412326465	-2.661660316	2.35E-09	8.63
**SMU_1700c**		LrgB-like protein; possible murein hydrolase regulator	-1.41331532	-2.663485302	5.77E-10	9.24
**SMU_145**		major facilitator superfamily transporter, efflux protein	-1.435379298	-2.70453262	6.68E-12	11.17
**SMU_933**		amino acid ABC transporter, amino acid substrate-binding protein	-1.442062247	-2.7170898	3.91292E-06	5.41
**SMU_1400c**		conserved hypothetical protein	-1.447811055	-2.727938377	5.80E-08	7.24
**SMU_1223**	*pyrDB*	dihydroorotate dehydrogenase	-1.510269236	-2.848631953	6.71E-09	8.17
**SMU_932**		conserved hypothetical protein	-1.5602527	-2.949054941	1.54E-08	7.81
**SMU_936**		amino acid ABC transporter, ATP-binding protein	-1.568137384	-2.965216376	1.08E-10	9.97
**SMU_1755c**		conserved hypothetical protein	-1.591071018	-3.012729234	3.62E-14	13.44
**SMU_1762c**		conserved hypothetical protein	-1.596855965	-3.024833987	6.84E-12	11.16
**SMU_1764c**		conserved hypothetical protein	-1.621327305	-3.076579568	4.74E-17	16.32
**SMU_1758c**		conserved hypothetical protein	-1.643948379	-3.125199695	1.98E-20	19.70
**SMU_934**		amino acid ABC transporter, permease protein	-1.648728347	-3.135571347	2.58E-09	8.59
**SMU_1760c**		conserved hypothetical protein	-1.657225308	-3.154093238	7.84E-23	22.11
**SMU_1761c**		conserved hypothetical protein	-1.676345811	-3.196173691	5.66E-18	17.25
**SMU_1753c**		conserved hypothetical protein	-1.680234525	-3.204800442	2.06E-16	15.69
**SMU_1763c**		conserved hypothetical protein	-1.693336797	-3.234038372	2.08E-18	17.68
**SMU_935**		amino acid ABC transporter, permease protein	-1.711596907	-3.275231554	5.14E-11	10.29
**SMU_2027**		transcriptional regulator/repressor	-1.722083746	-3.299125697	6.41E-13	12.19
**SMU_199c**		hypothetical protein	-1.773424077	-3.418643719	4.45E-08	7.35
**SMU_1001**	*smf*	DNA processing protein, Smf family	-1.815238068	-3.519176976	9.65E-15	14.02
**SMU_1658**	*nrgA*	ammonium transporter, NrgA protein	-1.823057735	-3.538303337	5.76E-13	12.24
**SMU_1175**		sodium:alanine (or glycine) symporter	-1.874096916	-3.665720822	8.94E-25	24.05
**SMU_498**	*comF*	late competence protein F	-1.922658396	-3.791210064	2.27E-17	16.64
**SMU_201c**		conserved hypothetical protein	-1.923670905	-3.793871738	3.59E-13	12.44
**SMU_1754c**		conserved hypothetical protein	-1.935771787	-3.825827395	2.74E-14	13.56
**SMU_210c**		hypothetical protein	-1.938198037	-3.832266887	2.29E-09	8.64
**SMU_202c**		conserved hypothetical protein/Streptococcus-specific protein	-1.950563547	-3.86525487	3.98E-12	11.40
**SMU_205c**		conserved hypothetical protein	-2.195354674	-4.580022462	1.08E-07	6.97
**SMU_208c**		conserved hypothetical protein, FtsK/SpoIIIE family	-2.198727395	-4.590742128	7.02E-21	20.15
**SMU_211c**		hypothetical protein	-2.229807246	-4.690713044	1.84E-11	10.73
**SMU_207c**		transcriptional regulator	-2.247704899	-4.74926711	4.50E-19	18.35
**SMU_214c**		hypothetical protein	-2.27237224	-4.831168729	1.43139E-05	4.84
**SMU_213c**		hypothetical protein	-2.363020549	-5.14446321	2.57E-07	6.59
**SMU_209c**		hypothetical protein	-2.739822831	-6.679882985	1.09E-17	16.96
**SMU_212c**		hypothetical protein	-3.168050845	-8.988315997	1.92138E-05	4.72
**SMU_2037**	*treA*	trehalose-6-phosphate hydrolase	-4.353324156	-20.44001226	1.73E-70	69.76
**SMU_2038**	*pttB*	phosphotransferase system, trehalose-specific IIBC component (EIIBC-tre)	-4.462238739	-22.04284811	2.02E-71	70.69

Supplemental Table 2. DEGs between *Streptococcus oralis* 34 grown in TY- vs TY-Saliva**.** Red, upregulated genes. Blue, downregulated genes.

Gene ID	Gene Name	Gene Description	Log2 Fold Change	Fold Change	P value	Log10 P value
KX728_RS08895		ABC transporter, permease protein 2 (cluster 1, maltose/g3p/polyamine/iron)	8.256835896	305.8829528	1.91E-128	127.72
KX728_RS08900		ABC transporter, permease protein 1 (cluster 1, maltose/g3p/polyamine/iron)	7.533501698	185.2720826	2.19E-142	141.66
KX728_RS08905		Glycosyl hydrolase-related protein	7.486551369	179.3397361	1.94E-156	155.71
KX728_RS08890		ABC transporter, substrate-binding protein (cluster 1, maltose/g3p/polyamine/iron)	5.219436038	37.25690786	2.12E-57	56.67
KX728_RS08885		Bactriocin immunity protein BlpY	4.969272165	31.32564186	3.59E-45	44.45
KX728_RS00335		Glyoxalase family protein	4.778266191	27.44109586	4.40E-57	56.36
KX728_RS06730		Efflux ABC transporter, permease protein	4.728386962	26.50857037	3.45E-14	13.46
KX728_RS02155		Transcriptional regulator, MarR family	4.374242951	20.73854754	4.15E-78	77.38
KX728_RS00215		Phosphoribosylaminoimidazole-succinocarboxamide synthase (EC 6.3.2.6)	4.258012065	19.13327675	7.69E-07	6.11
KX728_RS07065		MBL-fold metallo-hydrolase superfamily	3.897029597	14.89782274	3.31E-39	38.48
KX728_RS02160		Glycine betaine ABC transport system, ATP-binding protein OpuAA (EC 3.6.3.32)	3.646732158	12.52494322	2.82E-51	50.55
KX728_RS02165		Glycine betaine ABC transport system, permease protein OpuAB	3.568837583	11.86662347	5.16E-42	41.29
KX728_RS02500		Transcriptional regulator, MarR family	3.120553247	8.697213482	1.61E-44	43.79
KX728_RS05075	sipA	Signal peptidase I (EC 3.4.21.89)	2.981542922	7.898304134	1.25E-32	31.90
KX728_RS04325		Hydrolase, HAD superfamily	2.932984092	7.636883904	6.57E-31	30.18
KX728_RS05085	pepT	Tripeptide aminopeptidase (EC 3.4.11.4)	2.752447417	6.738593116	2.79E-39	38.55
KX728_RS02495		hypothetical protein	2.636093177	6.216459632	2.00E-35	34.70
KX728_RS06860	tpiA	Triosephosphate isomerase (EC 5.3.1.1)	2.617543968	6.137044173	7.61E-39	38.12
KX728_RS08205	glmS	Glutamine--fructose-6-phosphate aminotransferase [isomerizing] (EC 2.6.1.16)	2.502312465	5.665928771	1.67E-15	14.78
KX728_RS08940		Zinc ABC transporter, substrate-binding lipoprotein AdcA	2.482812698	5.589862122	2.99E-28	27.52
KX728_RS06750		Fructose-bisphosphate aldolase class II (EC 4.1.2.13)	2.479640177	5.577583386	3.22E-19	18.49
KX728_RS05065	srtB	NPQTN specific sortase B	2.434867176	5.407145492	1.02E-33	32.99
KX728_RS00250	purD	Phosphoribosylamine--glycine ligase (EC 6.3.4.13)	2.426052373	5.374208795	1.68E-07	6.78
KX728_RS05070		EftLSL.A	2.363064315	5.144619275	1.23E-30	29.91
KX728_RS05080	pitA	hypothetical protein	2.344276732	5.078057503	1.29E-34	33.89
KX728_RS00225	purF	Amidophosphoribosyltransferase (EC 2.4.2.14)	2.334835134	5.044933085	1.26E-08	7.90
KX728_RS03030		Transcriptional regulator, MarR family	2.323277691	5.004679533	1.73E-32	31.76
KX728_RS00230	purM	Phosphoribosylformylglycinamidine cyclo-ligase (EC 6.3.3.1)	2.299369284	4.922425203	8.88E-08	7.05
KX728_RS03085		Glycosyltransferase	2.289775565	4.889800361	8.06E-08	7.09
KX728_RS04430		Formate--tetrahydrofolate ligase (EC 6.3.4.3)	2.263561788	4.801754979	1.03E-11	10.99
KX728_RS06350		hypothetical protein	2.205537237	4.612462675	1.03E-12	11.99
KX728_RS00235	purN	Phosphoribosylglycinamide formyltransferase (EC 2.1.2.2)	2.204833236	4.610212452	2.92E-08	7.53
KX728_RS08220		Prolyl-tRNA synthetase (EC 6.1.1.15), bacterial type	2.123474652	4.357421432	3.22E-24	23.49
KX728_RS06775		ABC transporter membrane-spanning permease, Pep export, Vex1	2.112956147	4.325767563	7.73E-20	19.11
KX728_RS00245	purH	IMP cyclohydrolase (EC 3.5.4.10)	2.091420362	4.261674369	3.00E-09	8.52
KX728_RS00240		acetyltransferase, GNAT family	2.080981126	4.230948502	1.85E-07	6.73
KX728_RS08200		hypothetical protein	2.07632019	4.217301571	5.41E-13	12.27
KX728_RS02815		Prophage Lp1 protein 6	2.074915775	4.213198168	2.45E-16	15.61
KX728_RS07240		Fructokinase (EC 2.7.1.4)	2.01387439	4.038653545	2.41E-32	31.62
KX728_RS00255	purE	N5-carboxyaminoimidazole ribonucleotide mutase (EC 5.4.99.18)	2.006705609	4.018635171	6.66182E-06	5.18
KX728_RS07205		hypothetical protein	1.967841998	3.911825452	1.67E-11	10.78
KX728_RS05095		Laminin-binding surface protein	1.955258457	3.877853919	2.06E-18	17.69
KX728_RS00695	rrf	5S ribosomal RNA	1.953662263	3.873565842	3.06956E-06	5.51
KX728_RS01405		hypothetical protein	1.901835397	3.736883006	3.66E-15	14.44
KX728_RS07220		hypothetical protein	1.895836214	3.721376128	3.41E-08	7.47
KX728_RS05100		Streptococcal histidine triad protein	1.87834616	3.676533576	7.68E-17	16.11

KX728_RS02290		Glyoxalase	1.869578993	3.654259259	6.00E-12	11.22
KX728_RS03350		Collagen adhesion protein	1.867872386	3.649939082	9.91E-20	19.00
KX728_RS00260	purK	N5-carboxyaminoimidazole ribonucleotide synthase (EC 6.3.4.18)	1.865770707	3.644625823	4.12E-07	6.39
KX728_RS09040		Thiamin ABC transporter ThiZ, ATPase component	1.79285632	3.465002323	1.21E-21	20.92
KX728_RS06770	vex2	ABC transporter, ATP-binding protein Vex2	1.792067499	3.463108286	7.23E-19	18.14
KX728_RS00960	rrf	5S ribosomal RNA	1.778282974	3.430176884	1.06763E-05	4.97
KX728_RS01930		putative two-component system sensor histidine kinase, putative heat shock protein	1.73314454	3.324516505	1.33E-13	12.88
KX728_RS02370		Late competence protein ComC, processing protease	1.684996047	3.215395153	6.65E-10	9.18
KX728_RS06125		DNA translocase FtsK	1.680360465	3.205080217	1.02562E-05	4.99
KX728_RS01415		lipoprotein, putative	1.608143597	3.048593083	1.43635E-06	5.84
KX728_RS02025	rrf	5S ribosomal RNA	1.605251073	3.042486946	2.76877E-05	4.56
KX728_RS02285		Xanthine phosphoribosyltransferase (EC 2.4.2.22)	1.601345818	3.034262325	8.07E-11	10.09
KX728_RS02010		16S ribosomal RNA	1.594962931	3.020867551	3.47512E-05	4.46
KX728_RS00075		16S ribosomal RNA	1.58997704	3.010445585	3.19217E-05	4.50
KX728_RS00945		16S ribosomal RNA	1.585899461	3.001948988	3.16148E-05	4.50
KX728_RS07210		Type I restriction-modification system, specificity subunit S	1.581716918	2.993258587	3.37E-10	9.47
KX728_RS02505		Antiholin-like protein LrgA	1.58029368	2.990307153	6.75E-16	15.17
KX728_RS00680		16S ribosomal RNA	1.568028844	2.964993298	4.42358E-05	4.35
KX728_RS02360	trpB	Tryptophan synthase beta chain (EC 4.2.1.20)	1.558899721	2.946290573	8.25E-07	6.08
KX728_RS08325	rplQ	LSU ribosomal protein L17p	1.556417173	2.941225042	5.62E-10	9.25
KX728_RS02710		hypothetical protein	1.52889263	2.885642607	1.94E-08	7.71
KX728_RS05960	tRNA-Arg		1.527952553	2.883762901	4.08E-12	11.39
KX728_RS07680		Response regulator CsrR	1.527494865	2.882848185	1.36E-10	9.87
KX728_RS06780	tRNA-Leu		1.526335786	2.880533001	8.73E-15	14.06
KX728_RS02275		hypothetical protein	1.523792843	2.875460148	6.17357E-06	5.21
KX728_RS06590	thiD	Hydroxymethylpyrimidine kinase (EC 2.7.1.49)	1.506196501	2.840601592	2.80E-11	10.55
KX728_RS01665		Putative large secreted protein SCO0341	1.500944299	2.830279044	5.66E-12	11.25
KX728_RS08495	nrdD	Ribonucleotide reductase of class III (anaerobic), large subunit (EC 1.17.4.2)	1.498949885	2.826369107	8.63E-07	6.06
KX728_RS01400		hypothetical protein	1.465712147	2.761997756	1.09E-13	12.96
KX728_RS05365		Uncharacterized protease YrrO	1.461680761	2.754290557	4.01E-17	16.40
KX728_RS01935		FIG01117953: hypothetical protein	1.454634129	2.74087043	7.45E-13	12.13
KX728_RS03740		Putative cI repressor, metallo-proteinase motif (ACLAME 174)	1.441165499	2.71540144	6.33E-12	11.20
KX728_RS07235		Immunoreactive protein Se23.5 (Fragment)	1.438124605	2.709683979	4.01E-20	19.40
KX728_RS08350	infA	Translation initiation factor 1	1.437081298	2.707725138	3.53E-12	11.45
KX728_RS02340		Anthranilate synthase, amidotransferase component (EC 4.1.3.27)	1.423669999	2.682670748	1.77E-07	6.75
KX728_RS09070		Choline binding protein D	1.421223108	2.678124646	3.61E-11	10.44
KX728_RS04245		hypothetical protein	1.41212115	2.661281554	4.55E-07	6.34
KX728_RS05945	cas2	CRISPR-associated protein Cas2	1.409618797	2.656669564	1.4888E-05	4.83
KX728_RS00590		hypothetical protein	1.398316609	2.635938314	3.74E-12	11.43
KX728_RS08990	hslO	33 kDa chaperonin HslO	1.391079545	2.622748634	3.86493E-05	4.41
KX728_RS00540		Lj965 prophage protein	1.389678932	2.620203624	7.06E-10	9.15
KX728_RS05370		Transcriptional regulator in cluster with beta-lactamase, GntR family	1.385217442	2.61211324	1.38E-16	15.86
KX728_RS00450		FIG01119883: hypothetical protein	1.378234706	2.599500993	2.48409E-05	4.60
KX728_RS05360		hypothetical protein	1.372585759	2.589342413	2.64E-13	12.58
KX728_RS00545		hypothetical protein	1.364010335	2.573996933	5.24E-12	11.28
KX728_RS06580		Copper-exporting ATPase (EC 3.6.3.4)	1.335820227	2.524189507	1.73E-12	11.76
KX728_RS02280		Xanthine permease	1.315920561	2.489611382	4.90347E-06	5.31
KX728_RS06555		6-phospho-beta-glucosidase (EC 3.2.1.86)	1.308455402	2.476762274	8.09E-15	14.09
KX728_RS07675		Choline-binding protein	1.29527603	2.454239478	1.07E-14	13.97
KX728_RS02350	trpC	Indole-3-glycerol phosphate synthase (EC 4.1.1.48)	1.294348971	2.452662919	2.68312E-06	5.57
KX728_RS03580		hypothetical protein	1.285295601	2.437319871	1.47E-07	6.83
KX728_RS07580		Acyl-CoA dehydrogenase, short-chain specific (EC 1.3.8.1)	1.285141259	2.437059135	3.38E-14	13.47

KX728_RS05305		TPR-repeat-containing protein	1.283624424	2.434498183	1.70E-09	8.77
KX728_RS02005	tRNA-Glu		1.282320911	2.432299543	5.55E-07	6.26
KX728_RS00265		hypothetical protein	1.280676407	2.429528585	6.29E-10	9.20
KX728_RS07215		hypothetical protein	1.265216669	2.403633059	6.24E-10	9.20
KX728_RS07245		PTS system, sucrose-specific IIB component (EC 2.7.1.211)	1.257156395	2.39024151	2.34E-11	10.63
KX728_RS00315		PTS system, galactosamine-specific IIA component	1.253445882	2.384101871	1.36E-12	11.87
KX728_RS05460		Arginine pathway regulatory protein ArgR, repressor of arg regulon	1.248279482	2.375579491	4.66E-09	8.33
KX728_RS02695		hypothetical protein	1.246256026	2.372249949	4.53E-07	6.34
KX728_RS07905		Undecaprenyl-diphosphatase (EC 3.6.1.27)	1.243028297	2.366948468	2.63E-15	14.58
KX728_RS00535		hypothetical protein	1.238962989	2.360288135	5.35E-12	11.27
KX728_RS00310		PTS system, galactosamine-specific IID component	1.237515612	2.357921375	1.84E-12	11.73
KX728_RS06585		Negative transcriptional regulator-copper transport operon	1.232803679	2.350232817	7.10E-07	6.15
KX728_RS02365	trpA	Tryptophan synthase alpha chain (EC 4.2.1.20)	1.228618785	2.343425263	2.05461E-05	4.69
KX728_RS00275		Serine/threonine kinase	1.222334022	2.333238884	8.10E-10	9.09
KX728_RS05695		MutT/Nudix family protein	1.22212941	2.332907993	2.22181E-05	4.65
KX728_RS02380		Putative oxidoreductase	1.203613207	2.303157716	2.10E-14	13.68
KX728_RS02400		hypothetical protein	1.191265792	2.283530078	1.78E-09	8.75
KX728_RS01475		hypothetical protein	1.174462922	2.257088397	2.67E-09	8.57
KX728_RS01410		putative lipoprotein	1.169564485	2.249437816	3.78524E-05	4.42
KX728_RS07120	ugpC	Maltodextrin ABC transporter, ATP-binding protein MsmX	1.168126493	2.247196828	7.43013E-06	5.13
KX728_RS05060	srtB	NPQTN specific sortase B	1.165593414	2.243254669	1.30E-13	12.89
KX728_RS03585		Alanyl-tRNA synthetase (EC 6.1.1.7)	1.163368542	2.239797869	2.10E-07	6.68
KX728_RS04125		hypothetical protein	1.156705203	2.229476825	1.46316E-05	4.83
KX728_RS00340		hypothetical protein	1.154992705	2.226831976	5.28E-10	9.28
KX728_RS04675		Uracil-DNA glycosylase, family 1 (EC 3.2.2.27)	1.153176269	2.224030032	1.58E-10	9.80
KX728_RS05410	ccdA2	Cytochrome c-type biogenesis protein CcdA homolog	1.148109628	2.216233098	6.60E-13	12.18
KX728_RS01575		hypothetical protein	1.131264374	2.190506315	1.52822E-05	4.82
KX728_RS04535		Phosphoglucomutase (EC 5.4.2.2)	1.129335436	2.187579483	1.69E-14	13.77
KX728_RS08815	sdaAA	L-serine dehydratase, alpha subunit (EC 4.3.1.17)	1.128305234	2.18601793	6.83E-14	13.17
KX728_RS00285		beta-glycosyl hydrolase	1.12045467	2.174154809	9.41E-07	6.03
KX728_RS06355		transcription regulator	1.119665828	2.172966341	2.91689E-05	4.54
KX728_RS01600		hypothetical protein	1.11913932	2.172173467	4.72465E-06	5.33
KX728_RS03755		Phage protein	1.116849449	2.168728491	2.17706E-06	5.66
KX728_RS03210		Transcriptional regulator, Xre family	1.112041423	2.161512864	1.76477E-05	4.75
KX728_RS03735		hypothetical protein	1.109366287	2.157508566	5.95E-10	9.23
KX728_RS00500	ilvD	Dihydroxy-acid dehydratase (EC 4.2.1.9)	1.106551853	2.153303764	3.00E-08	7.52
KX728_RS02705		hypothetical protein	1.105517707	2.151760795	2.1599E-06	5.67
KX728_RS04665		2-haloalkanoic acid dehalogenase (EC 3.8.1.2)	1.086874514	2.124133607	3.04E-09	8.52
KX728_RS07320		UPF0348 protein family	1.07136845	2.101425703	1.58E-10	9.80
KX728_RS04250		GMP reductase (EC 1.7.1.7)	1.066127408	2.093805466	1.00203E-06	6.00
KX728_RS00885	yajC	Protein translocase subunit YajC	1.053991296	2.076266015	6.42E-11	10.19
KX728_RS00305		PTS system, galactosamine-specific IIC component	1.053465817	2.075509905	5.19E-09	8.28
KX728_RS02660		Sialic acid utilization regulator, RpiR family	1.047891138	2.067505457	4.32E-10	9.36
KX728_RS00595		Lead, cadmium, zinc and mercury transporting ATPase (EC 3.6.3.3) (EC 3.6.3.5)	1.037685609	2.052931669	4.41E-08	7.36
KX728_RS00330		Aldose 1-epimerase (EC 5.1.3.3)	1.037495078	2.052660565	5.49E-13	12.26
KX728_RS02725		hypothetical protein	1.036978658	2.051925937	6.09E-08	7.22
KX728_RS07095	tpx	Thiol peroxidase, Tpx-type (EC 1.11.1.15)	1.033287883	2.046683304	4.40E-09	8.36
KX728_RS01460		Putative secretion accessory protein EsaB/YukD	1.015432808	2.021509254	2.91806E-06	5.53
KX728_RS05955	cas9	CRISPR-associated endonuclease Cas9	1.010464951	2.014560246	4.35422E-05	4.36
KX728_RS05465		hypothetical protein	1.005747445	2.007983543	2.71E-10	9.57
KX728_RS06955		Tributyrin esterase	-1.002657568	-2.003687567	4.28658E-06	5.37

KX728_RS01320		Substrate-specific component RibU of riboflavin ECF transporter	-1.018869428	-2.026330398	3.19E-10	9.50
KX728_RS08795		Argininosuccinate synthase (EC 6.3.4.5)	-1.042920745	-2.060394715	9.63687E-06	5.02
KX728_RS07520	fabF	3-oxoacyl-[acyl-carrier-protein] synthase, KASII (EC 2.3.1.179)	-1.047446791	-2.066868769	2.16E-11	10.67
KX728_RS03710		Methylthioadenosine deaminase (EC 3.5.4.31)	-1.047782672	-2.067350023	4.63E-11	10.33
KX728_RS03885		Substrate-specific component BioY of biotin ECF transporter	-1.051240676	-2.072311209	1.01568E-06	5.99
KX728_RS06015		Carbamoyl-phosphate synthase small chain (EC 6.3.5.5)	-1.052169837	-2.073646302	9.12441E-05	4.04
KX728_RS07295	fmt	Methionyl-tRNA formyltransferase (EC 2.1.2.9)	-1.053783496	-2.075966979	4.30E-12	11.37
KX728_RS04110		O-acetylhomoserine sulfhydrylase (EC 2.5.1.49)	-1.055333605	-2.078198708	2.02E-07	6.69
KX728_RS04715		Lipoate-protein ligase A	-1.096329202	-2.13809981	2.97E-11	10.53
KX728_RS04350		Glutamine ABC transporter, substrate-binding protein GlnH	-1.118167286	-2.170710432	3.69E-10	9.43
KX728_RS01780	glnA	Glutamine synthetase type I (EC 6.3.1.2)	-1.125186725	-2.181297767	9.89E-09	8.00
KX728_RS06080		Beta-lactamase class C-like and penicillin binding proteins (PBPs) superfamily	-1.127203707	-2.184349497	3.84E-14	13.42
KX728_RS06075		Permease of the drug/metabolite transporter (DMT) superfamily	-1.128360916	-2.186102302	4.69849E-06	5.33
KX728_RS01550		glycosyl hydrolase family 29 (alpha-L-fucosidase)	-1.155113011	-2.227017679	2.70E-13	12.57
KX728_RS00655		Glutamine amidotransferase, class I	-1.17119556	-2.251982412	5.55E-09	8.26
KX728_RS00580	glgP	Maltodextrin phosphorylase (EC 2.4.1.1)	-1.182036775	-2.268968807	3.89E-12	11.41
KX728_RS05680		Lysyl aminopeptidase (EC 3.4.11.15)	-1.191003785	-2.283115404	4.43E-08	7.35
KX728_RS06990		ABC transporter, permease protein PebF (cluster 3, basic aa/glutamine/opines)	-1.197992591	-2.294202266	4.82089E-06	5.32
KX728_RS07300		Helicase PriA essential for oriC/DnaA-independent DNA replication	-1.209939935	-2.313280054	3.02E-15	14.52
KX728_RS05675	ciaR	Two component system response regulator CiaR	-1.222609988	-2.333685241	2.01E-07	6.70
KX728_RS08130		ABC transporter, permease protein (cluster 3, basic aa/glutamine/opines)	-1.230732817	-2.346861685	5.99575E-05	4.22
KX728_RS03595		Putative ribosomal RNA large subunit methyltransferase YwbD	-1.233657768	-2.35162459	1.79E-12	11.75
KX728_RS02295	xth	Exodeoxyribonuclease III (EC 3.1.11.2)	-1.247532223	-2.374349352	5.80E-21	20.24
KX728_RS04405		Substrate-specific component PanT of predicted pantothenate ECF transporter	-1.251747127	-2.381296272	4.60E-18	17.34
KX728_RS08075	recA	RecA protein	-1.255790122	-2.387978955	3.24E-07	6.49
KX728_RS02240		Hydrolase, HAD superfamily	-1.280330544	-2.428946214	7.80E-08	7.11
KX728_RS07305	rpoZ	DNA-directed RNA polymerase omega subunit (EC 2.7.7.6)	-1.282392443	-2.432420145	2.46E-15	14.61
KX728_RS08140		ABC transporter, substrate-binding protein (cluster 3, basic aa/glutamine/opines)	-1.296857237	-2.456930822	3.84855E-05	4.41
KX728_RS02855		probable esterase of alpha/beta hydrolase superfamily, YBBA B. subtilis ortholog	-1.343306006	-2.537320926	1.02496E-05	4.99
KX728_RS00575	malQ	4-alpha-glucanotransferase (amylomaltase) (EC 2.4.1.25)	-1.348854476	-2.547098012	2.97E-14	13.53
KX728_RS08135		ABC transporter, permease protein (cluster 3, basic aa/glutamine/opines)	-1.39308266	-2.626392728	1.09822E-05	4.96
KX728_RS05780		Late competence protein ComEA, DNA receptor	-1.526538785	-2.880938343	7.07E-07	6.15
KX728_RS02845		Vitamin B12 ABC transporter, ATP-binding protein BtuD	-1.541059246	-2.91008087	5.16E-07	6.29
KX728_RS02860		Acyl-ACP:1-acyl-sn-glycerol-3-phosphate acyltransferase (EC 2.3.1.n4)	-1.62817947	-3.09122672	5.67E-11	10.25
KX728_RS01540		hypothetical protein	-1.66834866	-3.178505667	4.73E-14	13.32
KX728_RS07100		Mn-dependent transcriptional regulator MntR	-1.692286353	-3.231684486	2.24E-20	19.65
KX728_RS03850		Hydrolase, alpha/beta fold family	-1.716531597	-3.286453556	4.13E-13	12.38
KX728_RS06240	pdxS	Pyridoxal 5'-phosphate synthase (glutamine hydrolyzing)	-1.752155503	-3.368614887	1.14E-10	9.94
KX728_RS02475	queE	7-carboxy-7-deazaguanine synthase (EC 4.3.99.3)	-1.810144673	-3.506774526	1.24E-12	11.91
KX728_RS01530		CI-like repressor, phage associated	-1.818559742	-3.527288897	5.16E-16	15.29
KX728_RS02850		Vitamin B12 ABC transporter, substrate-binding protein BtuF	-1.838093452	-3.575372241	5.99E-10	9.22
KX728_RS06245		NADH peroxidase (EC 1.11.1.1)	-1.843522445	-3.588852039	2.73E-12	11.56
KX728_RS04645		hypothetical protein	-1.843590788	-3.589022053	1.62E-08	7.79
KX728_RS01735		Iron compound ABC uptake transporter permease protein PiuB	-1.912929633	-3.76573018	3.09E-08	7.51
KX728_RS01545		hypothetical protein	-1.977967067	-3.939375845	9.61E-25	24.02
KX728_RS01730		Iron compound ABC uptake transporter permease protein PiuC	-1.987874004	-3.966520497	1.32E-09	8.88
KX728_RS01720		Iron compound ABC uptake transporter substrate-binding protein PiuA	-2.082138505	-4.234344076	4.50E-12	11.35
KX728_RS04630		Exopolysaccharide biosynthesis protein	-2.09190019	-4.263092001	2.64E-08	7.58
KX728_RS01535		Putative UV-damage repair protein UvrX	-2.146079642	-4.426233749	5.48E-29	28.26
KX728_RS02610		Sialidase (EC 3.2.1.18)	-2.17396383	-4.512615412	1.24E-07	6.91
KX728_RS04635		hypothetical protein	-2.319922173	-4.993052835	2.30E-08	7.64
KX728_RS08880		Mundticin KS immunity protein	-2.387082171	-5.230983326	1.54E-28	27.81

KX728_RS02490	trxA	Thioredoxin	-2.459023386	-5.498443909	9.69E-27	26.01
KX728_RS02485	queC	7-cyano-7-deazaguanine synthase (EC 6.3.4.20)	-2.487020961	-5.606191247	3.38E-34	33.47
KX728_RS01725		Iron compound ABC uptake transporter ATP-binding protein PiuD	-2.489647425	-5.616406762	9.81E-11	10.01
KX728_RS02480	queD	6-carboxy-5,6,7,8-tetrahydropterin synthase (EC 4.1.2.50)	-2.500909178	-5.660420289	1.13E-26	25.95
KX728_RS02230	galR	Galactose operon repressor, GalR-LacI family of transcriptional regulators	-3.095317533	-8.546404096	2.06E-60	59.69

Supplemental Table 3. DEGs in *Streptococcus mutans* UA159 grown in coculture with *Streptococcus oralis* 34 between TY- vs TY-Saliva**.** Red, upregulated genes. Blue, downregulated genes.

Geneid	Gene	Product	Log2 Fold Change	Fold Change	P value	Log10 P value
SMU_1284c		conserved hypothetical protein	2.864765735	7.284175769	1.17251E-05	4.93
SMU_1185	*mtlA1*	mannitol PTS EII	2.687742709	6.443045171	1.31918E-05	4.88
SMU_910	*gtfD*	glucosyltransferase-S	2.321953509	5.000088079	2.29E-07	6.64
SMU_2147c		conserved hypothetical protein	2.217019708	4.649319943	4.10E-07	6.39
SMU_184	*sloC*	ABC transporter element, iron predicted binding protein	2.161151134	4.472715933	4.56724E-06	5.34
SMU_1419		transcriptional regulator	2.118394979	4.342106101	7.34861E-06	5.13
SMU_1821c		glutamyl-tRNA (Gln) amidotransferase subunit C	2.113995439	4.328884892	8.13911E-06	5.09
SMU_2146c		conserved hypothetical protein	2.057888028	4.163763215	1.5251E-05	4.82
SMU_182	*sloA*	ABC transporter, ATP-binding protein, iron and/or manganese	1.960192048	3.891137733	3.40417E-05	4.47
SMU_1942c		amino acid ABC transporter substrate-binding protein	1.908858238	3.755117987	4.05E-07	6.39
SMU_1124	*pdp*	pyrimidine-nucleoside phosphorylase	1.838245937	3.575750158	7.69339E-05	4.11
SMU_183	*sloB*	manganese ABC transporter permease element	1.79349964	3.466547767	8.47E-07	6.07
SMU_672	*idh*	isocitrate dehydrogenase	1.790525605	3.459409033	2.70E-07	6.57
SMU_673		conserved hypothetical protein	1.751072008	3.366085934	4.5925E-06	5.34
SMU_1822	*gatA*	aspartyl-tRNA synthetase	1.74146718	3.343750448	1.45E-07	6.84
SMU_1390		conserved hypothetical protein	1.740080333	3.340537681	6.52908E-06	5.19
SMU_496	*cysK*	cysteine synthetase A	1.730419604	3.318243145	1.04182E-05	4.98
SMU_866		conserved hypothetical protein	1.609873822	3.052251457	7.15922E-05	4.15
SMU_186	*sloR*	metal-dependent transcriptional regulator (probable DtxR homolog)	1.601348324	3.034267596	3.27397E-06	5.48
SMU_2019	*rl29*	50s ribosomal protein L29	1.588652005	3.007681927	1.97765E-05	4.70
SMU_831		conserved hypothetical protein	1.519315384	2.866549881	2.61E-07	6.58
SMU_80	*hrcA*	heat-inducible transcription repressor	1.512070503	2.852190814	2.6366E-06	5.58
SMU_957		50S ribosomal protein L10	1.492572133	2.813902095	1.52956E-06	5.82
SMU_1565	*malQ*	4-alpha-glucanotransferase	1.490340757	2.809553274	7.76E-07	6.11
SMU_2033c		conserved hypothetical protein	1.489422581	2.807765756	7.21E-07	6.14
SMU_671	*citZ*	citrate synthase	1.47120111	2.772526231	9.82167E-06	5.01
SMU_1514	*rnc*	ribonuclease III	1.414706449	2.666054826	4.49571E-05	4.35
SMU_1877	*ptnA*	mannose PTS system component IIAB	1.393707593	2.62753065	1.0636E-06	5.97
SMU_81	*grpE*	co-chaperone protein GrpE	1.392729337	2.62574959	2.22627E-05	4.65
SMU_670	*citB*	aconitate hydratase; aconitase A	1.389473725	2.619830955	4.56973E-06	5.34
SMU_459		amino acid ABC transporter, substrate-binding protein	1.389224124	2.619377737	5.68259E-05	4.25
SMU_832		hypothetical protein	1.389180444	2.619298432	7.3093E-06	5.14
SMU_941c		conserved hypothetical protein	1.387069038	2.615467853	8.86223E-05	4.05
SMU_1568	*malX*	maltose / maltodextrin-binding protein	1.34247369	2.535857524	6.02695E-05	4.22
SMU_848		conserved hypothetical protein	1.332083426	2.517659931	1.31263E-05	4.88
SMU_649		conserved hypothetical protein	1.323733186	2.503129942	1.60787E-05	4.79
SMU_830	*rgpF*	polysaccharide biosynthesis protein	1.307383943	2.474923518	1.58302E-06	5.80
SMU_1288	*rl19*	50S ribosomal protein L19	1.306813066	2.47394438	5.98806E-06	5.22
SMU_1611c		multi-drug resistance efflux pump, major facilitator superfamily	1.288638285	2.44297362	4.10658E-05	4.39
SMU_1939c		amino acid ABC transport ATP-binding protein	1.27611169	2.421853655	5.2669E-05	4.28
SMU_2166	*rplW*	Ribosomal protein L23	1.260287659	2.395434988	6.36E-07	6.20
SMU_1564	*glgP*	glycogen phosphorylase	1.253671352	2.384474497	4.09483E-06	5.39
SMU_1006		ABC transporter, ATP-binding protein	1.244172492	2.36882643	4.68768E-05	4.33
SMU_1206c		conserved hypothetical protein	1.199750359	2.296999208	5.00521E-05	4.30
SMU_774		hydrolase, haloacid dehalogenase-like	1.192529915	2.285531836	1.20796E-05	4.92
SMU_532	*trpE*	anthranilate synthase, component I	1.181248558	2.267729493	5.69817E-05	4.24

SMU_500		ribosome-associated protein	1.174133117	2.256572479	4.86656E-05	4.31
SMU_2038	*pttB*	phosphotransferase system, trehalose-specific IIBC component (EIIBC-tre)	1.165040227	2.24239468	3.2798E-05	4.48
SMU_697		translation initiation factor IF-3	1.162657511	2.238694259	8.01234E-06	5.10
SMU_460		amino acid ABC transporter, permease	1.155721302	2.227956866	0.000100403	4.00
SMU_1177c		amino acid ABC transporter, amino acid-binding protein	1.138828479	2.202021384	1.86363E-05	4.73
SMU_2037	*treA*	trehalose-6-phosphate hydrolase	1.132342899	2.192144499	4.60745E-06	5.34
SMU_2047	*ptsG*	PTS system, enzyme II, A component	1.13162311	2.191051067	4.57303E-06	5.34
SMU_1879		mannose PTS system component IID	1.124384971	2.180085884	1.87161E-06	5.73
SMU_1178c		amino acid ABC transporter, ATP-binding protein	1.110674425	2.159465736	2.69842E-05	4.57
SMU_1878	*ptnC*	mannose PTS system component IIC	1.09053183	2.12952524	6.03348E-06	5.22
SMU_829	*rgpE*	glycosyltransferase	1.082436703	2.117609691	4.61152E-06	5.34
SMU_2011	*rl6*	50S ribosomal protein L6 (BL10)	1.068378089	2.097074461	2.44814E-06	5.61
SMU_611		ATP-dependent RNA helicase/DEAD family	1.068151812	2.096745576	9.30E-07	6.03
SMU_1940c		peptidase, possible succinyl-diaminopimelic descuccinlyase	1.060221174	2.085251178	5.34848E-05	4.27
SMU_1545c		conserved hypothetical protein	1.057014686	2.080621713	7.75756E-05	4.11
SMU_1179c		amino acid ABC transporter, permease	1.050786727	2.071659251	1.94071E-05	4.71
SMU_834		glycosyltransferase	1.041950871	2.059010048	1.26181E-05	4.90
SMU_835		conserved hypothetical protein (possible membrane protein)	1.04095088	2.057583359	1.01757E-05	4.99
SMU_1820c		glutamyl-tRNA(Gln) amidotransferase subunit A	1.029583841	2.041435295	2.22683E-06	5.65
SMU_1745c		transcriptional regulator, MarR family	1.027782423	2.038887853	3.3796E-05	4.47
SMU_16		amino acid permease	1.013141913	2.018301791	8.56546E-05	4.07
SMU_2016	*rl24*	50S ribosomal protein L24	-1.015809181	-2.022036699	1.90674E-05	4.72
SMU_463	*trxB*	thioredoxin reductase (NADPH)	-1.016601059	-2.023146874	5.07695E-05	4.29
SMU_2112	*gbpA*	glucan-binding protein	-1.029235991	-2.040943142	1.30022E-06	5.89
SMU_2079c		conserved hypothetical protein	-1.032872797	-2.046094526	7.55527E-06	5.12
SMU_1833	*recG*	ATP-dependent DNA helicase	-1.046465727	-2.065463731	8.46767E-06	5.07
SMU_2049c		conserved hypothetical protein	-1.052901872	-2.074698753	3.33456E-05	4.48
SMU_2042	*dexA*	dextranase (1,6-alpha-glucanhydrolase)	-1.05670073	-2.080168982	4.2679E-06	5.37
SMU_610	*spaP*	cell surface antigen	-1.059815047	-2.084664251	2.59719E-05	4.59
SMU_1040c		oxidoreductase, short-chain dehydrogenase/reductase	-1.090446745	-2.129399653	1.71252E-05	4.77
SMU_991		conserved hypothetical protein (similar to ribonucleotide reductase alpha subunit)	-1.094497043	-2.13538624	1.7052E-05	4.77
SMU_527		conserved hypothetical protein	-1.11732101	-2.169437479	6.47E-07	6.19
SMU_1297		conserved hypothetical protein, DHH family	-1.127412277	-2.184665311	1.82803E-05	4.74
SMU_1451	*aldB*	alpha-acetolactate decarboxylase	-1.128909175	-2.186933232	2.50707E-06	5.60
SMU_401c		acetyltransferase	-1.180203902	-2.266088024	3.87243E-06	5.41
SMU_275		L-ribulose 5-phosphate 4-epimerase	-1.187284845	-2.277237631	7.18223E-05	4.14
SMU_400		beta-lactamase family protein	-1.215470963	-2.322165768	3.31E-07	6.48
SMU_1679c		conserved hypothetical protein	-1.227141582	-2.341027013	1.33207E-05	4.88
SMU_32	*purF*	amidophosphoribosyltransferase	-1.22793267	-2.342311044	7.15652E-05	4.15
SMU_1958c		fructose-specific Enzyme IIC component	-1.233164175	-2.350820158	4.67705E-06	5.33
SMU_1382	*leuC*	alpha-isopropylmalate isomerase large subunit	-1.237425117	-2.357773475	1.27088E-06	5.90
SMU_51	*purK*	phosphoribosylaminoimidazole carboxylase, ATPase subunit	-1.241897924	-2.365094655	2.17794E-06	5.66
SMU_270	*sgaT*	ribulose monophosphate PTS pathway enzyme IIC	-1.242895767	-2.366731044	1.19347E-05	4.92
SMU_1706		conserved hypothetical protein	-1.255439715	-2.387399024	4.57324E-05	4.34
SMU_1627	*rl11*	50S ribosomal protein L11	-1.267974674	-2.408232487	5.1827E-05	4.29
SMU_1038c		response regulator	-1.268379127	-2.408907718	5.2196E-06	5.28
SMU_1452	*alsS*	alpha-acetolactate synthase	-1.272133177	-2.415184121	5.26695E-06	5.28
SMU_119	*adh*	alcohol dehydrogenase class III	-1.279926654	-2.428266313	2.02047E-06	5.69
SMU_360	*gapC*	glyceraldehyde-3-phosphate dehydrogenase; plasmin receptor	-1.286747966	-2.439774762	1.06514E-05	4.97
SMU_34	*purM*	phosphoribosylformylglycinamide cyclo-ligase (AIRS)	-1.29060831	-2.446311821	2.60862E-05	4.58
SMU_1383	*leuB*	3-isopropylmalate dehydrogenase	-1.2963552	-2.456075994	1.55236E-06	5.81

SMU_118c		esterase	-1.29795673	-2.458803988	1.97388E-05	4.70
SMU_1384	*leuA*	2-isopropylmalate synthase	-1.307213682	-2.474631456	1.83479E-06	5.74
SMU_54		amino acid racemase	-1.309747293	-2.478981136	8.97818E-06	5.05
SMU_595	*pyrD*	dihydroorotate dehydrogenase (dihydroorotate oxidase)	-1.309932978	-2.479300219	3.65453E-06	5.44
SMU_1602		NAD(P)H-flavin oxidoreductase	-1.314597144	-2.487328652	1.41896E-05	4.85
SMU_1626	*rl1*	50S ribosomal protein L1	-1.337102571	-2.526434139	3.79822E-05	4.42
SMU_1574c		conserved hypothetical protein	-1.349207056	-2.547720574	2.91273E-06	5.54
SMU_2025	*rl3*	50S ribosomal protein L3	-1.400632949	-2.640173883	2.93434E-05	4.53
SMU_924	*tpx*	thiol peroxidase	-1.457748424	-2.746793441	4.17E-07	6.38
SMU_48	*purD*	phosphoribosylamine-glycine ligase	-1.465597859	-2.761778963	8.39E-07	6.08
SMU_37	*purH*	phosphoribosylaminoimidazolecarboxamide formyltransferase / IMP cyclohydrolase	-1.473950843	-2.777815621	5.28E-07	6.28
SMU_1494	*lacC*	tagatose-6-phosphate kinase	-1.537455785	-2.902821348	4.98274E-05	4.30
SMU_1603	*lguL*	lactoylglutathione lyase	-1.601389508	-3.034354215	4.15733E-06	5.38
SMU_2023c		30S ribosomal protein S19, C-terminal fragment	-1.635224231	-3.106358268	4.21977E-06	5.37
SMU_30	*purL*	phosphoribosylformylglycinamidine synthase	-1.648450531	-3.134967597	1.97E-07	6.71
SMU_618		hypothetical protein	-1.669354142	-3.180721688	5.70E-08	7.24
SMU_1489	*lacX*	aldose 1-epimerase	-1.676524695	-3.196570017	1.43533E-06	5.84
SMU_528c		conserved hypothetical protein	-1.702185142	-3.253934345	1.41693E-05	4.85
SMU_629	*sod*	superoxide dismutase	-1.712633614	-3.277585952	2.89E-07	6.54
SMU_59	*purB*	adenylosuccinate lyase	-1.732280524	-3.32252608	1.37E-07	6.86
SMU_2157	*guaB*	inosine-5'- monophosphate dehydrogenase	-1.755695695	-3.376891193	6.30E-09	8.20
SMU_1073	*fthS*	formate--tetrahydrofolate ligase	-1.758916077	-3.384437505	2.47E-07	6.61
SMU_764	*ahpC*	alkyl hydroperoxide reductase, subunit C	-1.762514521	-3.392889679	4.25E-07	6.37
SMU_714		translation elongation factor Tu	-1.788151322	-3.453720471	2.75341E-06	5.56
SMU_1493	*lacD*	tagatose-1,6-bisphosphate aldolase	-1.793865226	-3.467426318	2.12756E-06	5.67
SMU_1860	*rs6*	30S ribosomal protein S6	-1.799706119	-3.48149299	1.41E-07	6.85
SMU_2167	*rplB*	Ribosomal protein L2	-1.807711751	-3.50086578	3.76443E-06	5.42
SMU_1858	*rs18*	30S ribosomal protein S18	-1.813039262	-3.513817498	1.01189E-05	4.99
SMU_2018	*rs17*	30S ribosomal protein S17	-1.864842389	-3.642281402	1.49493E-06	5.83
SMU_1491	*lacE*	PTS system, lactose-specific component IIBC	-1.864939664	-3.642526995	2.01595E-06	5.70
SMU_1490	*lacG*	phospho-beta-D-galactosidase	-1.880126378	-3.681073046	3.96E-07	6.40
SMU_99	*fbaA*	fructose-bisphosphate aldolase	-1.926543422	-3.801433152	1.59623E-06	5.80
SMU_925		bacteriocin immunity protein	-1.93523247	-3.824397469	5.44E-08	7.26
SMU_2002	*rs11*	30S ribosomal protein S11	-1.951094272	-3.866677046	1.13632E-06	5.94
SMU_1247	*eno*	enolase	-1.956921697	-3.882327159	2.05225E-06	5.69
SMU_765		alkyl hydroperoxide reductase, subunit F	-1.959132314	-3.888280541	1.38E-07	6.86
SMU_2021	*rs3*	30S ribosomal protein S3	-1.965554899	-3.905628963	2.27903E-06	5.64
SMU_651c		ABC transporter, periplasmic substrate-binding protein	-1.980339848	-3.945860213	9.29738E-05	4.03
SMU_1492	*lacF*	PTS system, cellobiose-specific IIA component	-1.996062568	-3.989098005	5.54667E-05	4.26
SMU_698		50S ribosomal protein L35	-2.009564796	-4.02660735	1.2338E-06	5.91
SMU_1296		glutathione S-transferase	-2.041912403	-4.117910296	3.46E-07	6.46
SMU_2104a		50S ribosomal protein L32	-2.052211659	-4.147412831	3.92336E-06	5.41
SMU_2015	*rl5*	50S ribosomal protein L5	-2.074661873	-4.212456746	8.59E-07	6.07
SMU_2020	*rl16*	50S ribosomal protein L16	-2.105466714	-4.303369484	2.63253E-06	5.58
SMU_2017	*rl14*	50S ribosomal protein L14	-2.225337637	-4.676203247	9.17E-07	6.04
SMU_2010	*rl18*	50S ribosomal protein L18	-2.233194223	-4.701738243	2.43118E-06	5.61
SMU_1037c		histidine kinase	-2.566059376	-5.921896912	1.59E-09	8.80
SMU_1682c		conserved hypothetical protein (possible intracellular protease)	-2.770532219	-6.823595938	6.02E-07	6.22
SMU_175			-3.112026508	-8.645962073	6.45071E-05	4.19
SMU_996		ABC transporter, permease protein;possible ferrichrome transport system	-3.128731765	-8.746657277	6.79E-10	9.17
SMU_995		ferrichrome ABC transporter (permease)	-3.192363805	-9.14107479	4.65E-10	9.33
SMU_998		ABC transporter, ferrichrome-binding protein	-3.211129551	-9.260753292	2.58E-09	8.59

SMU_997	inorganic ion ABC transporter,ATP-binding protein; possible ferrichrome transport system	-3.248657148	-9.504805786	2.34E-09	8.63
SMU_1764c	conserved hypothetical protein	-3.36334353	-10.29123009	2.44676E-06	5.61
SMU_176	hypothetical protein	-3.368657465	-10.32920608	4.86245E-05	4.31
SMU_1470c	conserved hypothetical protein	-3.497170161	-11.29153847	5.21E-10	9.28
SMU_2027	transcriptional regulator/repressor	-3.525700096	-11.51705616	7.81E-09	8.11
SMU_1761c	conserved hypothetical protein	-3.723177386	-13.20651024	6.42E-08	7.19
SMU_1763c	conserved hypothetical protein	-3.840962276	-14.32995601	6.22E-08	7.21
SMU_1750c	hypothetical protein	-3.856652662	-14.48665555	4.91E-09	8.31
SMU_1762c	conserved hypothetical protein	-3.860899882	-14.52936637	4.36E-08	7.36
SMU_217c	conserved hypothetical protein; Streptococcus-specific protein	-3.982812376	-15.81051411	2.42436E-06	5.62
SMU_1752c	hypothetical protein	-4.142507808	-17.66115522	3.46E-08	7.46
SMU_932	conserved hypothetical protein	-4.177333571	-18.09267186	2.76E-08	7.56
SMU_1760c	conserved hypothetical protein	-4.191425154	-18.27025867	6.82E-09	8.17
SMU_1757c	conserved hypothetical protein	-4.215661513	-18.57977999	2.57E-08	7.59
SMU_961	macrophage infectivity potentiator-related protein	-4.409685709	-21.25434226	3.58E-09	8.45
SMU_1755c	conserved hypothetical protein	-4.413690095	-21.31341838	1.36E-09	8.87
SMU_1758c	conserved hypothetical protein	-4.442797452	-21.74779836	8.35E-09	8.08
SMU_936	amino acid ABC transporter, ATP-binding protein	-4.478416261	-22.29141451	1.44E-07	6.84
SMU_935	amino acid ABC transporter, permease protein	-4.506700334	-22.73275032	4.98E-09	8.30
SMU_933	amino acid ABC transporter, amino acid substrate-binding protein	-4.577178462	-23.87085708	1.12E-07	6.95
SMU_191c	phage-related integrase	-4.602476667	-24.29313319	2.09E-07	6.68
SMU_962	acyl-CoA dehydrogenase	-4.619620473	-24.58353491	1.37E-09	8.86
SMU_195c	hypothetical protein	-4.642631101	-24.97877984	4.14E-07	6.38
SMU_1754c	conserved hypothetical protein	-4.647998181	-25.07187837	5.13E-10	9.29
SMU_934	amino acid ABC transporter, permease protein	-4.650404314	-25.11372825	1.68E-08	7.77
SMU_1753c	conserved hypothetical protein	-4.683232169	-25.6917306	6.47E-09	8.19
SMU_196c	immunogenic secreted protein (transfer protein)	-4.964494532	-31.22207535	3.52E-10	9.45
SMU_194c	conserved hypothetical protein, phage-related	-5.015345414	-32.34218881	2.21488E-06	5.65
SMU_215c	hypothetical protein	-5.25254536	-38.12182708	2.95E-07	6.53
SMU_216c	hypothetical protein	-5.691541616	-51.68026692	6.16E-07	6.21
SMU_200c	hypothetical protein	-6.131075572	-70.08702938	3.03E-07	6.52
SMU_198c	conjugative transposon protein	-6.300662506	-78.82943376	1.01E-09	8.99
SMU_213c	hypothetical protein	-6.351137642	-81.63622928	2.28839E-06	5.64
SMU_212c	hypothetical protein	-6.354243385	-81.81215994	2.53255E-06	5.60
SMU_199c	hypothetical protein	-6.435304082	-86.54053186	1.12E-09	8.95
SMU_206c	hypothetical protein	-6.4607037	-88.07762733	4.41E-07	6.36
SMU_197c	hypothetical protein	-6.474903618	-88.94882347	2.26E-11	10.65
SMU_204c	hypothetical protein	-6.705046802	-104.3326431	5.18E-09	8.29
SMU_214c	hypothetical protein	-6.921377529	-121.2110595	1.74E-07	6.76
SMU_202c	conserved hypothetical protein/Streptococcus-specific protein	-6.940149763	-122.7985541	1.31E-09	8.88
SMU_205c	conserved hypothetical protein	-7.104055285	-137.5731659	1.71E-08	7.77
SMU_210c	hypothetical protein	-7.273954601	-154.7670562	1.12E-07	6.95
SMU_211c	hypothetical protein	-7.62219843	-197.0200207	1.21E-09	8.92
SMU_208c	conserved hypothetical protein, FtsK/SpoIIIE family	-7.680653949	-205.1668662	2.80E-11	10.55
SMU_207c	transcriptional regulator	-7.751483451	-215.490947	3.65E-10	9.44
SMU_209c	hypothetical protein	-7.789318636	-221.2170289	2.13E-09	8.67
SMU_201c	conserved hypothetical protein	-8.018553668	-259.3135293	1.38E-08	7.86

Supplemental Table 4. *Streptococcus mutans* UA159 DEGs during coculture with *Streptococcus oralis* 34 in either TY-, TY-Saliva, or both. Light grey,TY- only; Red, TY-Saliva; Dark blue, both.

TY			TY-Saliva		
Gene ID	Gene	Product	Geneid	Gene	Product
			SMU_05		conserved hypothetical protein
			SMU_102		PTS system, IID component
			SMU_103		PTS system, IIA component
			SMU_104		glycosyl hydrolase, alpha-glucosidase
			SMU_105		SCR operon transcriptional repressor
			SMU_106c		
SMU_1036		conserved hypothetical protein			
SMU_1037c		histidine kinase			
SMU_1063	*opuAa*	amino acid ABC transporter, ATP-binding protein			
			SMU_1073	*fthS*	formate--tetrahydrofolate ligase
			SMU_1077	*pgm*	phosphoglucomutase
			SMU_1090		conserved hypothetical protein
			SMU_1091	*wapE*	hypothetical protein (possible cell wall protein)
SMU_1093		ABC transporter permease protein			
			SMU_110	*mutR*	transcriptional regulator
SMU_1131c		hypothetical protein			
			SMU_1175		sodium:alanine (or glycine) symporter
SMU_1177c		amino acid ABC transporter, amino acid-binding protein			
SMU_1178c		amino acid ABC transporter, ATP-binding protein			
SMU_1189c		conserved hypothetical protein			
SMU_1197		conserved hypothetical protein			
			SMU_120		50S ribosomal protein L28
SMU_1223	*pyrDB*	dihydroorotate dehydrogenase			
SMU_1246c		transcriptional regulator (TetR/AcrR family)			
SMU_1254		phosphatase			
			SMU_1260c		conserved hypothetical protein
			SMU_1262c		hypothetical protein
SMU_132		amino acid amidohydrolase (hippurate amidohydrolase)			
SMU_1326	*rf2*	peptide chain release factor			
			SMU_1396	*gbpC*	glucan-binding protein C
			SMU_1400c		conserved hypothetical protein
			SMU_1403c		conserved hypothetical protein
			SMU_1425	*clpB*	ATP-dependent Clp protease, ATP-binding subunit ClpB
SMU_1470c		conserved hypothetical protein			
			SMU_148	*adhE*	alcohol-acetaldehyde dehydrogenase
			SMU_1488c		conserved hypothetical protein
			SMU_1489	*lacX*	aldose 1-epimerase
			SMU_149		transposase fragment (IS605/IS200-like)
			SMU_1490	*lacG*	phospho-beta-D-galactosidase
			SMU_1491	*lacE*	PTS system, lactose-specific component IIBC
			SMU_1492	*lacF*	PTS system, cellobiose-specific IIA component
			SMU_1493	*lacD*	tagatose-1,6-bisphosphate aldolase
			SMU_1494	*lacC*	tagatose-6-phosphate kinase
			SMU_1495	*lacB*	galactose-6-phosphate isomerase
			SMU_1496	*lacA*	galactose-6-phosphate isomerase
SMU_150		non-lantibiotic mutacin IV A			

SMU_151		non-lantibiotic mutacin IV B			
SMU_152		hypothetical protein			
			SMU_1535	*phsG*	glycogen phosphorylase
			SMU_1536	*glgA*	glycogen synthase
			SMU_1537	*glgD*	glycogen biosynthesis protein
			SMU_1538	*glgC*	glucose-1-phosphate adenylyltransferase
			SMU_1539	*glgB*	1,4-alpha-glucan branching enzyme
SMU_1546		conserved hypothetical protein			
			SMU_1596	*ptcC*	PTS system, cellobiose-specific IIC component
			SMU_1599	*celR*	transcriptional regulator, required for cellobiose uptake and metabolism
			SMU_1600	*ptcB*	PTS system IIB component, required for cellobiose uptake and metabolism
			SMU_1601	*bgl*	6-phospho-beta-glucosidase, required for cellobiose uptake and metabolism
SMU_1602		NAD(P)H-flavin oxidoreductase			
			SMU_1604c		conserved hypothetical protein
SMU_1626	*rl1*	50S ribosomal protein L1			
			SMU_1641c		conserved hypothetical protein
SMU_1657c		nitrogen regulatory protein PII	SMU_1657c		nitrogen regulatory protein PII
SMU_1658	*nrgA*	ammonium transporter, NrgA protein	SMU_1658	*nrgA*	ammonium transporter, NrgA protein
SMU_1682c		conserved hypothetical protein			
SMU_169		50S ribosomal protein L13			
SMU_1734	*accA*	acetyl-CoA carboxylase alpha subunit			
SMU_1745c		transcriptional regulator, MarR family			
SMU_1750c		hypothetical protein			
SMU_1752c		hypothetical protein	SMU_1752c		hypothetical protein
SMU_1753c		conserved hypothetical protein			
SMU_1754c		conserved hypothetical protein	SMU_1754c		conserved hypothetical protein
SMU_1755c		conserved hypothetical protein			
SMU_1757c		conserved hypothetical protein	SMU_1757c		conserved hypothetical protein
SMU_1758c		conserved hypothetical protein			
SMU_1760c		conserved hypothetical protein	SMU_1760c		conserved hypothetical protein
SMU_1761c		conserved hypothetical protein	SMU_1761c		conserved hypothetical protein
SMU_1762c		conserved hypothetical protein	SMU_1762c		conserved hypothetical protein
SMU_1763c		conserved hypothetical protein	SMU_1763c		conserved hypothetical protein
SMU_1764c		conserved hypothetical protein	SMU_1764c		conserved hypothetical protein
			SMU_1774c		conserved hypothetical protein
			SMU_1775c		hypothetical protein
			SMU_179		conserved hypothetical protein (possible oxidoreductase)
			SMU_180		oxidoreductase, possible fumarate reductase
SMU_1802c		conserved hypothetical protein			
			SMU_182	*sloA*	ABC transporter, ATP-binding protein, iron and/or manganese
			SMU_183	*sloB*	manganese ABC transporter permease element
			SMU_184	*sloC*	ABC transporter element, iron predicted binding protein
			SMU_185		hypothetical protein
SMU_1853		conserved hypothetical protein			
			SMU_1858	*rs18*	30S ribosomal protein S18
			SMU_186	*sloR*	metal-dependent transcriptional regulator (probable DtxR homolog)
			SMU_1860	*rs6*	30S ribosomal protein S6
			SMU_1862		hypothetical protein
			SMU_1877	*ptnA*	mannose PTS system component IIAB
			SMU_1878	*ptnC*	mannose PTS system component IIC
			SMU_1879		mannose PTS system component IID
SMU_1889c		hypothetical protein (possible relation to bacteriocin BlpU)			
			SMU_189		hypothetical protein

			SMU_1895c		hypothetical protein
			SMU_1896c		hypothetical protein
SMU_191c		phage-related integrase			
SMU_193c		conserved hypothetical protein			
			SMU_1941	*atmB*	ABC transporter solute-binding protein
			SMU_1942c		amino acid ABC transporter substrate-binding protein
SMU_194c		conserved hypothetical protein, phage-related			
SMU_1945		conserved hypothetical protein			
			SMU_1955	*groES*	co-chaperonin 10kDa
SMU_1956c		conserved hypothetical protein	SMU_1956c		conserved hypothetical protein
SMU_1957		fructose-specific Enzyme IID component	SMU_1957		fructose-specific Enzyme IID component
SMU_1958c		fructose-specific Enzyme IIC component	SMU_1958c		fructose-specific Enzyme IIC component
SMU_195c		hypothetical protein			
SMU_1960c		fructose-specific Enzyme IIB component	SMU_1960c		fructose-specific Enzyme IIB component
SMU_1961c		fructose-specific Enzyme IIA component	SMU_1961c		fructose-specific Enzyme IIA component
SMU_196c		immunogenic secreted protein (transfer protein)			
SMU_197c		hypothetical protein			
SMU_198c		conjugative transposon protein			
SMU_199c		hypothetical protein	SMU_1988c		probable DNA binding protein
SMU_200c		hypothetical protein			
SMU_2002	*rs11*	30S ribosomal protein S11	SMU_2002	*rs11*	30S ribosomal protein S11
SMU_2010	*rl18*	50S ribosomal protein L18	SMU_2010	*rl18*	50S ribosomal protein L18
			SMU_2015	*rl5*	50S ribosomal protein L5
SMU_2017	*rl14*	50S ribosomal protein L14	SMU_2017	*rl14*	50S ribosomal protein L14
SMU_2018	*rs17*	30S ribosomal protein S17	SMU_2018	*rs17*	30S ribosomal protein S17
SMU_201c		conserved hypothetical protein			
			SMU_2020	*rl16*	50S ribosomal protein L16
			SMU_2023c		30S ribosomal protein S19, C-terminal fragment
SMU_2027		transcriptional regulator/repressor	SMU_2027		transcriptional regulator/repressor
SMU_2028	*sacB*	fructosyltransferase			
SMU_202c		conserved hypothetical protein/Streptococcus-specific protein	SMU_202c		conserved hypothetical protein/Streptococcus-specific protein
			SMU_2037	*treA*	trehalose-6-phosphate hydrolase
			SMU_2038	*pttB*	phosphotransferase system, trehalose-specific IIBC component (EIIBC-tre)
SMU_204c		hypothetical protein			
SMU_205c		conserved hypothetical protein			
SMU_206c		hypothetical protein			
SMU_207c		transcriptional regulator			
SMU_208c		conserved hypothetical protein, FtsK/SpoIIIE family			
SMU_209c		hypothetical protein			
SMU_210c		hypothetical protein			
			SMU_2104a		50S ribosomal protein L32
SMU_2117	*opuCb*	glycine betaine / carnitine / choline ABC transporter permease			
SMU_211c		hypothetical protein			
SMU_212c		hypothetical protein			
			SMU_2127		succinic semialdehyde dehydrogenase
SMU_2146c		conserved hypothetical protein			
SMU_2147c		conserved hypothetical protein	SMU_2147c		conserved hypothetical protein
SMU_215c		hypothetical protein			
			SMU_2154c		peptidase, M16 family
			SMU_217c		conserved hypothetical protein; Streptococcus-specific protein
SMU_231	*ilvB*	acetolactate synthase, large subunit (AHAS)			
			SMU_262	*otcA*	ornithine carbamoyltransferase
			SMU_263		amino acid permease /putrescine antiporter

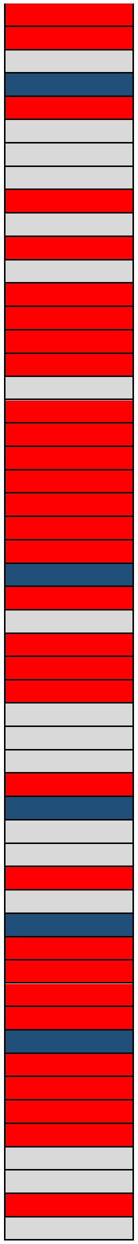

			SMU_267c		glutamate--cysteine ligase (gamma ECS)
			SMU_27	*acpP*	acyl carrier protein
SMU_272	*ptxA*	PTS system, enzyme IIA component			
SMU_299c		bacteriocin peptide precursor	SMU_299c		bacteriocin peptide precursor
			SMU_30	*purL*	phosphoribosylformylglycinamidine synthase
SMU_358		30S ribosomal protein S7			
SMU_361	*pgk*	phosphoglycerate kinase			
SMU_367		Streptococcus-specific protein; similar to glucan-binding protein			
			SMU_378		hypothetical protein
SMU_40		conserved hypothetical protein			
			SMU_402	*pfl*	pyruvate formate-lyase
SMU_412c		Hit-like protein			
			SMU_43		conserved hypothetical protein
			SMU_438c		(R)-2-hydroxyglutaryl-CoA dehydratase activator-related protein
			SMU_44		conserved hypothetical protein
			SMU_45		hypothetical protein
SMU_454	*ftsL*	cell division protein			
			SMU_459		amino acid ABC transporter, substrate-binding protein
			SMU_460		amino acid ABC transporter, permease
			SMU_461		amino acid ABC transporter, ATP-binding protein
			SMU_48	*purD*	phosphoribosylamine-glycine ligase
			SMU_491		transcriptional regulator, DeoR family
			SMU_494		transaldolase family protein
			SMU_495	*gldA*	glycerol dehydrogenase
SMU_496	*cysK*	cysteine synthetase A	SMU_496	*cysK*	cysteine synthetase A
			SMU_50	*purE*	phosphoribosylaminoimidazole carboxylase catalytic subunit
SMU_525		ABC transporter ATP-binding / permease protein			
			SMU_56		Streptococcus-specific protein
			SMU_576	*lytR*	response regulator
			SMU_577	*lytS*	sensor histidine kinase
SMU_586		conserved hypothetical protein, DevG family			
SMU_600c		conserved hypothetical protein			
SMU_627		conserved hypothetical protein			
			SMU_628		DNA polymerase III, delta subunit
SMU_635		conserved hypothetical protein	SMU_635		conserved hypothetical protein
SMU_651c		ABC transporter, periplasmic substrate-binding protein			
SMU_652c		ABC transporter, ATP-binding protein (possible nitrate transport system)			
			SMU_675		phosphoenolpyruvate-protein phosphotransferase (enzyme I)
SMU_689		autolysin/endolysin			
SMU_698		50S ribosomal protein L35	SMU_698		50S ribosomal protein L35
			SMU_714		translation elongation factor Tu
			SMU_735		hypothetical protein
			SMU_768c		conserved hypothetical protein
			SMU_769		conserved hypothetical protein
SMU_770c		manganese transporter	SMU_770c		manganese transporter/possible HitA ferric iron-binding periplasmic protein
			SMU_78	*fruA*	fructan hydrolase; exo-beta-D-fructosidase
			SMU_79	*fruB*	fructan hydrolase; exo-beta-D-fructosidase
			SMU_81	*grpE*	co-chaperone protein GrpE
			SMU_82	*dnaK*	chaperone protein, DnaK
SMU_84	*truA*	tRNA pseudouridine synthase A			
SMU_867	*rimM*	16S rRNA processing protein			
			SMU_872		fructose-specific PTS system enzyme IIBC component
SMU_873	*metE*	5-methyltetrahydropteroyltriglutamate--homocysteine S-methyltransferase			

SMU_909	malate permease				
			SMU_930c		transcriptional regulator
SMU_932	conserved hypothetical protein		SMU_932		conserved hypothetical protein
SMU_933	amino acid ABC transporter, amino acid substrate-binding protein		SMU_933		amino acid ABC transporter, amino acid substrate-binding protein
SMU_934	amino acid ABC transporter, permease protein		SMU_934		amino acid ABC transporter, permease protein
SMU_935	amino acid ABC transporter, permease protein		SMU_935		amino acid ABC transporter, permease protein
SMU_936	amino acid ABC transporter, ATP-binding protein		SMU_936		amino acid ABC transporter, ATP-binding protein
			SMU_940c		hemolysin III-related protein
SMU_957	50S ribosomal protein L10				
SMU_959c	hypothetical protein				
SMU_961	macrophage infectivity potentiator-related protein				
SMU_962	acyl-CoA dehydrogenase				
			SMU_980	*bglP*	beta-glucoside-specific EII permease
			SMU_999		hypothetical protein

Supplemental Table 5. DEGs in *Streptococcus oralis* 34 grown in coculture with *Streptococcus mutans* UA159 between TY- vs TY-Saliva**.** Red, upregulated genes. Blue, downregulated genes.

Gene ID	Gene Name	Gene Description	Log2 Fold Change	Fold Change	P value	Log10 P value
KX728_RS08895		ABC transporter substrate-binding protein	8.594658788	386.5895388	5.36E-135	134.27
KX728_RS08905		sugar ABC transporter permease	7.978349892	252.186964	2.36E-168	167.63
KX728_RS08900		carbohydrate ABC transporter permease	7.425799661	171.9445599	2.15E-141	140.67
KX728_RS08885		bacteriocin immunity protein	6.849969947	115.3576562	5.42E-64	63.27
KX728_RS08890		CPBP family intramembrane metalloprotease	5.638925535	49.82940819	3.35E-65	64.47
KX728_RS06730		S8 family serine peptidase	5.592911853	48.26521342	7.73E-18	17.11
KX728_RS02155		MarR family transcriptional regulator	3.734372888	13.30939316	7.97E-61	60.10
KX728_RS02160		ABC transporter ATP-binding protein	3.677840918	12.79795078	6.40E-52	51.19
KX728_RS02165		ABC transporter permease/substrate-binding protein	3.64125563	12.47748816	2.28E-43	42.64
KX728_RS00335		VOC family protein	3.517143232	11.44894877	4.17E-35	34.38
KX728_RS04325		VIT family protein	3.318274812	9.974709404	8.10E-38	37.09
KX728_RS06775	vex2	ABC transporter ATP-binding subunit Vex2	3.21408834	9.279765444	1.19E-39	38.92
KX728_RS07065		ferric reductase-like transmembrane domain-containing protein	3.133894995	8.778016576	1.13E-27	26.95
KX728_RS08940		alpha-L-fucosidase	2.921151292	7.57450333	5.10E-37	36.29
KX728_RS06770	vex3	ABC transporter permease subunit Vex3	2.780001048	6.86852848	9.15E-40	39.04
KX728_RS06860		DNA starvation/stationary phase protection protein	2.770436653	6.823143948	8.72E-43	42.06
KX728_RS02500		DUF4649 family protein	2.748995886	6.722490847	5.52E-36	35.26
KX728_RS02495	trxA	thioredoxin	2.740237763	6.681804459	8.12E-38	37.09
KX728_RS06780		ABC transporter permease	2.662453987	6.331090364	5.64E-38	37.25
KX728_RS03350		LPXTG-anchored adhesin/beta-galactosidase BgaA	2.479929477	5.578701957	4.08E-32	31.39
KX728_RS08220		glycoside hydrolase family 1 protein	2.454596315	5.481597181	4.69E-31	30.33
KX728_RS00240		GNAT family N-acetyltransferase	2.192728694	4.57169353	8.16E-08	7.09
KX728_RS00225	purF	amidophosphoribosyltransferase	2.150928601	4.441135538	1.49E-07	6.83
KX728_RS05075		phosphate-transport permease PitB	2.060081583	4.170098851	1.54E-17	16.81
KX728_RS00235	purN	phosphoribosylglycinamide formyltransferase	2.029800409	4.083483529	4.98E-07	6.30
KX728_RS05085	pitA	DUF5979 domain-containing protein	2.018806003	4.052482633	4.52E-23	22.35
KX728_RS00255	purE	5-(carboxyamino)imidazole ribonucleotide mutase	1.985205672	3.959191012	9.10935E-06	5.04
KX728_RS03030		NAD(P)H-dependent oxidoreductase	1.965554425	3.90562768	2.93E-24	23.53
KX728_RS08205		hypothetical protein	1.943849719	3.847309034	2.02E-10	9.70
KX728_RS00230	purM	phosphoribosylformylglycinamidine cyclo-ligase	1.873513636	3.664239076	1.15911E-05	4.94
KX728_RS02505		MarR family transcriptional regulator	1.783269439	3.442053303	1.36E-19	18.87
KX728_RS01665		glycoside hydrolase N-terminal domain-containing protein	1.752294577	3.368939631	1.38E-15	14.86
KX728_RS00260	purK	5-(carboxyamino)imidazole ribonucleotide synthase	1.752197519	3.368712992	1.91126E-06	5.72
KX728_RS00245	purH	bifunctional phosphoribosylaminoimidazolecarboxamide formyltransferase	1.736370218	3.331958005	7.27E-07	6.14
KX728_RS05080	sipA	PI-2 pilus system signal peptidase SipA	1.720812896	3.296220824	3.49E-20	19.46
KX728_RS05065	srtB	class B sortase	1.697254888	3.242833357	4.76E-18	17.32
KX728_RS05070	srtB	class B sortase	1.681768865	3.208210636	5.19E-17	16.28
KX728_RS05095		pneumococcal-type histidine triad protein	1.588943953	3.008290631	6.21E-13	12.21
KX728_RS05105		pneumococcal-type histidine triad protein	1.588470527	3.007303611	1.24E-09	8.91
KX728_RS06555	manA	mannose-6-phosphate isomerase%2C class I	1.565109061	2.958998696	3.01E-20	19.52
KX728_RS00285		LPXTG-anchored beta-N-acetylhexosaminidase StrH	1.560396091	2.949348065	1.63E-11	10.79
KX728_RS02370	trpA	tryptophan synthase subunit alpha	1.543939419	2.915896311	1.46E-08	7.83
KX728_RS04430		hypothetical protein	1.496376219	2.821331558	3.72635E-06	5.43
KX728_RS02290		xanthine phosphoribosyltransferase	1.489493378	2.807903545	3.01E-08	7.52
KX728_RS05100		zinc ABC transporter substrate-binding protein	1.458073221	2.747411903	5.59E-11	10.25
KX728_RS07210		hypothetical protein	1.435388383	2.704549651	6.29E-09	8.20

KX728_RS06750		hypothetical protein	1.429062367	2.692716541	7.64E-08	7.12
KX728_RS02815		G5 domain-containing protein	1.384204006	2.610278979	2.33E-08	7.63
KX728_RS06350		DUF6161 domain-containing protein	1.383948184	2.609816159	3.3961E-06	5.47
KX728_RS00500	ilvD	dihydroxy-acid dehydratase	1.373954783	2.591800698	8.05E-12	11.09
KX728_RS05365		CHY zinc finger protein	1.369157272	2.583196287	2.93E-15	14.53
KX728_RS09230		TetR/AcrR family transcriptional regulator	1.319536575	2.495859245	1.54345E-05	4.81
KX728_RS07205		hypothetical protein	1.319109513	2.495120537	3.66774E-06	5.44
KX728_RS08910		beta-N-acetylhexosaminidase	1.313168566	2.484866881	1.28E-08	7.89
KX728_RS09040		LPXTG cell wall anchor domain-containing protein	1.308979163	2.47766161	1.51E-12	11.82
KX728_RS07245		ROK family protein	1.294425187	2.452792494	6.33E-12	11.20
KX728_RS07235		type I restriction endonuclease subunit R	1.293345305	2.450957223	1.25E-16	15.90
KX728_RS02350	trpD	anthranilate phosphoribosyltransferase	1.259318469	2.393826296	4.20769E-06	5.38
KX728_RS08200		hypothetical protein	1.255137641	2.386899199	6.00457E-06	5.22
KX728_RS04535		thioredoxin	1.245197417	2.370509898	3.26E-17	16.49
KX728_RS02285		purine permease	1.240521913	2.362839956	3.74E-07	6.43
KX728_RS08920		alpha-mannosidase	1.233427494	2.351249267	1.79E-07	6.75
KX728_RS07120		bifunctional 2'%2C3'-cyclic-nucleotide 2'-phosphodiesterase/3'-nucleotidase	1.217849224	2.32599698	3.11964E-06	5.51
KX728_RS05370		U32 family peptidase	1.206423916	2.307649181	4.85E-13	12.31
KX728_RS02340	trpE	anthranilate synthase component I	1.199571909	2.296715104	9.46704E-06	5.02
KX728_RS05155		transcription antiterminator	1.162716837	2.23878632	6.04529E-06	5.22
KX728_RS01405		hypothetical protein	1.150402986	2.219758899	1.11293E-06	5.95
KX728_RS07240		TVP38/TMEM64 family protein	1.14273449	2.207991304	5.84E-12	11.23
KX728_RS02280		endonuclease	1.126964283	2.183987021	6.86412E-05	4.16
KX728_RS08915		ROK family protein	1.125295726	2.181462579	2.87319E-05	4.54
KX728_RS07075		hypothetical protein	1.109478649	2.157676607	2.46E-12	11.61
KX728_RS00310		PTS system mannose/fructose/sorbose family transporter subunit IID	1.077137795	2.109846141	7.42E-10	9.13
KX728_RS05020		4-oxalocrotonate tautomerase	1.073160208	2.104037194	2.90E-07	6.54
KX728_RS05195		PTS sugar transporter subunit IIA	1.058248734	2.08240219	4.84E-08	7.32
KX728_RS04125		DEAD/DEAH box helicase	1.051245111	2.072317579	7.72256E-05	4.11
KX728_RS00315		PTS sugar transporter subunit IIA	1.048521943	2.068409654	2.55E-09	8.59
KX728_RS02400		Rrf2 family transcriptional regulator	1.047590392	2.067074509	1.15E-07	6.94
KX728_RS08325		ACT domain-containing protein	1.045165004	2.063602363	2.3933E-05	4.62
KX728_RS03740		AAA family ATPase	1.038749521	2.054446157	5.57E-07	6.25
KX728_RS05060	hemH	ferrochelatase	1.027872537	2.03901521	5.55E-11	10.26
KX728_RS07730		beta-N-acetylglucosaminidase domain-containing protein	1.024537102	2.034306559	9.43514E-06	5.03
KX728_RS08805		ABC transporter substrate-binding protein	-1.015249382	-2.021252253	1.07773E-05	4.97
KX728_RS04055		cysteine desulfurase	-1.0231731	-2.032384125	3.49E-11	10.46
KX728_RS06080		EamA family transporter	-1.050139232	-2.070729681	1.53E-12	11.81
KX728_RS02470		aquaporin	-1.052375609	-2.073942088	1.03E-08	7.99
KX728_RS08705		M20/M25/M40 family metallo-hydrolase	-1.065773519	-2.093291924	1.81E-14	13.74
KX728_RS08800		argininosuccinate synthase	-1.076360093	-2.10870911	2.41E-07	6.62
KX728_RS03410		CshA/CshB family fibrillar adhesin-related protein	-1.099059404	-2.142149849	3.78E-13	12.42
KX728_RS09005		DUF1275 family protein	-1.154040126	-2.225362135	1.08E-09	8.97
KX728_RS03595		alpha-amylase	-1.160287131	-2.235019055	3.18E-11	10.50
KX728_RS04355		ABC transporter substrate-binding protein/permease	-1.209381243	-2.312384398	1.08E-12	11.97
KX728_RS08210	glmS	glutamine--fructose-6-phosphate transaminase (isomerizing)	-1.254474317	-2.385802	3.21E-10	9.49
KX728_RS06240	pdxT	pyridoxal 5'-phosphate synthase glutaminase subunit PdxT	-1.271839986	-2.414693347	2.36481E-06	5.63
KX728_RS09130	cysK	cysteine synthase A	-1.305594729	-2.471856049	1.13E-07	6.95
KX728_RS03360		Cna B-type domain-containing protein	-1.311886196	-2.482659136	2.97E-10	9.53
KX728_RS04350		amino acid ABC transporter ATP-binding protein	-1.34378575	-2.538164809	6.77E-14	13.17
KX728_RS06245	pdxS	pyridoxal 5'-phosphate synthase lyase subunit PdxS	-1.354340111	-2.556801413	2.06E-07	6.69

KX728_RS06980		amino acid ABC transporter ATP-binding protein	-1.385399359	-2.612442637	1.97E-08	7.71
KX728_RS01540		hypothetical protein	-1.407811474	-2.65334353	1.41E-10	9.85
KX728_RS07830		peptide ABC transporter substrate-binding protein	-1.462539261	-2.755930031	3.75E-12	11.43
KX728_RS04630		bifunctional glycosyltransferase family 2/GtrA family protein	-1.468265608	-2.76689061	6.86921E-05	4.16
KX728_RS00575	malQ	4-alpha-glucanotransferase	-1.46830233	-2.76696104	1.64E-16	15.78
KX728_RS01545		DUF5960 family protein	-1.473373239	-2.776703705	8.20E-15	14.09
KX728_RS04645		peptidase	-1.475744817	-2.78127195	5.70922E-06	5.24
KX728_RS06985		transporter substrate-binding domain-containing protein	-1.547519309	-2.923140774	3.76E-10	9.43
KX728_RS06995		amino acid ABC transporter permease	-1.589534921	-3.009523163	1.95E-08	7.71
KX728_RS07995		hypothetical protein	-1.67538197	-3.194039092	9.21204E-05	4.04
KX728_RS06990		amino acid ABC transporter permease	-1.678781217	-3.201573692	2.50E-10	9.60
KX728_RS00655		gamma-glutamyl-gamma-aminobutyrate hydrolase family protein	-1.681462541	-3.207529515	1.48E-16	15.83
KX728_RS04635		phosphodiester glycosidase family protein	-1.791304326	-3.461276816	1.1182E-05	4.95
KX728_RS07585		acyl-CoA dehydrogenase family protein	-1.794512366	-3.468982027	1.37E-11	10.86
KX728_RS01530		XRE family transcriptional regulator	-1.831952874	-3.560186636	3.43E-16	15.47
KX728_RS01730		iron chelate uptake ABC transporter family permease subunit	-1.835180204	-3.56815974	3.51E-08	7.46
KX728_RS07100	tpx	thiol peroxidase	-1.875027437	-3.668085933	2.23E-24	23.65
KX728_RS01535		Y-family DNA polymerase	-1.899969694	-3.732053567	1.98E-23	22.70
KX728_RS07590		carboxymuconolactone decarboxylase family protein	-1.925056157	-3.797516299	1.56E-11	10.81
KX728_RS02855		ABC transporter substrate-binding protein	-2.004799632	-4.013329566	1.08E-10	9.97
KX728_RS02475	queF	preQ(1) synthase	-2.099964536	-4.286988468	3.33E-16	15.48
KX728_RS02860		alpha/beta hydrolase-fold protein	-2.141639219	-4.412631335	2.45E-17	16.61
KX728_RS02850		ABC transporter ATP-binding protein	-2.160880975	-4.471878452	6.16E-13	12.21
KX728_RS02845		iron chelate uptake ABC transporter family permease subunit	-2.275281952	-4.840922348	4.65E-13	12.33
KX728_RS03850		alpha/beta hydrolase	-2.322085996	-5.000547272	7.96E-22	21.10
KX728_RS01725		ATP-binding cassette domain-containing protein	-2.420706113	-5.354330203	2.77E-10	9.56
KX728_RS02230		cation diffusion facilitator family transporter	-2.472033778	-5.548253775	6.88E-42	41.16
KX728_RS08145		transporter substrate-binding domain-containing protein	-2.486984516	-5.606049629	2.16E-14	13.67
KX728_RS08135		amino acid ABC transporter permease	-2.512371045	-5.70557009	2.16E-14	13.67
KX728_RS08130		amino acid ABC transporter ATP-binding protein	-2.527172942	-5.764409956	2.70E-15	14.57
KX728_RS02490	queC	7-cyano-7-deazaguanine synthase QueC	-2.541642165	-5.822513844	2.59E-28	27.59
KX728_RS02485	queD	6-carboxytetrahydropterin synthase QueD	-2.544797935	-5.835264028	1.27E-35	34.90
KX728_RS02480	queE	7-carboxy-7-deazaguanine synthase QueE	-2.571590225	-5.944643215	5.78E-28	27.24
KX728_RS08140		amino acid ABC transporter permease	-2.589941128	-6.020741296	3.32E-15	14.48
KX728_RS08880		rhodanese-related sulfurtransferase	-2.598490839	-6.056527396	5.53E-33	32.26
KX728_RS01720		siderophore ABC transporter substrate-binding protein	-2.60398491	-6.079635836	2.95E-17	16.53
KX728_RS08150		uroporphyrinogen decarboxylase family protein	-2.652419631	-6.287208604	2.69E-16	15.57
KX728_RS08155		hypothetical protein	-2.822062082	-7.071724551	2.36E-20	19.63
KX728_RS08160		uroporphyrinogen decarboxylase	-2.858915191	-7.254696149	6.29E-17	16.20

Supplemental Table 6. *Streptococcus oralis* 34 DEGs during coculture with *Streptococcus mutans* UA159 in either TY-, TY-Saliva, or both. Light grey,TY- only; Red, TY-Saliva; Dark blue, both.

TY				TY-Saliva		
Gene ID	Gene	Product		Geneid	Gene	Product
				KX728_RS00335		VOC family protein
				KX728_RS00735	tRNA-Tyr	
				KX728_RS00900	adhE	bifunctional acetaldehyde-CoA/alcohol dehydrogenase
KX728_RS02040	tRNA-Lys					
KX728_RS02275		hypothetical protein				
KX728_RS02565		hypothetical protein				
KX728_RS05075	sipA	Signal peptidase I (EC 3.4.21.89)				
KX728_RS05780		Late competence protein ComEA, DNA receptor				
KX728_RS06240	pdxS	Pyridoxal 5'-phosphate synthase (glutamine hydrolyzing)				
KX728_RS06245		NADH peroxidase (EC 1.11.1.1)				
KX728_RS06645	pyrF	Orotidine 5'-phosphate decarboxylase (EC 4.1.1.23)				
KX728_RS06650		Cell division protein FtsQ				
KX728_RS06750		Fructose-bisphosphate aldolase class II (EC 4.1.2.13)				
KX728_RS06770	vex2	ABC transporter, ATP-binding protein Vex2				
KX728_RS06780	tRNA-Leu					

Supplemental Table 7. Bacterial strains used in this study.

Species	Strain	Genotype or description	Antibiotic resistance	Reference or source
Actinomyces oris	WVU627			Lab Stock
Corynebacterium matruchotii	ATCC 14266			Lab Stock
Lactobacilllus casei	ATCC 4646			Lab Stock
Streptococcus cristatus	ATCC 51100			Lab Stock
Streptococcus gordonii	DL1			Lab Stock
Streptococcus mits	ATCC 49456			Lab Stock
Streptococcus mutans	UA159			Lab stock
	pMZ- / UA159	UA159 with pMZ plasmid integrated into genome. Serves as GFP(-) background control strain during fluorescent intensity-based competitive growth assays and as a "marked" *S. mutans* strain during competitive index assays	Kanamycin	Sheilds et al. 2019, Appl Environ Microbiol.
	pMZ-P*veg*::*gfp* / UA159	UA159 with pMZ plasmid integrated into genome harboring constitutively-active *gfp*. Serves as GFP(+) strain during fluorescent intensity-based competitive growth assays and used in coculture microscopy experiments	Kanamycin	Sheilds et al. 2019, Appl Environ Microbiol.
Streptococcus oralis	34			Lab Stock
	ΔKX728_03760	Mutation of gene KX728_03760 which is not actively expressed in *S. oralis* 34 by RNA-Seq. This strain is used within competitive index assays as a "marked" *S. oralis* strain	Spectinomycin	Lab Stock
Streptococcus sanguinis	SK36			Lab Stock
Streptococcus sobrinus	6715			Lab Stock

Supplemental Table 8. Catalog numbers of growth medium additives.

Category	Item	Vendor	Catalog Number
Human Saliva	Pooled Human Saliva	Innovative Research	IRHUSL250ML
Filter Unit	PES Membrane 0.22 µm Filter Unit	Millipore	SLGPR33RB
Bacteriological Mediums	Brain Heart Infusion	BD Difco	237500
	Tryptone	Fisher Bioreagents	BP1421
	Yeast Extract	Fisher Bioreagents	BP1422
	Potassium phosphate, dibasic	Sigma-Aldrich	P3786
Oil	Mineral Oil, Light	Fisher Chemical	O121
Carbohydrates	D-(+)-Cellobiose	Sigma-Aldrich	22150
	D-(-)-Fructose	Sigma-Aldrich	F0127
	D-(+)-Galactose	Sigma-Aldrich	G0750
	N-Acetyl-D-Glucosamine	Sigma-Aldrich	A8625
	D-(+)-Glucose	Sigma-Aldrich	G8270
	Lactose Monohydrate	Sigma-Aldrich	1.07657
	D-(+)-Mannose	Sigma-Aldrich	63580
	Sucrose	Sigma-Aldrich	S7903
Biofilm Imaging	Alexa Fluor 647-labeled dextran	Invitrogen	D22914
	12 well, glass bottom, black plates	Cellvis	P12-1.5H-N
	3% BSA in PBS	Thermo Scientific	J61655.AK
	B-DNA, Anti-dsDNA, 3519 DNA	Abcam	ab27156
	Z-DNA	Absolute Antibodies	AB00783-23.0
	Alexa Fluor 594-labeled goat anti-mouse IgG	Invitrogen	A32742
	Live Cell Imaging Solution, 1X	Invitrogen	A14291DJ
	Hoechst 33342	Thermo Scientific	62249
Metals	Iron(II) Sulfate Heptahydrate	Sigma-Aldrich	F8633
	Manganese(II) Sulfate Heptahydrate	Sigma-Aldrich	229784
	Zinc Sulfate Heptahydrate	Sigma-Aldrich	20251
CV Biofilms	Crystal Violet	Fisher Chemical	C581
	30% acetic acid solution	RICCA Chemical	1383032
Competitions	Costar 96 well black assay plates with clear bottom	Corning	3603
RNA Isolation	RNAprotect	Qiagen	76506
	QIAzol Lysis Reagent	Qiagen	79306
	Chloroform	Fisher Chemical	AC423550250
	RNeasy Mini Kit	Qiagen	74524
	2-mercaptoethanol	Sigma-Aldrich	M3148

	RNase-free DNase	Qiagen	79254
	Qubit RNA BR Assay Kit	Thermo Scientific	Q10210

Supplemental Table 9. Concentration* of inoculated bacterial strains during competitive competitions.
* Concentrations were determined based on the growth rate of individual species, such that the initial growth rate and entrance into exponential growth phase is matched to that of *Streptococcus mutans* pMZ-P*veg*::*gfp* / UA159 strain.

Strain Name	Concentration of Competitor (in comparision to *S. mutans*)	*S. mutans* Concentration
S. sp. A12	0.5x	1x
S. cristatus ATCC 51100	10x	1x
S. gordonii DL1	10% solution (1:10 dilution); 1x	1x
S. mitis ATCC 49456	1x	1x
S. oralis 34	1x	1x
S. sanguinis SK36	1x	1x
S. sobrinus 6715	2x	1x

Supplemental Table 10. Species identity of human saliva-derived bacterial isolates used in this study.

Strain Identifier	Species Identified by 16S Sequencing
SOSUI001	*Streptococcus salivarius*
SOSUI002	*Streptococcus salivarius*
SOSUI003	*Rothia mucilaginosa*
SOSUI004	*Rothia mucilaginosa*
SOSUI005	*Streptococcus infantis*
SOSUI006	*Streptococcus parasanguinis*
SOSUI007	*Rothia mucilaginosa*
SOSUI008	*Streptococcus salivarius*
SOSUI009	*Streptococcus parasanguinis*
SOSUI010	*Streptococcus salivarius*
SOSUI011	*Neisseria mucosa*
SOSUI012	*Streptococcus mitis*
SOSUI013	*Staphylococcus epidermidis*
SOSUI014	N/A
SOSUI015	*Streptococcus pseudopneumonia*
SOSUI016	*Granulicatella elegans*
SOSUI017	*Streptococcus salivarius*
SOSUI018	*Rothia dentocariosa*
SOSUI019	*Rothia dentocariosa*
SOSUI020	*Actinomyces oris*
SOSUI021	*Streptococcus mitis*
SOSUI022	*Actinomyces oris*
SOSUI023	N/A
SOSUI024	*Neisseria perflava*
SOSUI025	*Streptococcus sanguinis*
SOSUI026	*Actinomyces oris*
SOSUI027	*Streptococcus mitis*
SOSUI028	*Rothia dentocariosa*
SOSUI029	*Streptococcus mitis*
SOSUI030	*Streptococcus mitis*
SOSUI031	*Streptococcus mitis*
SOSUI032	*Streptococcus mitis*
SOSUI033	*Streptococcus infantis*
SOSUI034	*Streptococcus mitis*
SOSUI035	*Streptococcus sanguinis*
SOSUI036	*Streptococcus sanguinis*
SOSUI037	*Streptococcus mitis*

N/A = identity not available at the time of publication

Supplemental Table 11. RNA-Seq read counts assigned to each species. The first table expresses total number of raw read counts for each species per library, wile the second table is expressed in % of counts within the library.

Sample	Sample Name	SMU Counts	So34 Counts	Unassigned Counts	Total Counts
1	SMU_Mono_TYG_1	27,296,719	-	989,135	**28,288,718**
2	SMU_Mono_TYG_2	23,342,177	-	909,573	**24,252,194**
3	SMU_Mono_TYG_3	34,251,780	-	1,260,929	**35,521,179**
4	SMU_Mono_Saliva_1	28,233,732	-	1,345,373	**29,580,126**
5	SMU_Mono_Saliva_2	35,732,134	-	1,616,062	**37,362,518**
6	SMU_Mono_Saliva_3	29,436,944	-	1,461,315	**30,901,700**
7	So34_Mono_TYG_1	-	28,747,060	1,192,381	**29,947,676**
8	So34_Mono_TYG_2	-	24,127,536	892,143	**25,024,188**
9	So34_Mono_TYG_3	-	26,330,521	1,340,118	**27,674,072**
10	So34_Mono_Saliva_1	-	28,132,187	1,353,476	**29,489,220**
11	So34_Mono_Saliva_2	-	24,200,619	888,293	**25,091,932**
12	So34_Mono_Saliva_3	-	21,337,676	1,011,599	**22,355,198**
13	SMU_So34_TYG_1	23,414,430	37,277,444	1,991,434	**62,238,206**
14	SMU_So34_TYG_2	25,659,123	28,242,466	1,690,794	**54,978,976**
15	SMU_So34_TYG_3	24,139,040	37,960,448	2,180,094	**64,077,122**
16	SMU_So34_Saliva_1	26,855,427	41,849,131	1,413,320	**69,543,264**
17	SMU_So34_Saliva_2	26,413,072	40,543,444	1,797,724	**68,133,578**
18	SMU_So34_Saliva_3	25,252,549	47,380,963	1,941,747	**73,887,968**

Sample	Sample Name	SMU % Counts	So34 % Counts	Unassigned % Counts	Total % Counts
1	SMU_Mono_TYG_1	96.5%	0.0%	3.5%	100.0%
2	SMU_Mono_TYG_2	96.2%	0.0%	3.8%	100.0%
3	SMU_Mono_TYG_3	96.4%	0.0%	3.5%	100.0%
4	SMU_Mono_Saliva_1	95.4%	0.0%	4.5%	100.0%
5	SMU_Mono_Saliva_2	95.6%	0.0%	4.3%	100.0%
6	SMU_Mono_Saliva_3	95.3%	0.0%	4.7%	100.0%
7	So34_Mono_TYG_1	0.0%	96.0%	4.0%	100.0%
8	So34_Mono_TYG_2	0.0%	96.4%	3.6%	100.0%
9	So34_Mono_TYG_3	0.0%	95.1%	4.8%	100.0%
10	So34_Mono_Saliva_1	0.0%	95.4%	4.6%	100.0%
11	So34_Mono_Saliva_2	0.0%	96.4%	3.5%	100.0%
12	So34_Mono_Saliva_3	0.0%	95.4%	4.5%	100.0%
13	SMU_So34_TYG_1	37.6%	59.9%	3.2%	100.7%
14	SMU_So34_TYG_2	46.7%	51.4%	3.1%	101.1%
15	SMU_So34_TYG_3	38%	59%	3%	100.3%
16	SMU_So34_Saliva_1	38.6%	60.2%	2.0%	100.8%
17	SMU_So34_Saliva_2	38.8%	59.5%	2.6%	100.9%
18	SMU_So34_Saliva_3	34.2%	64.1%	2.6%	100.9%

Supplemental Table 12. Genome files used in this study.

Species	Isolate	Accession	Reference?	Source
Streptococcus mutans	UA159	NC_004350.2	Yes	NCBI GenBank
Streptococcus oralis	34	CP079724.1	No	NCBI GenBank
